# One-Pot NIS-Promoted Cyclization/Palladium-Catalyzed Carbonylation for the Selective Synthesis of HFIP Ester-Containing Indenes and Thiochromenes

**DOI:** 10.1021/acsorginorgau.5c00005

**Published:** 2025-02-21

**Authors:** Pengfei Ji, Xing-Feng Pan, Xinxin Qi, Xiao-Feng Wu

**Affiliations:** † School of Chemistry and Chemical Engineering, Key Laboratory of Surface & Interface Science of Polymer Materials of Zhejiang Province, Zhejiang Sci-Tech University, Hangzhou 310018, China; ‡ Dalian National Laboratory for Clean Energy, Dalian Institute of Chemical Physics, Chinese Academy of Sciences, Dalian 116023, Liaoning, China; § Leibniz-Institut für Katalyse e.V., Albert-Einstein-Straβe 29a, 18059 Rostock, Germany

**Keywords:** palladium catalyst, carbonylation, cyclization, cascade reaction, heterocycle synthesis, indene, thiochromene

## Abstract

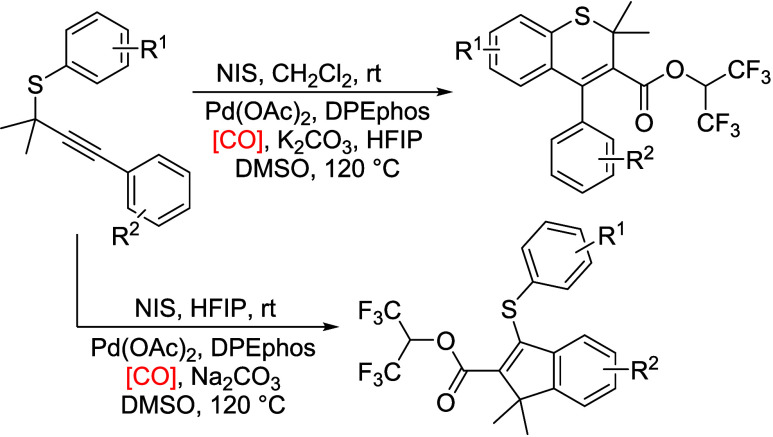

Practical and atom-economic procedures for the selective synthesis of HFIP ester-containing indenes/thiochromenes from the same propargylic thioethers and HFIP have been developed via one-pot NIS-promoted cyclization/palladium-catalyzed carbonylation. Solvent plays an important role in this transformation, and the reactions proceed selectively and efficiently to afford a variety of HFIP ester-containing indenes and thiochromenes in moderate to excellent yields. In addition, the use of formic acid as the CO source could avoid manipulation of toxic CO gas.

## Introduction

1,1,1,3,3,3-Hexafluoroisopropanol (HFIP), a versatile and superior solvent, has been widely used in organic synthesis^1−5^ and has gained much attention owing to its unique properties, including the ability to stabilize carbocationic species, high hydrogen bond donor strength,^6^ and low nucleophilicity.^7^ Taking advantage of these inherent capacities, the use of HFIP in organic chemistry continues to be an important task. Among HFIP-containing compounds, HFIP esters have been used as a type of useful building blocks and have made a particular impact in a variety of acylation reactions and asymmetric reactions.^4−17^ Although considerable effort for the synthesis of HFIP esters has been established, the formation of compounds with this unit is still limited. The exploration of straightforward and efficient strategies to access HFIP ester-containing molecules is still highly desired.

On the other hand, indenes and thiochromenes are valuable moieties present in a large number of natural products, pharmaceutical compounds, and synthetic intermediates. Indenes have broad applications in organic synthesis, medicinal chemistry, and material science due to their significant chemical, bioactive, and pharmacological properties.^18−21^ A number of synthetic methods have been explored, such as intramolecular electrophilic substitution reactions, transition-metal-catalyzed cyclizations, ring expansion, and contraction.^22,23^ Thiochromenes are, in general, synthesized by multistep reactions,^24−26^ as well as a series of enantioselective synthetic methods.^27−34^ Although numerous approaches have been reported for the construction of indenes and thiochromenes, to access these compounds with various functional groups and multisubstituents would be warmly welcomed.

Since the pioneer work by Heck and co-workers in 1974,^35−37^ palladium-catalyzed carbonylation reactions have become the most efficient strategies for the preparation of carbonyl-containing compounds and have attracted more and more attention from both academic and industrial fields.^38−43^ As a cheap and efficient *C*1 source, CO could be easily obtained from fossil fuel and biomass and plays an important role in carbonylation reaction. However, gaseous CO is colorless, odorless, and toxic, and the use of CO usually needs high-press equipment (Autoclave), which limits its applications in fine chemistry and lab use. Therefore, the exploration of convenient and atom-economic CO sources is of great interest.^44−51^ Recently, Sanz, Suárez-Pantiga, and their co-workers achieved NIS-promoted selective cyclization of propargylic thioethers to iodofuntionalizated indenes and 3-iodothiochromenes in good yields.^52−54^ Inspired by these achievements and with our continuous work on carbonylation reaction based on CO surrogates,^55−64^ as well as the advantage of HFIP esters, indenes and thiochromenes, herein, we wish to disclose a one-pot NIS-promoted cyclization/palladium-catalyzed carbonylation for the selective synthesis of HFIP ester-containing indenes and thiochromenes by applying formic acid as the CO source.

## Results and Discussion

Initially, (2-methyl-4-phenylbut-3-yn-2-yl)(*p*-tolyl)sulfane **1a** was selected as the model substrate, and the reaction was performed using a one-pot two-step method. Based on previous reports,^52−54^ solvent in the first step was very crucial for the synthesis of iodo-functionalized indene/thiothromene intermediates. Iodo-functionalized indenes were formed with HFIP as the solvent in the first step. Then the mixture was subjected under Pd(OAc)_2_/Xantphos-catalyzed system with K_2_CO_3_ as the base, formic acid as the CO source in 1,4-dioxane at 100 °C for 24 h, and no desired **3a** was observed (Table 1, entry 1). Solvents such as THF, CH_3_CN, toluene, DMF, and DMSO were tested (Table 1, entries 2–6). DMSO tends to be the best solvent, and product **3a** was obtained in 46% yield (Table 1, entry 6). The influence of base was then examined (Table 1, entries 7–11), and a better yield was detected with Na_2_CO_3_ as the base (Table 1, entry 7). Next, a series of ligands, such as DPEphos, Sphos, DPPF, DPPE, and DPPB, were investigated (Table 1, entries 12–16), and the yield of **3a** increased to 80% using DPEphos as the ligand (Table 1, entry 12). When the temperature increased to 120 °C, the yield of the expected product slightly improved to 82% (Table 1, entry 17). Furthermore, CH_2_Cl_2_ was employed as the only solvent in the first step, and HFIP was utilized in the second step, product **4a** was obtained in 30% yield (Table 1, entry 18). Notably, the reaction time of the first step is very important for the formation of iodo-functionalized thiochromene intermediates; a 62% yield of product **4a** was generated by prolonging the reaction time to 24 h (Table 1, entry 19). Finally, using K_2_CO_3_ as the base, the desired product was produced in 79% yield (Table 1, entry 20). An attempt with 1 mol % of catalyst loading was also carried out, but less than 10% yield of the desired product was detected.

**Table 1:**
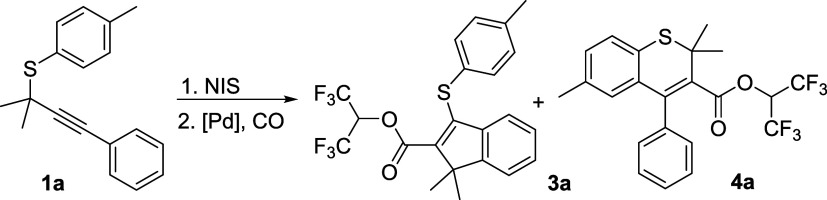
Optimization of the Reaction Conditions^a^

entry	ligand	base	solvent	yield **3a** (%)	yield **4a** (%)
1	xantphos	K_2_CO_3_	1,4-dioxane	0	
2	xantphos	K_2_CO_3_	THF	0	
3	xantphos	K_2_CO_3_	CH_3_CN	11	
4	xantphos	K_2_CO_3_	toluene	0	
5	xantphos	K_2_CO_3_	DMF	36	
6	xantphos	K_2_CO_3_	DMSO	46	
7	xantphos	Na_2_CO_3_	DMSO	69	
8	xantphos	Cs_2_CO_3_	DMSO	35	
9	xantphos	Na_3_PO_4_	DMSO	41	
10	xantphos	NaHCO_3_	DMSO	42	
11	xantphos	K_3_PO_4_	DMSO	47	
12	DPEphos	Na_2_CO_3_	DMSO	80	
13	Sphos	Na_2_CO_3_	DMSO	39	
14	DPPF	Na_2_CO_3_	DMSO	72	
15	DPPE	Na_2_CO_3_	DMSO	73	
16	DPPB	Na_2_CO_3_	DMSO	47	
17^b^	DPEphos	Na_2_CO_3_	DMSO	82	
18^b^^,^^c^	DPEphos	Na_2_CO_3_	HFIP/DMSO		30
19^b^^,^^c^^,^^d^	DPEphos	Na_2_CO_3_	HFIP/DMSO		62
20^b^^,^^c^^,^^d^	DPEphos	K_2_CO_3_	HFIP/DMSO		79

a Reaction conditions: (1) **1a** (0.2 mmol), NIS (0.3 mmol), HFIP (1 mL), 30 °C, 0.5 h. (2) Pd(OAc)_2_ (5 mol %), ligand (5 mol %), base (1.5 equiv), [CO] (HCOOH+Ac_2_O, 2.0 mmol), solvent (1 mL), 100 °C, 24 h. Isolated yields.

b (2) 120 °C.

c (1) CH_2_Cl_2_ (2 mL) instead of HFIP (1 mL), (2) HFIP (1 mL), DMSO (1 mL).

d (1) 24 h.

**Scheme 1:**
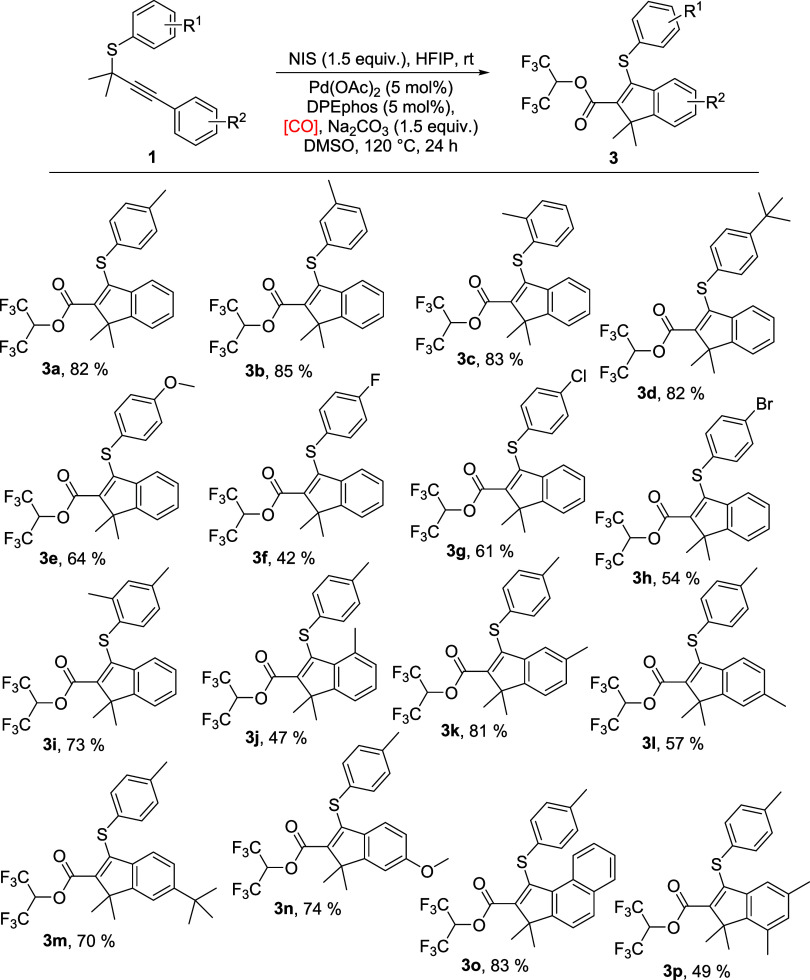
Synthesis of HFIP Ester-Containing Indenes Reaction conditions: (1) **1a** (0.2 mmol), NIS (0.3 mmol), HFIP (1 mL), 30 °C, 0.5 h. (2) Pd(OAc)_2_ (5 mol %), DPEphos (5 mol %), Na_2_CO_3_ (1.5 equiv), [CO] (HCOOH + Ac_2_O, 2.0 mmol), DMSO (1 mL), 120 °C, 24 h. Isolated yields.

With the optimal reaction conditions in hand, we first studied the synthesis of HFIP ester-containing indenes (Scheme 1). For aryl substituents attached to the S-atom, electron-donating groups, such as methyl, *tert*-butyl, and methoxy, were well tolerated to give the desired products in high yields (**3a**–**3e**). Halo groups such as fluoro, chloro, and bromo were then examined, and the target products were obtained in moderate yields (**3f**–**3h**). Dimethyl substituents also worked smoothly to provide product **3i** in a very good yield. For aryl alkynes, electron-donating groups, including methyl, *tert*-butyl, and methoxy, were compatible well to afford the corresponding products in moderate to high yields (**3j**–**3n**). Those substrates with *para-* and *meta-*groups provided the expected products in higher yields than *ortho*-substituent, probably due to the steric hindrance effect (**3k**, **3l** vs **3j**). Moreover, naphthalenyl and dimethyl substituents were tested, and the final products were isolated in 83 and 49% yields (**3o**, **3p**). However, no desired product was detected when the alkyne was heteroaryl group substituted.

**Scheme 2:**
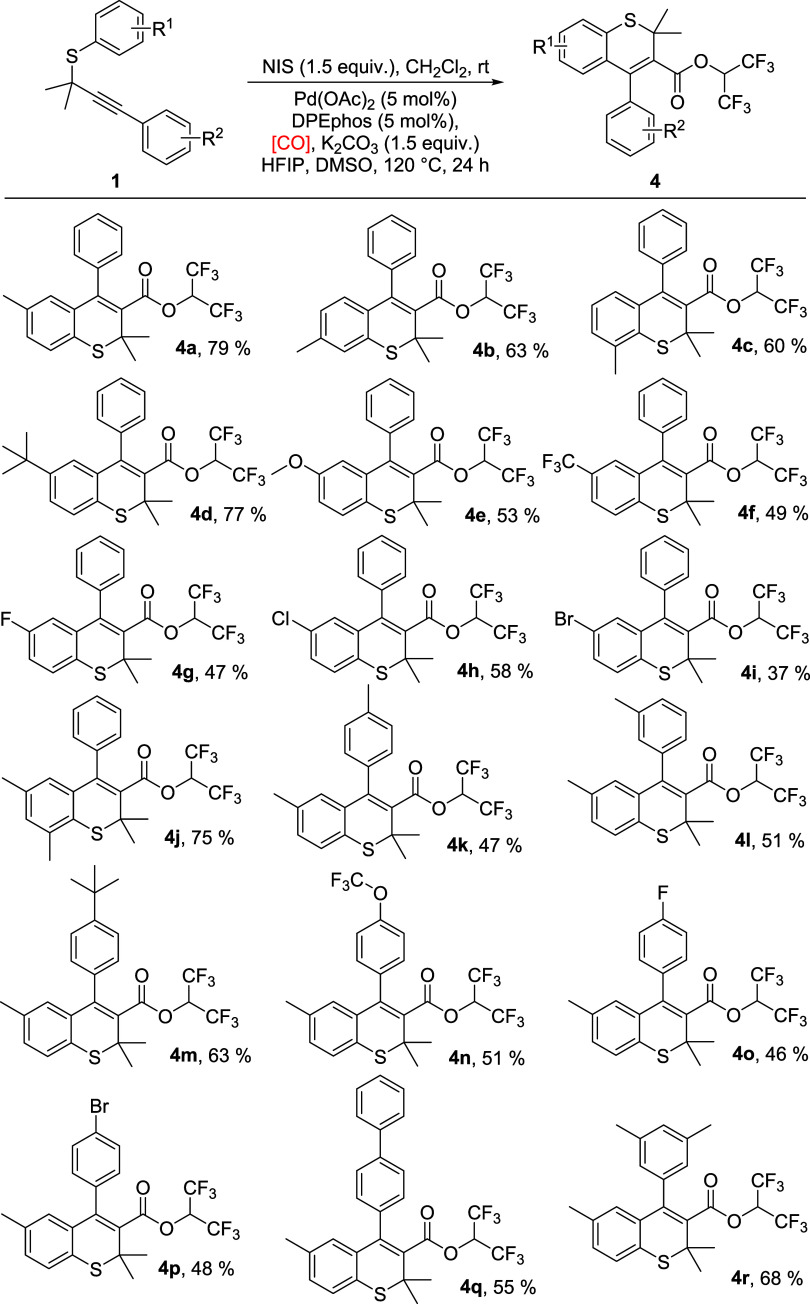
Synthesis of HFIP Ester-Containing Thiochromenes (1) **1** (0.2 mmol), NIS (0.3 mmol), CH_2_Cl_2_ (2 mL), 30 °C, 24 h. (2) Pd(OAc)_2_ (5 mol %), DPEphos (5 mol %), K_2_CO_3_ (1.5 equiv), [CO] (HCOOH+Ac_2_O, 2.0 mmol), HFIP (1 mL), DMSO (1 mL), 120 °C, 24 h. Isolated yields.

Subsequently, the generality of this carbonylation reaction toward the synthesis of HFIP ester-containing thiochromenes was investigated, and the results are summarized in Scheme 2. For aryl substituents on the S-atom, both electron-rich and electron-deficient groups were well tolerated, and the expected products were formed in moderate to good yields (**4a**–**4f**). Fluoro, chloro, and bromo groups were shown to be suitable substituents, resulting in the target products in moderate yields (**4g**–**4i**). Moreover, the dimethyl group was compatible as well and led to product **4j** in 75% yield. For aryl alkynes, this method allowed an array of HFIP ester-containing thiochromenes to be prepared with methyl, *tert*-butyl, and trifluoromethoxy substituents (**4k**–**4n**). In addition, fluoro, bromo, phenyl, and dimethyl groups could be introduced successfully under the standard reaction conditions; the corresponding products were obtained in moderate to good yields (**4o**–**4r**).

**Scheme 3:**
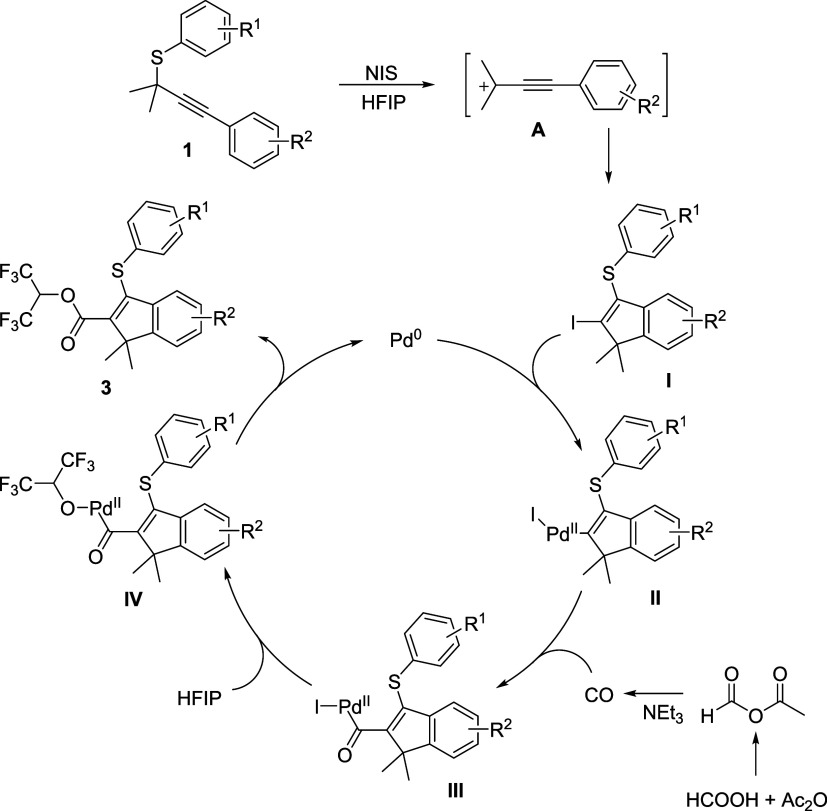
Plausible Mechanism for Indenes Synthesis

**Scheme 4:**
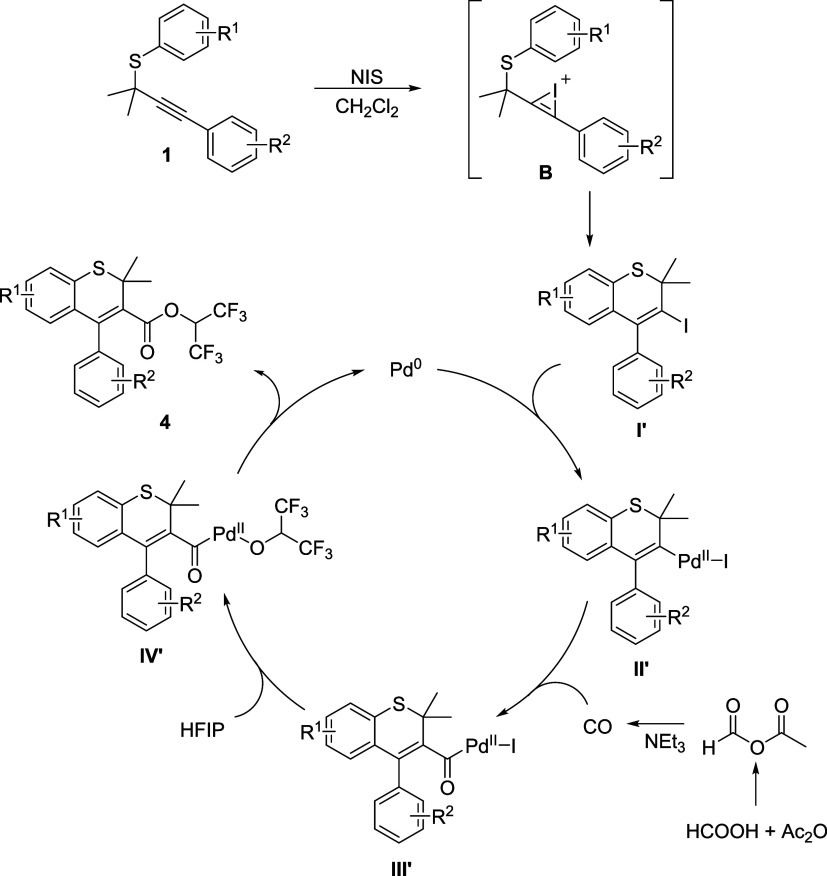
Plausible Mechanism for Thiochromenes Synthesis

Based on the above results and previous reports,^52−64^ two plausible reaction mechanisms are proposed in Schemes 3 and 4. Iodo-functionalized indenes (Scheme 3)/thiochromenes (Scheme 4) **I**/**I**′ were selectively synthesized from the same propargylic thioethers with NIS by employing HFIP/CH_2_Cl_2_ as the only solvent via different intermediates **A**/**B**. Then, an oxidation of Pd(0) with intermediates **I**/**I**′ to give complexes **II**/**II**′, followed by a CO (released from formic acid with acetic anhydride as the activator and promoted by NEt_3_) insertion and coordination to afford acylpalladium species **III**/**III**′. Next, a nucleophilic attack of HFIP to species **III**/**III′** occurs to deliver intermediates **IV**/**IV**′, which then undergo reductive elimination to afford target products **3**/**4**.

## Conclusions

In summary, one-pot NIS-promoted cyclization/palladium-catalyzed carbonylation reactions for the selective synthesis of HFIP ester-containing indenes and thiochromenes have been achieved. From the same propargylic thioethers and HFIP, by employing formic acid as the CO source, the reactions proceed selectively under CO-free conditions, affording a series of HFIP ester-containing indenes and thiochromenes in moderate to high yields.

## Experimental Section

### General Information

Unless otherwise noted, all reactions were carried out under a N_2_ atmosphere. All reagents were from commercial sources and used as received without further purification. All solvents were dry solvents. Column chromatography was performed on silica gel (200–300 meshes) using dichloromethane and ethyl acetate as eluent. NMR spectra were recorded on a Bruker Avance operating for ^1^H NMR at 400 MHz and ^13^C{^1^H} NMR at 101 MHz, and spectral data were reported in ppm relative to tetramethylsilane (TMS) as internal standard and CDCl_3_ (^1^H NMR δ 7.26, ^13^C{^1^H} NMR δ 77.16) as solvent. All coupling constants (*J*) are reported in Hz. The following abbreviations were used to describe peak splitting patterns when appropriate: s = singlet, d = doublet, dd = double doublet, ddd = double doublet of doublets, t = triplet, dt = double triplet, q = quatriplet, m = multiplet, and br = broad. Gas chromatography (GC) analyses were performed on a Shimadzu GC-2014C instrument equipped with an FID detector. Mass spectra (MS) were measured on a spectrometer by direct inlet at 70 eV. Mass spectroscopy data of the products were collected on an HRMS-TOF instrument or Waters TOFMS GCT Premier using EI or ESI ionization. Melting points were measured with a WRR digital point apparatus and not corrected.

### General Procedure for the Preparation of Propargyl Alcohols

To a mixture of aryl halide (1 equiv) and Et_3_N (3 equiv) in THF (0.5 M) were added PdCl_2_(PPh_3_)_2_ (2 mol %) and CuI (2 mol %) under a nitrogen atmosphere. After the reaction mixture was stirred for 5 min in ice baths, 2-methyl but-3-yn-2-ol (1.2 equiv) was added by a syringe. The reaction mixture was stirred at room temperature overnight. The resulting mixture was then poured into an aqueous saturated solution of NaCl (25 mL) and extracted with ethyl acetate (3 × 20 mL). The combined organic layers were washed with brine and dried over Na_2_SO_4_. The mixture was concentrated under reduced pressure and the residue was purified by column chromatography on silica gel (petroleum ether:ethyl acetate = 10:1) to get the corresponding propargyl alcohols.

### General Procedure for the Synthesis of Propargylic Thioethers

Thiol **S2** (1.3 equiv) and *p*-toluenesulfonic acid (5 mol %) were sequentially added to a solution of propargyl alcohol **S1** (1 equiv) in nitromethane (0.5 M). The mixture was allowed to stir at room temperature for 30 min to 2 h. Then, the reaction mixture was quenched by the addition of aqueous NaOH (20 mL) and CH_2_Cl_2_ (5 mL). The separated aqueous phase was extracted with CH_2_Cl_2_ (3  × 20 mL). The combined organic layers were dried over anhydrous Na_2_SO_4_, filtered, and concentrated under reduced pressure. The residue was purified by column chromatography on silica gel (petroleum ether:ethyl acetate = 200:1), affording the corresponding propargylic thioethers.

### General Procedure for the Synthesis of Indenes

**1** (0.2 mmol, 1.0 equiv), NIS (0.3 mmol, 1.5 equiv), and HFIP (1.0 mL) were added to an oven-dried tube (15 mL). The tube was sealed and the mixture was stirred at room temperature for 30 min. Subsequently, Pd(OAc)_2_ (5 mol %), DPEphos (5 mol %), and Na_2_CO_3_ (0.3 mmol, 1.5 equiv) were added to the reaction mixture which was then placed under vacuum and refilled with nitrogen three times quickly. Then DMSO (1.0 mL) was added to the reaction mixture via a syringe. A mixture of formic acid (2.0 mmol) and acetic anhydride (2.0 mmol) was stirred at 30 °C for 1.5 h and then added to the small inner tube with Et_3_N (2.0 mmol). The tube was sealed and the mixture was stirred at 120 °C (oil bath) for 24 h. After the reaction was completed, the reaction mixture was filtered and concentrated under a vacuum. The crude product was purified by column chromatography (petroleum ether:ethyl acetate = 100:1) on silica gel to afford the corresponding product **3**.

### General Procedure for the Synthesis of Thiochromenes

**1** (0.2 mmol, 1.0 equiv), NIS (0.3 mmol, 1.5 equiv), and CH_2_Cl_2_ (2.0 mL) were added to an oven-dried tube (15 mL) in ice baths. The tube was sealed and the mixture was stirred at room temperature for 24 h. Then CH_2_Cl_2_ was evaporated at room temperature, and Pd(OAc)_2_ (5 mol %), DPEphos (5 mol %), and K_2_CO_3_ (0.3 mmol, 1.5 equiv) were added to the reaction mixture which was then placed under vacuum and refilled with nitrogen three times. Next, DMSO (1.0 mL) and HFIP (1.0 mL) were added to the reaction mixture via a syringe. A mixture of formic acid (2.0 mmol) and acetic anhydride (2.0 mmol) was stirred at 30 °C for 1.5 h and then added to the small inner tube with Et_3_N (2.0 mmol). The tube was sealed, and the mixture was stirred at 120 °C (oil bath) for 24 h. After the reaction was completed, the reaction mixture was filtered and concentrated under a vacuum. The crude product was purified by column chromatography (petroleum ether:ethyl acetate = 100:1) on silica gel to afford the corresponding product **4**.

## Data Availability

The data underlying this study are available in the published article and its Supporting Information.

## Supporting Information Available

General comments, general procedures, analytic data, and NMR spectra of products (PDF)
