# Supplementary material for: One-Pot NIS-Promoted Cyclization/Palladium-Catalyzed Carbonylation for the Selective Synthesis of HFIP Ester-Containing Indenes and Thiochromenes
Source: ACS Org Inorg Au. 2025 Feb 21;5(2):156–63. doi: 10.1021/acsorginorgau.5c00005 (PMC11969275; doi:10.1021/acsorginorgau.5c00005)
Supplement: Supplementary file 1 — gg5c00005_si_001.pdf [file gg5c00005_si_001.pdf]

Supporting Information

One-Pot NIS-Promoted Cyclization/Palladium-Catalyzed Carbonylation for the Selective Synthesis of HFIP Esters-Containing Indenes and Thiochromenes

Pengfei Ji,† Xing-Feng Pan,† Xinxin Qi,*,† and Xiao-Feng Wu*,‡

[a] School of Chemistry and Chemical Engineering, Key Laboratory of Surface & Interface Science of Polymer Materials of Zhejiang Province, Zhejiang Sci-Tech University, Hangzhou, Zhejiang 310018, People's Republic of China
E-mail: xinxinqi@zstu.edu.cn

[b] Dalian National Laboratory for Clean Energy, Dalian Institute of Chemical Physics, Chinese Academy of Sciences, 116023, Dalian, Liaoning, China
E-mail: xwu2020@dicp.ac.cn

[c] Leibniz-Institut für Katalyse e.V., Albert-Einstein-Straße 29a, Rostock 18059, Germany
E-mail: xiao-feng.wu@catalysis.de

Table of Contents

1. General Information S2
2. General Procedure S3
2.1 General Procedure for the Synthesis of Starting Materials S3
2.1.1 General procedure for the Preparation of Propargyl Alcohols S3
2.1.2 General Procedure for the Synthesis of Propargylic Thioethers S3
2.2 General Procedure for the Synthesis of Products S4
3. Characterization Data of Products S6
4. Reference S27
5. Copy of ^{1}H and $^{13}C\{^{1}H\}$ NMR Spectra of Products S28

1. General Information

Unless otherwise noted, all reactions were carried out under N_2 atmosphere. All reagents were from commercial sources and used as received without further purification. All solvents were dry solvents. Column chromatography was performed on silica gel (200-300 meshes) using dichloromethane and ethyl acetate as eluent. NMR spectra were recorded on a Bruker Avance operating at for ^1H NMR at 400 MHz, $^{13}C\{^1H\}$ NMR at 101 MHz and spectral data were reported in ppm relative to tetramethylsilane (TMS) as internal standard and $CDCl_3$ (^1H NMR δ 7.26, $^{13}C\{^1H\}$ NMR δ 77.16) as solvent. All coupling constants (*J*) are reported in Hz. The following abbreviations were used to describe peak splitting patterns when appropriate: s = singlet, d = doublet, dd = double doublet, ddd = double doublet of doublets, t = triplet, dt = double triplet, q = quatriplet, m = multiplet, br = broad. Gas chromatography (GC) analyses were performed on a Shimadzu GC-2014C chromatograph equipped with a FID detector. Mass spectra (MS) were measured on spectrometer by direct inlet at 70 eV. Mass spectroscopy data of the products were collected on an HRMS-TOF instrument or Waters TOFMS GCT Premier using EI or ESI ionization. Melting points were measured with WRR digital point apparatus and not corrected.

2. General Procedure

2.1 General Procedure for the Synthesis of Starting Materials

2.1.1 General procedure for the Preparation of Propargyl Alcohols

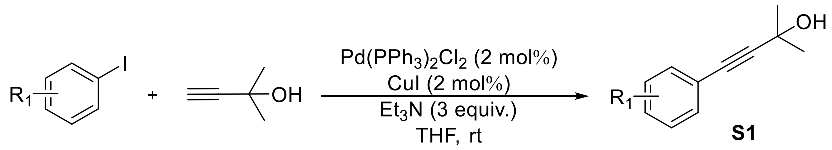

To a mixture of aryl halide (1 equiv.) and Et_3N (3 equiv.) in THF (0.5 M) were added $PdCl_2(PPh_3)_2$ (2 mol%) and CuI (2 mol%) under nitrogen atmosphere. After the reaction mixture was stirred for 5 min at ice baths, the 2-methyl but-3-yn-2-ol (1.2 equiv.) was added by a syringe. The reaction mixture was stirred at room temperature overnight. The resulting mixture was then poured into an aqueous saturated solution of NaCl (25 mL), extracted with ethyl acetate (3 × 20 mL). The combined organic layers were washed with brine, dried over Na_2SO_4, The mixture was concentrated under reduced pressure and the residue was purified by column chromatography on silica gel (petroleum ether : ethyl acetate = 10 : 1) to get the corresponding propargyl alcohols **S1**[1].

2.1.2 General Procedure for the Synthesis of Propargylic Thioethers

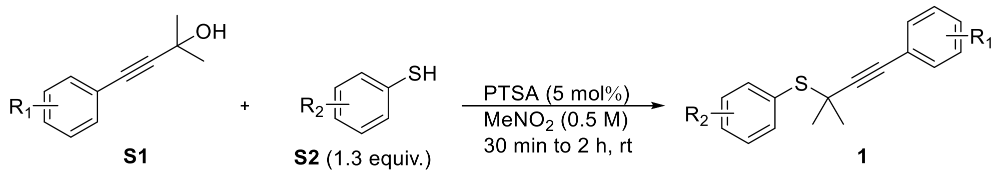

Thiol **S2** (1.3 equiv.) and *p*-toluenesulfonic acid (5 mol%) were sequentially added to a solution of propargyl alcohol **S1** (1 equiv.) in nitromethane (0.5 M). The mixture was allowed to stir at room temperature for 30 min to 2 h. Then, the reaction mixture was quenched by the addition of aqueous NaOH (20 mL) and CH_2Cl_2 (5 mL). The separated aqueous phase was extracted with CH_2Cl_2 (3 × 20 mL). The combined organic layers were dried over anhydrous Na_2SO_4, filtered, and concentrated under reduced pressure. The residue was purified by column chromatography on silica gel (petroleum ether : ethyl acetate = 200 : 1), affording the corresponding propargylic thioethers **1**[2].

2.2 General Procedure for the Synthesis of Products

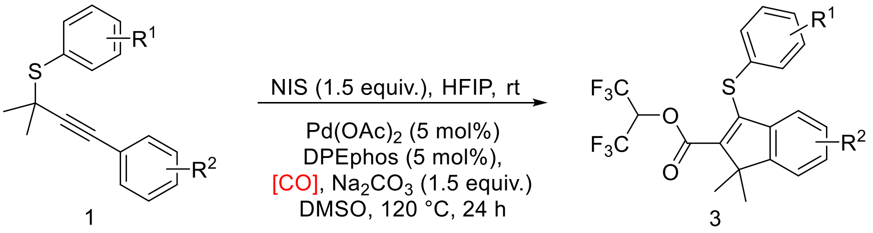

1 (0.2 mmol, 1.0 equiv.), NIS (0.3 mmol, 1.5 equiv.) and HFIP (1.0 mL) were added to an oven-dried tube (15 mL). The tube was sealed and the mixture was stirred at room temperature for 30 minutes. Subsequently, $Pd(OAc)_2$ (5 mol%), DPEphos (5 mol%), and Na_2CO_3 (0.3 mmol, 1.5 equiv.) were added to the reaction mixture which was then placed under vacuum and refilled with nitrogen for three times quickly. Then DMSO (1.0 mL) was added into the reaction mixture via a syringe. A mixture of formic acid (2.0 mmol) and acetic anhydride (2.0 mmol), which was stirred at 30 °C for 1.5 h and then added to the small inner tube with Et_3N (2.0 mmol). The tube was sealed and the mixture was stirred at 120 °C (oil bath) for 24 h. After the reaction was completed, the reaction mixture was filtered and concentrated under vacuum. The crude product was purified by column chromatography (petroleum ether : ethyl acetate = 100 : 1) on silica gel to afford the corresponding product **3**.

1a (266.5 mg, 1 mmol, 1.0 equiv.), NIS (1.5 mmol, 1.5 equiv.) and HFIP (5.0 mL) were added to an oven-dried tube (25 mL). The tube was sealed and the mixture was stirred at room temperature for 30 minutes. Subsequently, $Pd(OAc)_2$ (5 mol%), DPEphos (5 mol%), and Na_2CO_3 (1.5 mmol, 1.5 equiv.) were added to the reaction mixture which was then placed under vacuum and refilled with nitrogen for three times quickly. Then DMSO (5.0 mL) was added into the reaction mixture via a syringe. A mixture of formic acid (10.0 mmol) and acetic anhydride (10.0 mmol), which was stirred at 30 °C for 1.5 h and then added to the small inner tube with Et_3N (10.0 mmol). The tube was sealed and the mixture was stirred at 120 °C (oil bath) for 24 h. After the reaction was completed, the reaction mixture was filtered and concentrated under vacuum. The crude product was purified by column chromatography (petroleum ether : ethyl acetate = 100 : 1) on silica gel to afford the corresponding product **3a** in 80% yield (368 mg).

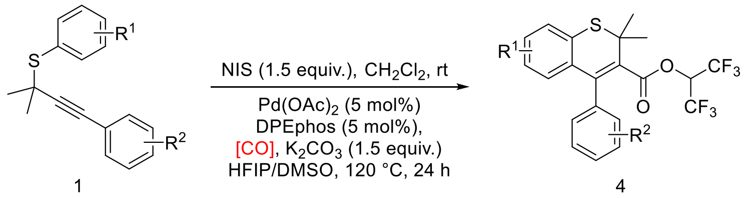

1 (0.2 mmol, 1.0 equiv.), NIS (0.3 mmol, 1.5 equiv.) and CH_2Cl_2 (2.0 mL) were added to an oven-dried tube (15 mL) at ice baths. The tube was sealed and the mixture was stirred at room temperature for 24 h. Then CH_2Cl_2 was evaporated at room temperature, $Pd(OAc)_2$ (5 mol%), DPEphos (5 mol%), and K_2CO_3 (0.3 mmol, 1.5 equiv.) were added to the reaction mixture which was then placed under vacuum and refilled with nitrogen for three times. Next, DMSO (1.0 mL) and HFIP (1.0 mL) were added into the reaction mixture via syringe. A mixture of formic acid (2.0 mmol) and acetic anhydride (2.0 mmol), which was stirred at 30 °C for 1.5 h and then added to the small inner tube with Et_3N (2.0 mmol). The tube was sealed and the mixture was stirred at 120 °C (oil bath) for 24 h. After the reaction was completed, the reaction mixture was filtered and concentrated under vacuum. The crude product was purified by column chromatography (petroleum ether : ethyl acetate = 100 : 1) on silica gel to afford the corresponding product **4**.

1a (266.5 mg, 1 mmol, 1.0 equiv.), NIS (1.5 mmol, 1.5 equiv.) and CH_2Cl_2 (10.0 mL) were added to an oven-dried tube (25 mL) at ice baths. The tube was sealed and the mixture was stirred at room temperature for 24 h. Then CH_2Cl_2 was evaporated at room temperature, $Pd(OAc)_2$ (5 mol%), DPEphos (5 mol%), and K_2CO_3 (1.5 mmol, 1.5 equiv.) were added to the reaction mixture which was then placed under vacuum and refilled with nitrogen for three times. Next, DMSO (5.0 mL) and HFIP (5.0 mL) were added into the reaction mixture via syringe. A mixture of formic acid (10.0 mmol) and acetic anhydride (10.0 mmol), which was stirred at 30 °C for 1.5 h and then added to the small inner tube with Et_3N (10.0 mmol). The tube was sealed and the mixture was stirred at 120 °C (oil bath) for 24 h. After the reaction was completed, the reaction mixture was filtered and concentrated under vacuum. The crude product was purified by column chromatography (petroleum ether : ethyl acetate = 100 : 1) on silica gel to afford the corresponding product **4a** in 75% yield (345 mg).

3. Characterization Data of Products

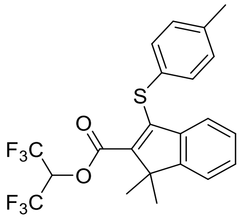

1,1,1,3,3,3-hexafluoropropan-2-yl 1,1-dimethyl-3-(*p*-tolylthio)-1*H*-indene-2-carboxylate (3a)

Upon completion the mixture was concentrated and purified via flash column chromatography (petroleum ether : ethyl acetate = 100 : 1) to give the titled product **3a** as a **yellow oil** (75.5 mg, 82%, 96% purity).

^{1}H NMR (400 MHz, CDCl$_3$) δ 7.42 (d, J = 8.1 Hz, 2H), 7.39 – 7.31 (m, 2H), 7.15 (d, J = 8.0 Hz, 2H), 7.01 – 6.97 (m, 1H), 6.62 (d, J = 8.0 Hz, 1H), 6.05 (hept, J = 6.1 Hz, 1H), 2.38 (s, 3H), 1.51 (s, 6H).

^{13}C{^{1}H} NMR (101 MHz, CDCl$_3$) δ 160.7, 155.8, 154.4, 138.8, 138.5, 135.5, 132.8, 130.1, 129.4, 128.4, 126.7, 124.7, 121.8, 120.76 (d, J = 284.7 Hz), 65.96 (p, J = 34.6 Hz), 49.8, 24.4, 21.2.

^{19}F NMR (376 MHz, CDCl$_3$) δ -72.80 (d, J = 5.6 Hz).

HRMS (ESI-TOF) m/z: [M+H]$^+$ Calcd. for $C_{22}H_{19}F_6O_2S^+$ 461.1004; Found 461.1007.

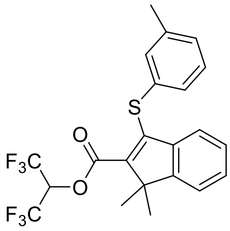

1,1,1,3,3,3-hexafluoropropan-2-yl 1,1-dimethyl-3-(*m*-tolylthio)-1*H*-indene-2-carboxylate (3b)

Upon completion the mixture was concentrated and purified via flash column chromatography (petroleum ether : ethyl acetate = 100 : 1) to give the titled product **3b** as a **yellow oil** (78.3 mg, 85%, 96% purity).

^{1}H NMR (400 MHz, CDCl$_3$) δ 7.37 (dd, J = 15.4, 9.2 Hz, 3H), 7.29 (d, J = 7.6 Hz,

1H), 7.22 (t, J = 7.6 Hz, 1H), 7.17 (d, J = 7.4 Hz, 1H), 7.00 (t, J = 7.5 Hz, 1H), 6.64 (d, J = 7.9 Hz, 1H), 6.05 (hept, J = 6.2 Hz, 1H), 2.33 (s, 3H), 1.52 (s, 6H).

$^{13}C\{^1H\}$ NMR (101 MHz, $CDCl_3$) δ 160.7, 155.7, 153.9, 139.2, 138.5, 135.9, 133.0, 131.8, 129.7, 129.4, 129.4, 129.1, 126.7, 124.7, 121.8, 120.72 (q, J = 282.1 Hz), 65.76 (p, J = 34.5 Hz), 49.9, 24.4, 21.3.

^{19}F NMR (376 MHz, $CDCl_3$) δ -72.72 (d, J = 6.0 Hz).

HRMS (ESI-TOF) m/z: $[M+H]^+$ Calcd. for $C_{22}H_{19}F_6O_2S^+$ 461.1004; Found 461.1008.

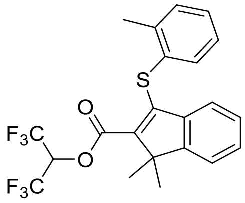

1,1,1,3,3,3-hexafluoropropan-2-yl 1,1-dimethyl-3-(*o*-tolylthio)-1*H*-indene-2-carboxylate (3c)

Upon completion the mixture was concentrated and purified via flash column chromatography (petroleum ether : ethyl acetate = 100 : 1) to give the titled product **3c** as a **yellow solid** (76.4 mg, 83%, 96% purity).

^1H NMR (400 MHz, $CDCl_3$) δ 7.51 (d, J = 7.7 Hz, 1H), 7.38 (d, J = 7.2 Hz, 1H), 7.35 – 7.29 (m, 2H), 7.27 (s, 1H), 7.18 – 7.14 (m, 1H), 6.98 – 6.94 (m, 1H), 6.51 (d, J = 8.0 Hz, 1H), 6.06 (hept, J = 6.2 Hz, 1H), 2.42 (s, 3H), 1.52 (s, 6H).

$^{13}C\{^1H\}$ NMR (101 MHz, $CDCl_3$) δ 160.8, 155.7, 154.6, 141.0, 138.7, 135.2, 134.2, 131.0, 130.9, 129.5, 129.3, 126.9, 126.8, 124.0, 121.8, 120.76 (q, J = 282.3 Hz), 66.11 (p, J = 34.5 Hz), 49.8, 24.4, 20.9.

^{19}F NMR (376 MHz, $CDCl_3$) δ -73.34 (d, J = 4.8 Hz).

HRMS (ESI-TOF) m/z: $[M+H]^+$ Calcd. for $C_{22}H_{19}F_6O_2S^+$ 461.1004; Found 461.1007.

M.p. 48.4– 48.9 °C

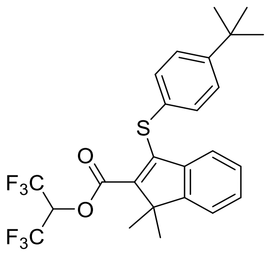

1,1,1,3,3,3-hexafluoropropan-2-yl 3-((4-(*tert*-butyl)phenyl)thio)-1,1-dimethyl-1*H*-indene-2-carboxylate (3d)

Upon completion the mixture was concentrated and purified via flash column chromatography (petroleum ether : ethyl acetate = 100 : 1) to give the titled product **3d** as a **yellow solid** (82.4 mg, 82%, 96% purity).

^{1}H NMR (400 MHz, CDCl$_3$) δ 7.46 (d, J = 8.4 Hz, 2H), 7.39 – 7.34 (m, 3H), 7.31 (d, J = 7.5 Hz, 1H), 6.96 (t, J = 7.6 Hz, 1H), 6.54 (d, J = 8.0 Hz, 1H), 6.03 (hept, J = 6.1 Hz, 1H), 1.51 (s, 6H), 1.33 (s, 9H).

^{13}C{^{1}H} NMR (101 MHz, CDCl$_3$) δ 160.7, 155.7, 152.2, 138.6, 134.6, 132.6, 129.4, 128.4, 126.7, 126.4, 124.7, 121.8, 121.22 (d, J = 282.3 Hz), 66.09 (p, J = 34.7 Hz), 49.8, 34.7, 31.2, 29.7, 24.4.

^{19}F NMR (376 MHz, CDCl$_3$) δ -73.46 (dd, J = 98.8, 69.5 Hz).

HRMS (ESI-TOF) m/z: $[M+H]^+$ Calcd. for $C_{25}H_{25}F_6O_2S^+$ 503.1474; Found 503.1476.

M.p. 64.4 – 65.1 °C

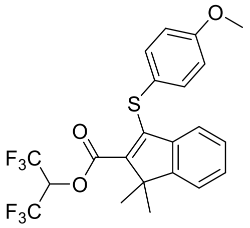

1,1,1,3,3,3-hexafluoropropan-2-yl 3-((4-methoxyphenyl)thio)-1,1-dimethyl-1*H*-indene-2-carboxylate (3e)

Upon completion the mixture was concentrated and purified via flash column chromatography (petroleum ether : ethyl acetate = 100 : 1) to give the titled product **3e** as a **yellow oil** (61.0 mg, 64%, 96% purity).

^{1}H NMR (400 MHz, CDCl$_3$) δ 7.51 (d, J = 8.5 Hz, 2H), 7.38 (d, J = 8.0 Hz, 1H), 7.33 (t, J = 7.4 Hz, 1H), 6.98 (t, J = 7.6 Hz, 1H), 6.90 (d, J = 8.6 Hz, 2H), 6.53 (d, J = 8.0

Hz, 1H), 6.06 (hept, J = 6.2 Hz, 1H), 3.85 (s, 3H), 1.50 (s, 6H).

^{13}C{^{1}H} NMR (101 MHz, CDCl3) δ 160.8, 160.4, 155.9, 138.4, 135.1, 134.0, 132.7, 129.4, 126.7, 124.7, 121.8, 120.77 (d, J = 283.1 Hz), 115.0, 114.6, 65.71 (p, J = 34.6 Hz), 55.4, 49.5, 24.4.

^{19}F NMR (376 MHz, CDCl3) δ -72.80 (d, J = 5.9 Hz).

HRMS (ESI-TOF) m/z: $[M+H]^+$ Calcd. for $C_{22}H_{19}F_6O_3S^+$ 477.0954; Found 477.0954.

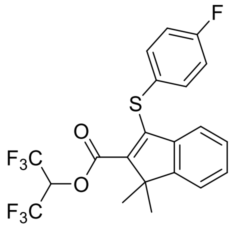

1,1,1,3,3,3-hexafluoropropan-2-yl 3-((4-fluorophenyl)thio)-1,1-dimethyl-1*H*-indene-2-carboxylate (3f)

Upon completion the mixture was concentrated and purified via flash column chromatography (petroleum ether : ethyl acetate = 100 : 1) to give the titled product **3f** as a **yellow oil** (39.0 mg, 42%, 96% purity).

^{1}H NMR (400 MHz, CDCl3) δ 7.51 (dd, J = 8.6, 5.3 Hz, 2H), 7.40 (d, J = 7.5 Hz, 1H), 7.35 (t, J = 7.4 Hz, 1H), 7.08 – 7.01 (m, 3H), 6.61 (d, J = 7.9 Hz, 1H), 6.04 (hept, J = 6.1 Hz, 1H), 1.51 (s, 6H).

^{13}C{^{1}H} NMR (101 MHz, CDCl3) δ 160.7, 157.52 (d, J = 247.0 Hz), 155.8, 138.2, 134.7, 134.6, 129.6, 126.8, 124.4, 121.9, 120.70 (d, J = 284.3 Hz), 116.60 (d, J = 22.1 Hz), 66.15 (p, J = 34.8 Hz), 50.0, 24.3.

^{19}F NMR (376 MHz, CDCl3) δ -73.33 (s), -112.42 (s).

HRMS (ESI-TOF) m/z: $[M+H]^+$ Calcd. for $C_{21}H_{16}F_7O_2S^+$ 465.0754; Found 465.0756.

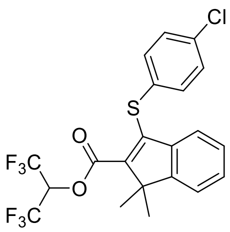

1,1,1,3,3,3-hexafluoropropan-2-yl 3-((4-chlorophenyl)thio)-1,1-dimethyl-1*H*-ind-ene-2-carboxylate (3g)

Upon completion the mixture was concentrated and purified via flash column chromatography (petroleum ether : ethyl acetate = 100 : 1) to give the titled product **3g** as a **yellow oil** (58.7 mg, 61%, 96% purity).

^{1}H NMR (400 MHz, CDCl$_3$) δ 7.41 (d, J = 8.4 Hz, 3H), 7.36 (s, 1H), 7.30 (d, J = 8.5 Hz, 2H), 7.06 (t, J = 7.5 Hz, 1H), 6.73 (d, J = 7.9 Hz, 1H), 6.03 (hept, J = 6.2 Hz, 1H), 1.52 (s, 6H).

^{13}C{^{1}H} NMR (101 MHz, CDCl$_3$) δ 160.6, 155.6, 138.1, 134.5, 133.2, 132.8, 131.1, 129.6, 129.5, 129.3, 126.9, 124.5, 123.25 (d, J = 285.2 Hz), 121.9, 66.20 (p, J = 34.1 Hz), 50.2, 24.3.

^{19}F NMR (376 MHz, CDCl$_3$) δ -73.32 (d, J = 5.2 Hz).

HRMS (ESI-TOF) m/z: [M+H]$^+$ Calcd. for $C_{21}H_{16}F_6O_2S^+$ 481.0458; Found 481.0461.

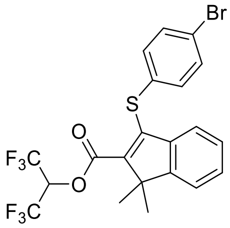

1,1,1,3,3,3-hexafluoropropan-2-yl 3-((4-bromophenyl)thio)-1,1-dimethyl-1*H*-ind-ene-2-carboxylate (3h)

Upon completion the mixture was concentrated and purified via flash column chromatography (petroleum ether : ethyl acetate = 100 : 1) to give the titled product **3h** as a **yellow oil** (56.7 mg, 54%, 96% purity).

^{1}H NMR (400 MHz, CDCl$_3$) δ 7.45 (d, J = 8.4 Hz, 2H), 7.40 (s, 1H), 7.33 (d, J = 8.3 Hz, 3H), 7.07 (t, J = 7.5 Hz, 1H), 6.75 (d, J = 7.9 Hz, 1H), 6.03 (hept, J = 6.2 Hz, 1H), 1.52 (s, 6H).

$^{13}C\{^1H\}$ NMR (101 MHz, CDCl$_3$) δ 160.6, 155.6, 138.1, 133.3, 132.9, 132.4, 132.2, 131.8, 129.6, 126.9, 124.5, 122.4, 121.9, 120.60 (d, J = 285.6 Hz), 50.3, 66.04 (p, J = 34.4 Hz), 24.3.

^{19}F NMR (376 MHz, CDCl$_3$) δ -72.80 (d, J = 6.0 Hz).

HRMS (ESI-TOF) m/z: $[M+H]^+$ Calcd. for $C_{21}H_{16}BrF_6O_2S^+$ 524.9953; Found 524.9953.

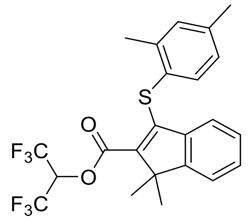

1,1,1,3,3,3-hexafluoropropan-2-yl 3-((2,4-dimethylphenyl)thio)-1,1-dimethyl-1*H*-indene-2-carboxylate (3i)

Upon completion the mixture was concentrated and purified via flash column chromatography (petroleum ether : ethyl acetate = 100 : 1) to give the titled product **3i** as a **yellow oil** (69.3 mg, 73%, 96% purity).

^1H NMR (400 MHz, CDCl$_3$) δ 7.44 (d, J = 7.8 Hz, 1H), 7.37 (d, J = 7.5 Hz, 1H), 7.32 (t, J = 7.4 Hz, 1H), 7.09 (s, 1H), 6.97 (dd, J = 16.0, 7.9 Hz, 2H), 6.51 (d, J = 8.0 Hz, 1H), 6.07 (hept, J = 6.2 Hz, 1H), 2.36 (s, 6H), 1.50 (s, 6H).

$^{13}C\{^1H\}$ NMR (101 MHz, CDCl$_3$) δ 160.8, 155.7, 155.6, 141.1, 139.7, 138.8, 134.6, 134.1, 131.7, 129.5, 127.7, 127.2, 126.8, 124.0, 121.8, 120.76 (q, J = 282.6, Hz), 65.86 (p, J = 34.6 Hz), 49.6, 24.4, 21.2, 20.9.

^{19}F NMR (376 MHz, CDCl$_3$) δ -72.74 (d, J = 5.9 Hz).

HRMS (ESI-TOF) m/z: $[M+H]^+$ Calcd. for $C_{23}H_{21}F_6O_2S^+$ 475.1161; Found 475.1165.

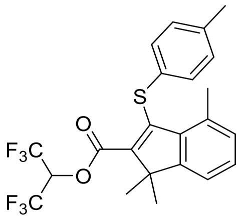

1,1,1,3,3,3-hexafluoropropan-2-yl 1,1,4-trimethyl-3-(*p*-tolylthio)-1*H*-indene-2-carboxylate (3j)

Upon completion the mixture was concentrated and purified via flash column chromatography (petroleum ether : ethyl acetate = 100 : 1) to give the titled product **3j** as a **yellow oil** (44.6 mg, 47%, 96% purity).

^{1}H NMR (400 MHz, $CDCl_3$) δ 7.30 (d, J = 7.4 Hz, 1H), 7.27 (s, 1H), 7.08 (s, 1H), 7.05 (d, J = 3.7 Hz, 3H), 7.03 (s, 1H), 5.86 (hept, J = 6.2 Hz, 1H), 2.55 (s, 3H), 2.28 (s, 3H), 1.51 (s, 6H).

^{13}C{^{1}H} NMR (101 MHz, $CDCl_3$) δ 160.8, 155.2, 148.7, 144.1, 137.2, 136.2, 135.5, 132.5, 130.7, 130.0, 129.2, 127.8, 120.56 (d, J = 282.6 Hz), 119.5, 65.77 (p, J = 34.9 Hz), 50.6, 24.6, 20.9, 20.3.

^{19}F NMR (376 MHz, $CDCl_3$) δ -72.85 (d, J = 5.8 Hz).

HRMS (ESI-TOF) m/z: $[M+H]^+$ Calcd. for $C_{23}H_{21}F_6O_2S^+$ 475.1161; Found 475.1161.

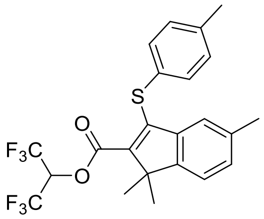

1,1,1,3,3,3-hexafluoropropan-2-yl 1,1,5-trimethyl-3-(*p*-tolylthio)-1*H*-indene-2-carboxylate (3k)

Upon completion the mixture was concentrated and purified via flash column chromatography (petroleum ether : ethyl acetate = 100 : 1) to give the titled product **3k** as a **yellow solid** (76.9 mg, 81%, 96% purity).

^{1}H NMR (400 MHz, $CDCl_3$) δ 7.38 (dd, J = 45.8, 7.9 Hz, 2H), 7.24 (s, 1H), 7.17 (d, J = 8.4 Hz, 1H), 7.11 (dd, J = 16.6, 8.2 Hz, 1H), 6.31 (s, 1H), 6.05 (hept, J = 6.2 Hz, 1H), 2.46 (d, J = 53.0 Hz, 3H), 2.21 (d, J = 111.4 Hz, 3H), 1.56 (d, J = 56.2 Hz, 6H).

$^{13}C\{^1H\}$ NMR (101 MHz, CDCl3) δ 160.2, 149.6, 148.9, 138.7, 137.7, 137.05 (d, J = 5.6 Hz), 132.9, 130.9, 129.8, 129.8, 129.2, 127.4, 126.9, 121.60 (d, J = 130.5 Hz), 120.45 (d, J = 285.1 Hz), 119.2, 66.18 (p, J = 34.4 Hz), 55.20 (d, J = 68.9 Hz), 25.6, 21.84 (d, J = 46.6 Hz), 21.10 (d, J = 11.0 Hz), 18.96.

^{19}F NMR (376 MHz, CDCl3) δ -72.74 (dd, J = 12.4, 6.1 Hz).

HRMS (ESI-TOF) m/z: $[M+H]^+$ Calcd. for $C_{23}H_{21}F_6O_2S^+$ 475.1161; Found 475.1161.

M.p. 69.8 – 71.4 ºC

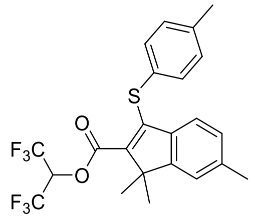

1,1,1,3,3,3-hexafluoropropan-2-yl 1,1,6-trimethyl-3-(*p*-tolylthio)-1*H*-indene-2-carboxylate (3l)

Upon completion the mixture was concentrated and purified via flash column chromatography (petroleum ether : ethyl acetate = 100 : 1) to give the titled product **3l** as a **yellow solid** (54.1 mg, 57%, 96% purity).

1H NMR (400 MHz, CDCl3) δ 7.43 (d, J = 7.9 Hz, 2H), 7.15 (d, J = 8.4 Hz, 3H), 6.79 (d, J = 8.1 Hz, 1H), 6.43 (d, J = 8.1 Hz, 1H), 6.05 (hept, J = 6.1 Hz, 1H), 2.38 (s, 3H), 2.35 (s, 3H), 1.49 (s, 6H).

$^{13}C\{^1H\}$ NMR (101 MHz, CDCl3) δ 160.8, 156.2, 155.2, 140.0, 138.9, 135.9, 134.0, 133.1, 130.1, 128.3, 127.7, 124.5, 122.4, 120.76 (q, J = 282.9 Hz), 65.83 (p, J = 34.6 Hz), 49.4, 24.5, 21.7, 21.3.

^{19}F NMR (376 MHz, CDCl3) δ -73.33 (d, J = 5.2 Hz).

HRMS (ESI-TOF) m/z: $[M+H]^+$ Calcd. for $C_{23}H_{21}F_6O_2S^+$ 475.1161; Found 475.1165.

M.p. 51.5 – 52.1 ºC

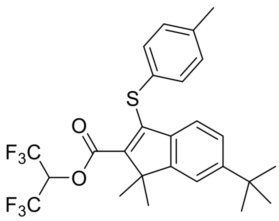

1,1,1,3,3,3-hexafluoropropan-2-yl 6-(*tert*-butyl)-1,1-dimethyl-3-(*p*-tolylthio)-1*H*-indene-2-carboxylate (3m)

Upon completion the mixture was concentrated and purified via flash column chromatography (petroleum ether : ethyl acetate = 100 : 1) to give the titled product **3m** as a **yellow oil** (72.3 mg, 70%, 96% purity).

^{1}H NMR (400 MHz, CDCl$_3$) δ 7.46 (d, J = 7.9 Hz, 1H), 7.35 (s, 2H), 7.17 (d, J = 7.9 Hz, 2H), 7.00 (dd, J = 8.3, 1.4 Hz, 1H), 6.41 (d, J = 8.4 Hz, 1H), 6.05 (hept, J = 6.2 Hz, 1H), 2.41 (s, 3H), 1.49 (s, 6H), 1.29 (s, 9H).

^{13}C{^{1}H} NMR (101 MHz, CDCl$_3$) δ 160.8, 155.9, 153.3, 139.1, 135.9, 133.7, 133.4, 130.1, 128.0, 124.2, 124.1, 120.77 (q, J = 284.8 Hz), 118.4, 65.83 (p, J = 34.5 Hz), 49.5, 35.1, 31.3, 24.6, 21.3.

^{19}F NMR (376 MHz, CDCl$_3$) δ -72.71 (d, J = 6.1 Hz).

HRMS (ESI-TOF) m/z: [M+H]$^+$ Calcd. for $C_{26}H_{27}F_6O_2S^+$ 517.1630; Found 517.1635.

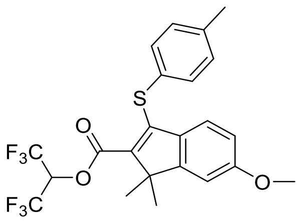

1,1,1,3,3,3-hexafluoropropan-2-yl 6-methoxy-1,1-dimethyl-3-(*p*-tolylthio)-1*H*-indene-2-carboxylate (3n)

Upon completion the mixture was concentrated and purified via flash column chromatography (petroleum ether : ethyl acetate = 100 : 1) to give the titled product **3n** as a **yellow solid** (72.6 mg, 74%, 96% purity).

^{1}H NMR (400 MHz, CDCl$_3$) δ 7.44 (d, J = 8.0 Hz, 2H), 7.16 (d, J = 7.9 Hz, 2H), 6.86 (d, J = 2.0 Hz, 1H), 6.56 – 6.34 (m, 2H), 6.05 (hept, J = 6.0 Hz, 1H), 3.80 (s, 3H), 2.39 (s, 3H), 1.49 (s, 6H).

$^{13}C\{^1H\}$ NMR (101 MHz, $CDCl_3$) δ 161.2, 160.6, 158.6, 155.3, 139.0, 133.2, 132.6, 131.3, 130.1, 128.3, 126.0, 125.0, 120.81 (d, J = 281.8 Hz), 112.7, 107.2, 65.79 (p, J = 34.3 Hz), 55.5, 49.4, 24.7, 21.3 .

^{19}F NMR (376 MHz, $CDCl_3$) δ -72.82 (d, J = 6.1 Hz).

HRMS (ESI-TOF) m/z: $[M+H]^+$ Calcd. for $C_{23}H_{21}F_6O_3S^+$ 491.1110; Found 491.1111.

M.p. 81.3 – 81.8 °C

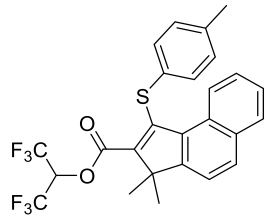

1,1,1,3,3,3-hexafluoropropan-2-yl 3,3-dimethyl-1-(*p*-tolylthio)-3*H*-cyclopenta[a]naphthalene-2-carboxylate (3o)

Upon completion the mixture was concentrated and purified via flash column chromatography (petroleum ether : ethyl acetate = 100 : 1) to give the titled product **3o** as a **yellow oil** (84.7 mg, 83%, 96% purity).

^1H NMR (400 MHz, $CDCl_3$) δ 9.25 – 9.23 (m, 1H), 7.96 (d, J = 8.3 Hz, 1H), 7.87 – 7.86 (m, 1H), 7.57 (d, J = 8.4 Hz, 1H), 7.44 (dd, J = 6.3, 3.2 Hz, 2H), 7.13 (d, J = 8.1 Hz, 2H), 6.99 (d, J = 7.7 Hz, 2H), 5.94 (hept, J = 6.2 Hz, 1H), 2.22 (s, 3H), 1.59 (s, 6H).

$^{13}C\{^1H\}$ NMR (101 MHz, $CDCl_3$) δ 160.3, 154.9, 149.3, 144.5, 136.3, 133.5, 133.2, 132.0, 131.5, 130.0, 129.2, 128.8, 128.1, 126.9, 125.6, 124.5, 120.61 (q, J = 282.6 Hz), 119.5, 65.91 (p, J = 34.7 Hz), 51.0, 23.6, 20.9.

^{19}F NMR (376 MHz, $CDCl_3$) δ -72.74 (d, J = 5.8 Hz).

HRMS (ESI-TOF) m/z: $[M+H]^+$ Calcd. for $C_{26}H_{21}F_6O_2S^+$ 511.1161; Found 511.1166.

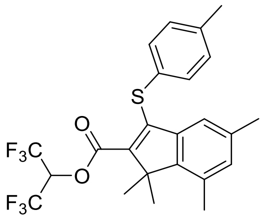

1,1,1,3,3,3-hexafluoropropan-2-yl 1,1,5,7-tetramethyl-3-(*p*-tolylthio)-1*H*-indene-2-carboxylate (3p)

Upon completion the mixture was concentrated and purified via flash column chromatography (petroleum ether : ethyl acetate = 100 : 1) to give the titled product **3p** as a **yellow oil** (47.8 mg, 49%, 96% purity).

^{1}H NMR (400 MHz, CDCl$_3$) δ 7.34 (d, J = 8.0 Hz, 2H), 7.12 (d, J = 7.9 Hz, 2H), 6.92 (s, 1H), 6.48 (s, 1H), 6.03 (hept, J = 6.2 Hz, 1H), 2.48 (s, 3H), 2.36 (s, 3H), 2.07 (s, 3H), 1.60 (s, 6H).

^{13}C{^{1}H} NMR (101 MHz, CDCl$_3$) δ 160.6, 152.5, 149.3, 139.6, 138.1, 138.0, 136.3, 133.1, 132.6, 131.9, 129.9, 129.6, 129.3, 128.8, 123.1, 120.72 (d, J = 282.9 Hz), 66.06 (p, J = 34.7 Hz), 51.0, 21.1, 21.0, 19.0.

^{19}F NMR (376 MHz, CDCl$_3$) δ -72.75 (d, J = 5.9 Hz).

HRMS (ESI-TOF) m/z: [M+H]$^+$ Calcd. for $C_{24}H_{23}F_6O_2S^+$ 489.1317; Found 489.1320.

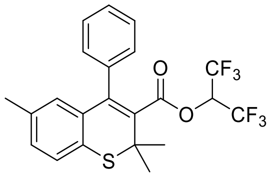

1,1,1,3,3,3-hexafluoropropan-2-yl 2,2,6-trimethyl-4-phenyl-2*H*-thiochromene-3-carboxylate (4a)

Upon completion the mixture was concentrated and purified via flash column chromatography (petroleum ether : ethyl acetate = 100 : 1) to give the titled product **4a** as a **yellow solid** (72.7 mg, 79%, 96% purity).

^{1}H NMR (400 MHz, CDCl$_3$) δ 7.69 (d, J = 7.6 Hz, 1H), 7.40 (d, J = 8.0 Hz, 2H), 7.27 (d, J = 4.7 Hz, 3H), 7.13 (d, J = 7.9 Hz, 2H), 5.95 (hept, J = 6.1 Hz, 1H), 2.35 (s, 3H), 1.30 (s, 6H).

^{13}C{^{1}H} NMR (101 MHz, CDCl$_3$) δ 160.1, 152.3, 138.8, 136.9, 133.1, 129.8, 127.8,

127.4, 126.1, 125.4, 121.5, 121.2, 120.57 (q, J = 282.4 Hz), 66.18 (p, J = 34.9 Hz), 55.2, 25.5, 21.2.

^{19}F NMR (376 MHz, $CDCl_3$) δ -73.27 (d, J = 4.7 Hz).

HRMS (ESI-TOF) m/z: $[M+H]^+$ Calcd. for $C_{22}H_{19}F_6O_2S^+$ 461.1004; Found 461.1007.

M.p. 81.2 – 81.7 °C

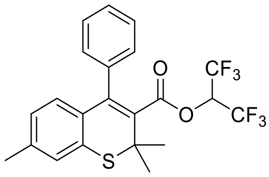

1,1,1,3,3,3-hexafluoropropan-2-yl 2,2,7-trimethyl-4-phenyl-2*H*-thiochromene-3-carboxylate (4b)

Upon completion the mixture was concentrated and purified via flash column chromatography (petroleum ether : ethyl acetate = 100 : 1) to give the titled product **4b** as a **yellow oil** (58.0 mg, 63%, 96% purity).

^{1}H NMR (400 MHz, $CDCl_3$) δ 7.71 (d, J = 7.4 Hz, 1H), 7.35 – 7.30 (m, 3H), 7.28 (s, 2H), 7.19 (t, J = 7.9 Hz, 1H), 7.10 (d, J = 7.5 Hz, 1H), 5.93 (hept, J = 6.1 Hz, 1H), 2.31 (s, 3H), 1.32 (s, 6H).

^{13}C{^{1}H} NMR (101 MHz, $CDCl_3$) δ 160.0, 152.3, 139.0, 136.9, 132.5, 131.7, 129.2, 129.0, 128.8, 127.4, 126.3, 121.7, 121.4, 120.53 (d, J = 284.3 Hz), 66.35 (p, J = 34.4 Hz), 55.1, 25.4, 21.2.

^{19}F NMR (376 MHz, $CDCl_3$) δ -72.79 (d, J = 5.9 Hz).

HRMS (ESI-TOF) m/z: $[M+H]^+$ Calcd. for $C_{22}H_{19}F_6O_2S^+$ 461.1004; Found 461.1007.

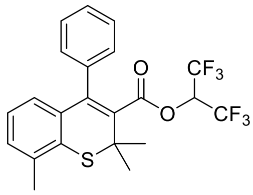

1,1,1,3,3,3-hexafluoropropan-2-yl 2,2,8-trimethyl-4-phenyl-2*H*-thiochromene-3-carboxylate (4c)

Upon completion the mixture was concentrated and purified via flash column chromatography (petroleum ether : ethyl acetate = 100 : 1) to give the titled product **4c** as a **yellow oil** (55.3 mg, 60%, 96% purity).

^{1}H NMR (400 MHz, CDCl$_3$) δ 7.70 (d, *J* = 7.6 Hz, 1H), 7.43 (d, *J* = 7.7 Hz, 1H), 7.34 – 7.28 (m, 3H), 7.23 (d, *J* = 4.2 Hz, 2H), 7.13 (dt, *J* = 8.6, 4.4 Hz, 1H), 5.91 (hept, *J* = 6.2 Hz, 1H), 2.47 (s, 3H), 1.30 (s, 6H).

^{13}C{^{1}H} NMR (101 MHz, CDCl$_3$) δ 160.0, 152.3, 140.9, 137.0, 135.7, 133.1, 130.9, 130.6, 128.8, 127.4, 126.6, 126.1, 121.5, 121.3, 120.55 (q, *J* = 283.8 Hz), 66.32 (p, *J* = 34.6 Hz), 55.2, 25.2, 20.9.

^{19}F NMR (376 MHz, CDCl$_3$) δ -72.73 (d, *J* = 5.8 Hz).

HRMS (ESI-TOF) m/z: $[M+H]^+$ Calcd. for $C_{22}H_{19}F_6O_2S^+$ 461.1004; Found 461.1005.

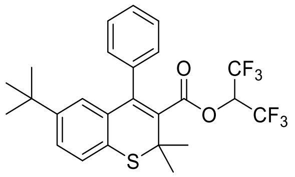

1,1,1,3,3,3-hexafluoropropan-2-yl 6-(*tert*-butyl)-2,2-dimethyl-4-phenyl-2*H*-thiochromene-3-carboxylate (4d)

Upon completion the mixture was concentrated and purified via flash column chromatography (petroleum ether : ethyl acetate = 100 : 1) to give the titled product **4d** as a **yellow oil** (77.4 mg, 77%, 96% purity).

^{1}H NMR (400 MHz, CDCl$_3$) δ 7.69 (d, *J* = 7.5 Hz, 1H), 7.41 (d, *J* = 8.5 Hz, 2H), 7.33 (d, *J* = 8.4 Hz, 3H), 7.29 (dd, *J* = 6.2, 2.7 Hz, 2H), 5.89 (hept, *J* = 6.2 Hz, 1H), 1.33 (s, 6H), 1.31 (s, 9H).

^{13}C{^{1}H} NMR (101 MHz, CDCl$_3$) δ 159.9, 152.2, 151.9, 137.0, 132.5, 129.9, 128.2, 127.4, 126.1, 126.1, 125.4, 121.5, 121.3, 120.54 (q, *J* = 282.6 Hz), 66.13 (p, *J* = 34.8 Hz), 55.0, 34.7, 31.1, 25.4.

^{19}F NMR (376 MHz, CDCl$_3$) δ -72.67 (d, *J* = 5.8 Hz).

HRMS (ESI-TOF) m/z: $[M+H]^+$ Calcd. for $C_{25}H_{25}F_6O_2S^+$ 503.1474; Found 503.1477.

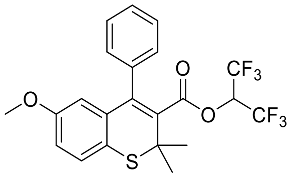

1,1,1,3,3,3-hexafluoropropan-2-yl 6-methoxy-2,2-dimethyl-4-phenyl-2*H*-thiochromene-3-carboxylate (4e)

Upon completion the mixture was concentrated and purified via flash column chromatography (petroleum ether : ethyl acetate = 100 : 1) to give the titled product **4e** as a **yellow oil** (50.5 mg, 53%, 96% purity).

^{1}H NMR (400 MHz, CDCl$_3$) δ 7.69 (d, J = 7.7 Hz, 1H), 7.51 (d, J = 8.7 Hz, 2H), 7.32 – 7.28 (m, 1H), 7.24 (d, J = 3.8 Hz, 2H), 6.88 (d, J = 8.7 Hz, 2H), 5.99 (hept, J = 6.2 Hz, 1H), 3.83 (s, 3H), 1.27 (s, 6H).

^{13}C{^{1}H} NMR (101 MHz, CDCl$_3$) δ 160.5, 160.3, 152.3, 136.8, 136.0, 134.6, 127.4, 125.9, 124.8, 124.1, 121.4, 121.1, 120.63 (q, J = 282.5 Hz), 114.5, 66.37 (p, J = 34.5 Hz), 55.4, 25.7.

^{19}F NMR (376 MHz, CDCl$_3$) δ -72.69 (s).

HRMS (ESI-TOF) m/z: [M+H]$^+$ Calcd. for $C_{22}H_{19}F_6O_3S^+$ 477.0954; Found 477.0957.

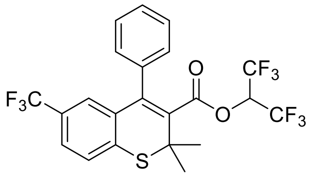

1,1,1,3,3,3-hexafluoropropan-2-yl 2,2-dimethyl-4-phenyl-6-(trifluoromethyl)-2*H*-thiochromene-3-carboxylate (4f)

Upon completion the mixture was concentrated and purified via flash column chromatography (petroleum ether : ethyl acetate = 100 : 1) to give the titled product **4f** as a **yellow oil** (50.4 mg, 49%, 96% purity).

^{1}H NMR (400 MHz, CDCl$_3$) δ 7.75 – 7.73 (m, 1H), 7.51 (d, J = 8.3 Hz, 2H), 7.43 (d, J = 8.4 Hz, 2H), 7.38 (dt, J = 5.7, 4.8 Hz, 3H), 5.86 (hept, J = 6.0 Hz, 1H), 1.40 (s, 6H).

^{13}C{^{1}H} NMR (101 MHz, CDCl$_3$) δ 163.0, 159.7, 152.3, 138.9, 136.7, 131.0, 129.7, 129.4, 129.0, 127.7, 127.2, 125.94 (d, J = 3.6 Hz), 123.83 (d, J = 272.2 Hz), 122.2, 121.8, 120.01 (d, J = 284.8 Hz), 118.95 (d, J = 2.6 Hz), 66.49 (p, J = 34.9 Hz), 54.7,

24.7.

^{19}F NMR (376 MHz, $CDCl_3$) δ -62.71 (s), -72.81 (d, J = 5.5 Hz).

HRMS (ESI-TOF) m/z: $[M+H]^+$ Calcd. for $C_{22}H_{16}F_9O_2S^+$ 515.0722; Found 515.0724.

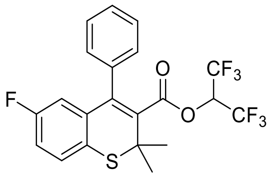

1,1,1,3,3,3-hexafluoropropan-2-yl 6-fluoro-2,2-dimethyl-4-phenyl-2*H*-thiochromene-3-carboxylate (4g)

Upon completion the mixture was concentrated and purified via flash column chromatography (petroleum ether : ethyl acetate = 100 : 1) to give the titled product **4g** as a **yellow solid** (43.7 mg, 47%, 96% purity).

^{1}H NMR (400 MHz, $CDCl_3$) δ 7.69 (d, J = 7.4 Hz, 1H), 7.48 (dd, J = 8.7, 5.2 Hz, 2H), 7.32 (dt, J = 11.0, 4.5 Hz, 3H), 7.03 (t, J = 8.6 Hz, 2H), 5.93 (hept, J = 6.2 Hz, 1H), 1.31 (s, 6H).

^{13}C{^{1}H} NMR (101 MHz, $CDCl_3$) δ 162.83 (d, J = 249.5 Hz), 160.0, 152.2, 136.7, 134.8, 134.7, 129.89 (d, J = 3.5 Hz), 127.5, 127.0, 126.7, 126.4, 121.7, 121.4, 120.50 (q, J = 285.5 Hz), 116.28 (d, J = 22.2 Hz), 66.41 (p, J = 34.7 Hz), 55.0, 25.4.

^{19}F NMR (376 MHz, $CDCl_3$) δ -73.28 (d, J = 6.0 Hz), -112.72 (s).

HRMS (ESI-TOF) m/z: $[M+H]^+$ Calcd. for $C_{21}H_{16}F_7O_2S^+$ 465.0754; Found 465.0755.

M.p. 49.1 – 49.7 °C

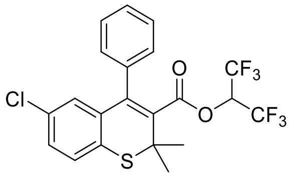

1,1,1,3,3,3-hexafluoropropan-2-yl 6-chloro-2,2-dimethyl-4-phenyl-2*H*-thiochromene-3-carboxylate (4h)

Upon completion the mixture was concentrated and purified via flash column chromatography (petroleum ether : ethyl acetate = 100 : 1) to give the titled product **4h** as a **yellow oil** (55.8 mg, 58%, 96% purity).

^{1}H NMR (400 MHz, $CDCl_3$) δ 7.70 (d, J = 6.8 Hz, 1H), 7.37 (d, J = 8.6 Hz, 2H), 7.34

– 7.30 (m, 3H), 7.29 (s, 2H), 5.92 (hept, J = 6.1 Hz, 1H), 1.34 (s, 6H).

$^{13}C\{^1H\}$ NMR (101 MHz, CDCl3) δ 159.9, 152.3, 136.7, 134.2, 132.8, 131.1, 129.3, 127.5, 126.7, 120.80 (q, J = 280.3 Hz), 121.8, 121.5, 66.09 (p, J = 34.9 Hz), 55.0, 25.2.

^{19}F NMR (376 MHz, CDCl3) δ -72.76 (d, J = 6.0 Hz).

HRMS (ESI-TOF) m/z: $[M+H]^+$ Calcd. for $C_{21}H_{16}ClF_6O_2S^+$ 481.0458; Found 481.0461.

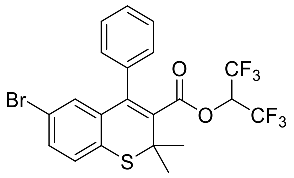

1,1,1,3,3,3-hexafluoropropan-2-yl 6-bromo-2,2-dimethyl-4-phenyl-2*H*-thiochromene-3-carboxylate (4i)

Upon completion the mixture was concentrated and purified via flash column chromatography (petroleum ether : ethyl acetate = 100 : 1) to give the titled product **4i** as a **yellow oil** (38.9 mg, 37%, 96% purity).

^1H NMR (400 MHz, CDCl3) δ 7.70 (d, J = 7.9 Hz, 1H), 7.42 (d, J = 8.4 Hz, 2H), 7.34 – 7.29 (m, 4H), 7.29 (s, 1H), 5.92 (hept, J = 6.1 Hz, 1H), 1.34 (s, 6H).

$^{13}C\{^1H\}$ NMR (101 MHz, CDCl3) δ 159.9, 152.3, 136.7, 132.9, 132.2, 131.8, 127.6, 126.7, 122.1, 121.9, 121.5, 121.41 (d, J = 281.4 Hz), 66.27 (p, J = 35.1 Hz), 55.0, 25.2.

^{19}F NMR (376 MHz, CDCl3) δ -72.79 (d, J = 5.7 Hz).

HRMS (ESI-TOF) m/z: $[M+H]^+$ Calcd. for $C_{21}H_{15}BrF_6O_2S^+$ 524.9953; Found 524.9956.

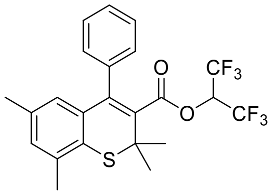

1,1,1,3,3,3-hexafluoropropan-2-yl 2,2,6,8-tetramethyl-4-phenyl-2*H*-thiochromene-3-carboxylate (4j)

Upon completion the mixture was concentrated and purified via flash column chromatography (petroleum ether : ethyl acetate = 100 : 1) to give the titled product **4j** as a **yellow oil** (71.2 mg, 75%, 96% purity).

^{1}H NMR (400 MHz, CDCl$_3$) δ 7.69 (d, J = 7.6 Hz, 1H), 7.39 (d, J = 7.9 Hz, 1H), 7.33 – 7.29 (m, 1H), 7.25 (d, J = 7.2 Hz, 2H), 7.06 (s, 1H), 6.96 (d, J = 7.9 Hz, 1H), 5.95 (hept, J = 6.2 Hz, 1H), 2.42 (s, 3H), 2.32 (s, 3H), 1.27 (s, 6H).

^{13}C{^{1}H} NMR (101 MHz, CDCl$_3$) δ 160.1, 152.3, 141.5, 140.3, 139.4, 137.0, 134.2, 131.4, 127.4, 126.8, 125.9, 125.0, 121.4, 121.1, 120.60 (q, J = 282.8 Hz), 66.32 (p, J = 34.6 Hz), 55.4, 25.3, 21.1, 20.9.

^{19}F NMR (376 MHz, CDCl$_3$) δ -72.76 (d, J = 5.9 Hz).

HRMS (ESI-TOF) m/z: [M+H]$^+$ Calcd. for $C_{23}H_{21}F_6O_2S^+$ 475.1161; Found 475.1163.

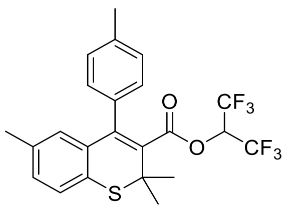

1,1,1,3,3,3-hexafluoropropan-2-yl 2,2,6-trimethyl-4-(*p*-tolyl)-2*H*-thiochromene-3-carboxylate (4k)

Upon completion the mixture was concentrated and purified via flash column chromatography (petroleum ether : ethyl acetate = 100 : 1) to give the titled product **4k** as a **yellow oil** (44.6 mg, 47%, 96% purity).

^{1}H NMR (400 MHz, CDCl$_3$) δ 7.57 (d, J = 7.9 Hz, 1H), 7.38 (d, J = 8.0 Hz, 2H), 7.12 (d, J = 7.7 Hz, 3H), 7.09 (s, 1H), 5.94 (hept, J = 6.1 Hz, 1H), 2.39 (s, 3H), 2.34 (s, 3H), 1.28 (s, 6H).

^{13}C{^{1}H} NMR (101 MHz, CDCl$_3$) δ 160.1, 152.6, 140.4, 138.6, 136.2, 134.3, 132.8, 129.8, 128.2, 122.1, 121.3, 120.87 (d, J = 286.0 Hz), 120.7, 66.33 (p, J = 36.3 Hz), 55.0, 25.5, 21.5, 21.2.

^{19}F NMR (376 MHz, CDCl$_3$) δ -72.77 (d, J = 5.5 Hz).

HRMS (ESI-TOF) m/z: [M+H]$^+$ Calcd. for $C_{23}H_{21}F_6O_2S^+$ 475.1161; Found 475.1164.

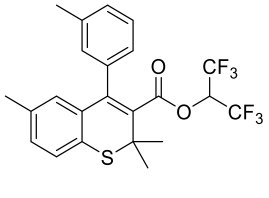

1,1,1,3,3,3-hexafluoropropan-2-yl 2,2,6-trimethyl-4-(*m*-tolyl)-2*H*-thiochromene-3-carboxylate (4l)

Upon completion the mixture was concentrated and purified via flash column chromatography (petroleum ether : ethyl acetate = 100 : 1) to give the titled product **4l** as a **yellow oil** (48.4 mg, 51%, 96% purity).

^{1}H NMR (400 MHz, CDCl$_3$) δ 7.52 (d, J = 5.7 Hz, 1H), 7.39 (d, J = 7.9 Hz, 1H), 7.28 (d, J = 8.0 Hz, 1H), 7.20 (dd, J = 23.1, 7.7 Hz, 1H), 7.18 – 7.04 (m, 3H), 5.94 (hept, J = 5.7 Hz, 1H), 2.45 (d, J = 43.7 Hz, 3H), 2.33 (d, J = 12.5 Hz, 3H), 1.38 (d, J = 78.9 Hz, 6H).

^{13}C{^{1}H} NMR (101 MHz, CDCl$_3$) δ 160.2, 149.26 (d, J = 63.4 Hz), 138.7, 137.70 (d, J = 6.0 Hz), 137.04 (d, J = 5.3 Hz), 132.9, 130.9, 129.8, 129.8, 129.2, 127.4, 126.9, 122.2, 121.0, 120.52 (q, J = 283.6 Hz), 119.2, 66.18 (p, J = 34.7 Hz), 55.20 (d, J = 68.8 Hz), 25.6, 21.84 (d, J = 46.8 Hz), 21.09 (d, J = 11.0 Hz), 19.0.

^{19}F NMR (376 MHz, CDCl$_3$) δ -72.73 (dd, J = 12.5, 6.1 Hz).

HRMS (ESI-TOF) m/z: $[M+H]^+$ Calcd. for $C_{23}H_{21}F_6O_2S^+$ 475.1161; Found 475.1161.

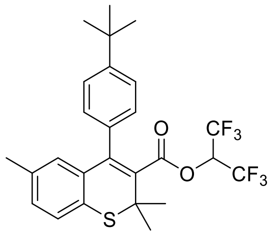

1,1,1,3,3,3-hexafluoropropan-2-yl 4-(4-(*tert*-butyl)phenyl)-2,2,6-trimethyl-2*H*-thiochromene-3-carboxylate (4m)

Upon completion the mixture was concentrated and purified via flash column chromatography (petroleum ether : ethyl acetate = 100 : 1) to give the titled product **4m** as a **yellow oil** (65.1 mg, 63%, 96% purity).

^{1}H NMR (400 MHz, CDCl$_3$) δ 7.62 (d, J = 8.2 Hz, 1H), 7.36 (d, J = 7.9 Hz, 3H), 7.30 (s, 1H), 7.11 (d, J = 7.9 Hz, 2H), 5.93 (hept, J = 6.2 Hz, 1H), 2.34 (s, 3H), 1.34 (s, 9H), 1.31 (s, 6H).

^{13}C{^{1}H} NMR (101 MHz, CDCl$_3$) δ 160.0, 152.2, 149.7, 138.5, 134.3, 132.5, 129.8, 129.5, 128.4, 124.6, 121.1, 120.55 (d, J = 279.3 Hz), 118.2, 66.30 (p, J = 34.6 Hz), 55.1, 34.8, 31.5, 25.6, 21.1.

^{19}F NMR (376 MHz, CDCl$_3$) δ -72.78 (d, J = 5.4 Hz).

HRMS (ESI-TOF) m/z: $[M+H]^+$ Calcd. for $C_{26}H_{27}F_6O_2S^+$ 517.1630; Found 517.1635.

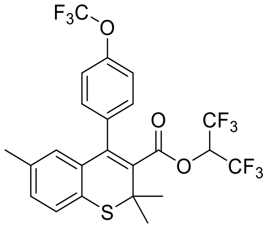

1,1,1,3,3,3-hexafluoropropan-2-yl 2,2,6-trimethyl-4-(4-(trifluoromethoxy)phenyl)-2*H*-thiochromene-3-carboxylate (4n)

Upon completion the mixture was concentrated and purified via flash column chromatography (petroleum ether : ethyl acetate = 100 : 1) to give the titled product **4n** as a **yellow oil** (55.5 mg, 51%, 96% purity).

^{1}H NMR (400 MHz, CDCl$_3$) δ 7.68 (d, J = 8.5 Hz, 1H), 7.41 (d, J = 8.0 Hz, 2H), 7.16 (t, J = 9.5 Hz, 3H), 7.10 (s, 1H), 5.94 (hept, J = 6.1 Hz, 1H), 2.36 (s, 3H), 1.29 (s, 6H).

^{13}C{^{1}H} NMR (101 MHz, CDCl$_3$) δ 159.7, 154.0, 147.8, 139.3, 135.5, 133.6, 129.9, 127.1, 122.4, 122.51 (d, J = 270.9 Hz), 120.58 (d, J = 277.5 Hz), 120.2, 114.5, 66.07 (p, J = 35.0 Hz), 55.4, 25.5, 21.2.

^{19}F NMR (376 MHz, CDCl$_3$) δ -57.86 (s), -72.76 (d, J = 6.0 Hz).

HRMS (ESI-TOF) m/z: $[M+H]^+$ Calcd. for $C_{23}H_{18}F_9O_3S^+$ 545.0827; Found 545.0831.

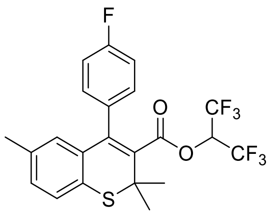

1,1,1,3,3,3-hexafluoropropan-2-yl 4-(4-fluorophenyl)-2,2,6-trimethyl-2*H*-thiochromene-3-carboxylate (4o)

Upon completion the mixture was concentrated and purified via flash column chromatography (petroleum ether : ethyl acetate = 100 : 1) to give the titled product **4o** as a **yellow solid** (44.0 mg, 46%, 96% purity).

^{1}H NMR (400 MHz, CDCl$_3$) δ 7.63 (dd, J = 8.5, 5.1 Hz, 1H), 7.40 (d, J = 8.0 Hz, 2H), 7.14 (d, J = 7.9 Hz, 2H), 7.03 – 6.97 (m, 2H), 5.95 (hept, J = 6.1 Hz, 1H), 2.35 (s, 3H), 1.28 (s, 6H).

$^{13}C\{^1H\}$ NMR (101 MHz, $CDCl_3$) δ 162.01 (d, J = 246.1 Hz), 160.8, 159.8, 154.58 (d, J = 7.5 Hz), 139.0, 133.2, 132.7, 129.8, 127.6, 124.9, 122.7, 122.6, 120.54 (q, J = 282.3 Hz), 114.34 (d, J = 22.6 Hz), 109.2, 108.9, 66.06 (p, J = 34.7 Hz), 55.2, 25.5, 21.2.

^{19}F NMR (376 MHz, $CDCl_3$) δ -72.70 (d, J = 5.9 Hz), -115.53 (d, J = 4.2 Hz).

HRMS (ESI-TOF) m/z: $[M+H]^+$ Calcd. for $C_{22}H_{18}F_7O_2S^+$ 479.0910; Found 479.0914.

M.p. 89.9 – 91.3 °C

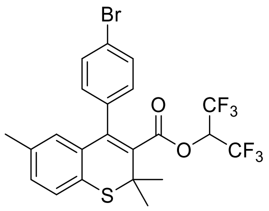

1,1,1,3,3,3-hexafluoropropan-2-yl 4-(4-bromophenyl)-2,2,6-trimethyl-2*H*-thiochromene-3-carboxylate (4p)

Upon completion the mixture was concentrated and purified via flash column chromatography (petroleum ether : ethyl acetate = 100 : 1) to give the titled product **4p** as a **yellow solid** (51.8 mg, 48%, 96% purity).

^1H NMR (400 MHz, $CDCl_3$) δ 7.54 (d, J = 8.3 Hz, 1H), 7.43 (dd, J = 11.9, 4.7 Hz, 3H), 7.38 (s, 1H), 7.15 (d, J = 7.9 Hz, 2H), 5.94 (hept, J = 6.1 Hz, 1H), 2.36 (s, 3H), 1.28 (s, 6H).

$^{13}C\{^1H\}$ NMR (101 MHz, $CDCl_3$) δ 160.0, 152.2, 151.9, 137.0, 132.5, 128.2, 127.4, 126.14, 126.10, 125.4, 121.9, 121.5, 121.3, 120.72 (d, J = 280.6 Hz), 66.14 (p, J = 34.7 Hz), 55.0, 34.7, 31.1, 25.4.

^{19}F NMR (376 MHz, $CDCl_3$) δ -72.78 (d, J = 5.9 Hz).

HRMS (ESI-TOF) m/z: $[M+H]^+$ Calcd. for $C_{22}H_{18}BrF_6O_2S^+$ 539.0110; Found 539.0113.

M.p. 99.0 – 99.6 °C

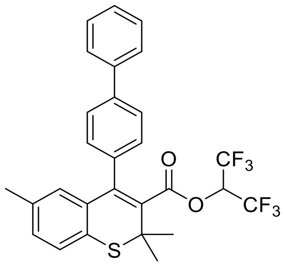

1,1,1,3,3,3-hexafluoropropan-2-yl 4-([1,1'-biphenyl]-4-yl)-2,2,6-trimethyl-2*H*-thiochromene-3-carboxylate (4q)

Upon completion the mixture was concentrated and purified via flash column chromatography (petroleum ether : ethyl acetate = 100 : 1) to give the titled product **4q** as a **yellow oil** (59.0 mg, 55%, 96% purity).

^{1}H NMR (400 MHz, $CDCl_3$) δ 7.75 (d, J = 8.1 Hz, 1H), 7.61 (d, J = 7.3 Hz, 2H), 7.56 (dd, J = 8.1, 1.6 Hz, 1H), 7.48 – 7.41 (m, 5H), 7.35 (t, J = 7.3 Hz, 1H), 7.15 (d, J = 8.0 Hz, 2H), 5.97 (hept, J = 6.1 Hz, 1H), 2.36 (s, 3H), 1.35 (s, 6H).

^{13}C{^{1}H} NMR (101 MHz, $CDCl_3$) δ 160.0, 152.9, 141.1, 139.3, 138.9, 136.1, 133.2, 129.8, 128.8, 127.8, 127.3, 127.2, 126.5, 125.1, 121.8, 120.56 (d, J = 278.8 Hz), 120.0, 66.04 (p, J = 34.9 Hz), 55.3, 25.6, 21.2.

^{19}F NMR (376 MHz, $CDCl_3$) δ -72.72 (d, J = 6.0 Hz).

HRMS (ESI-TOF) m/z: $[M+H]^+$ Calcd. for $C_{28}H_{23}F_6O_2S^+$ 537.1317; Found 537.1317.

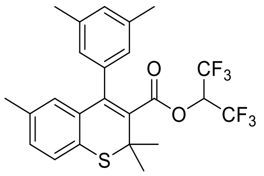

1,1,1,3,3,3-hexafluoropropan-2-yl 4-(3,5-dimethylphenyl)-2,2,6-trimethyl-2*H*-thiochromene-3-carboxylate (4r)

Upon completion the mixture was concentrated and purified via flash column chromatography (petroleum ether : ethyl acetate = 100 : 1) to give the titled product **4r** as a **yellow oil** (66.4 mg, 68%, 96% purity).

^{1}H NMR (400 MHz, $CDCl_3$) δ 7.34 (s, 1H), 7.27 (d, J = 6.4 Hz, 2H), 7.07 (d, J = 7.9 Hz, 2H), 6.88 (s, 1H), 5.85 (hept, J = 6.1 Hz, 1H), 2.46 (s, 3H), 2.35 (s, 3H), 2.31 (s, 3H), 1.45 (s, 6H).

$^{13}C\{^1H\}$ NMR (101 MHz, $CDCl_3$) *δ* 160.1, 146.2, 137.9, 137.6, 137.0, 132.5, 130.8, 130.0, 129.8, 129.7, 128.6, 120.48 (d, *J* = 280.1 Hz), 119.9, 66.16 (p, *J* = 35.1 Hz), 55.2, 22.2, 21.3, 21.0, 18.8.

^{19}F NMR (376 MHz, $CDCl_3$) *δ* -72.72 (d, *J* = 6.0 Hz).

HRMS (ESI-TOF) m/z: $[M+H]^+$ Calcd. for $C_{24}H_{23}F_6O_2S^+$ 489.1317; Found 489.1322.

4. Reference

[1]. Pammi, V. R.; Attunuri, N.; Jagadeesh, B. N.; Maddi, S. R. *Green Chem*. **2024**, *26*, 2705−2711.

[2]. Noelia, V.; Roberto, S.; Clara, M.-N.; Manuel, A.; Samuel, S.-P. *Adv. Synth. Catal.* **2022**, *364*, 2932−2938.

5. Copy of ^1H and $^{13}C\{^1H\}$ NMR Spectra of Products

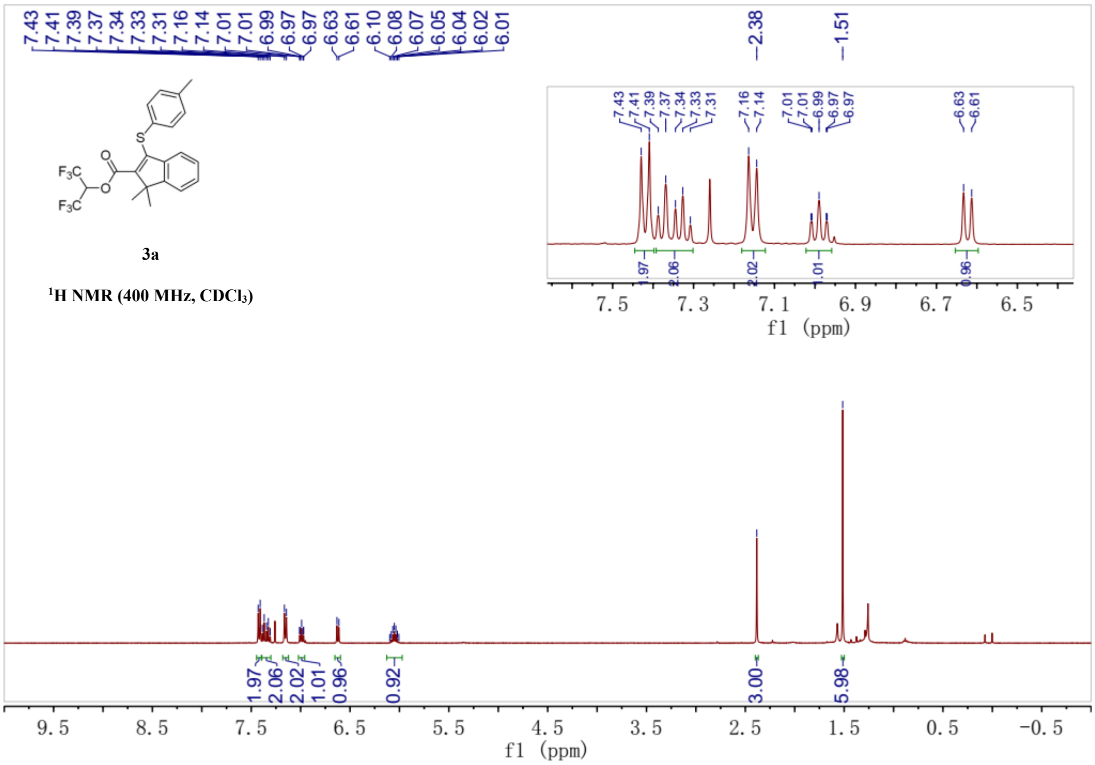

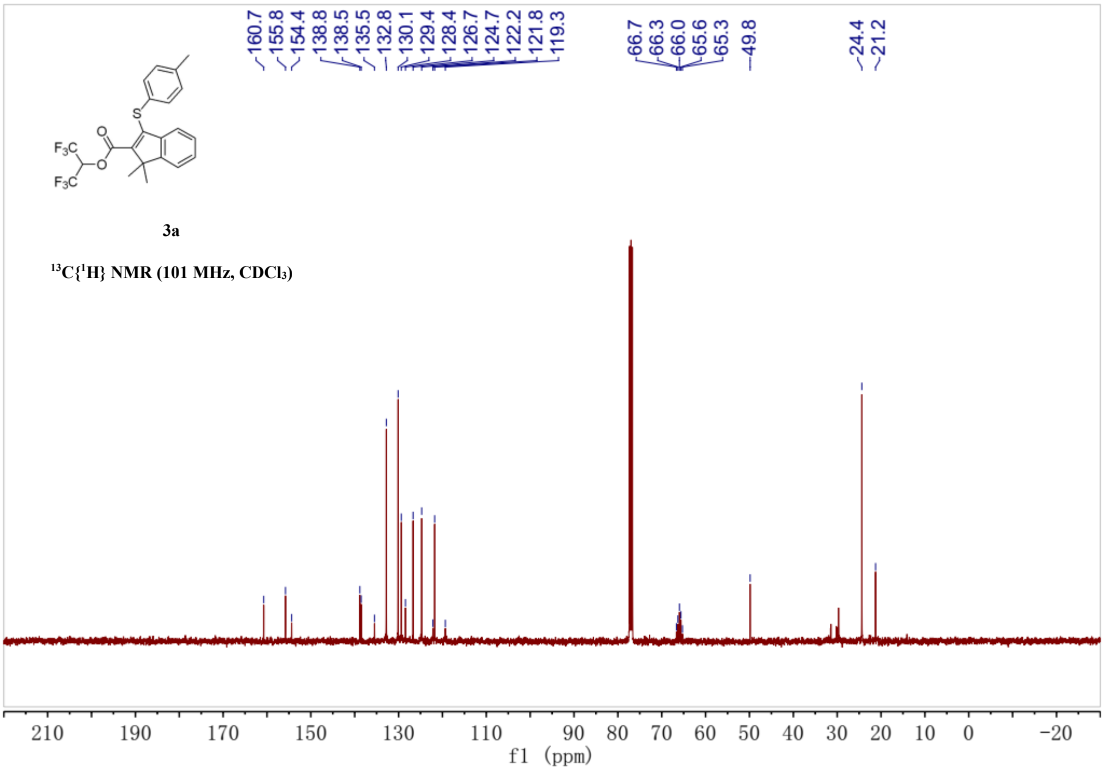

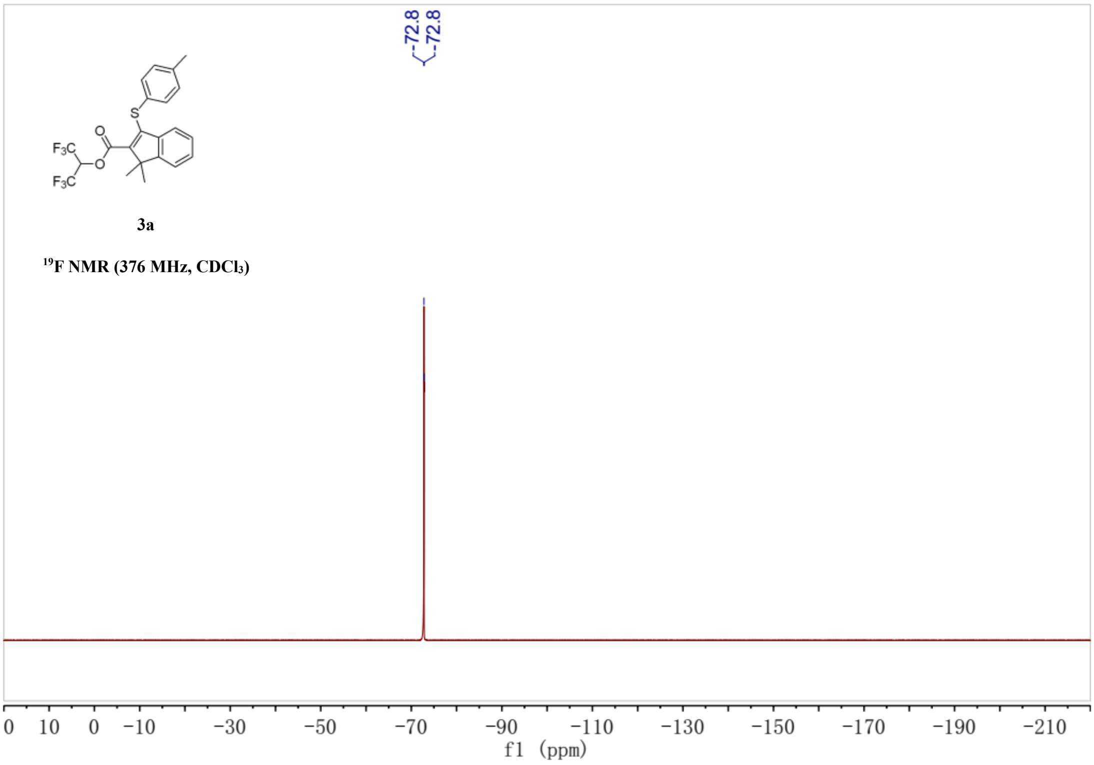
-72.8
-72.8
F3C
F3C
O
O
S
3a
19F NMR (376 MHz, CDCl3)
0 10 0 −10 −30 −50 −70 −90 −110 −130 −150 −170 −190 −210
f1 (ppm)

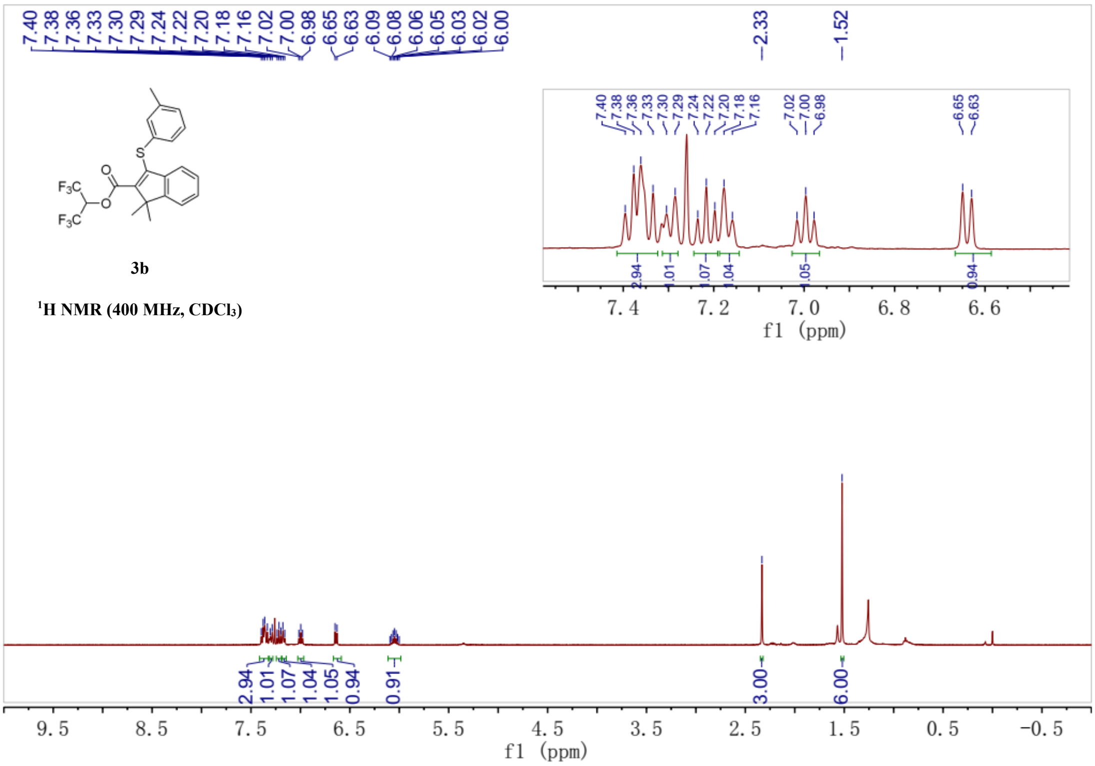
7.40 7.38 7.36 7.33 7.30 7.29 7.24 7.22 7.20 7.18 7.16 7.02 7.00 6.98 6.65 6.63 6.09 6.08 6.06 6.05 6.03 6.02 6.00
2.33
1.52
3b
1H NMR (400 MHz, CDCl3)
2.94 1.01 1.07 1.04 1.05 0.94 0.91 3.00 6.00
f1 (ppm)

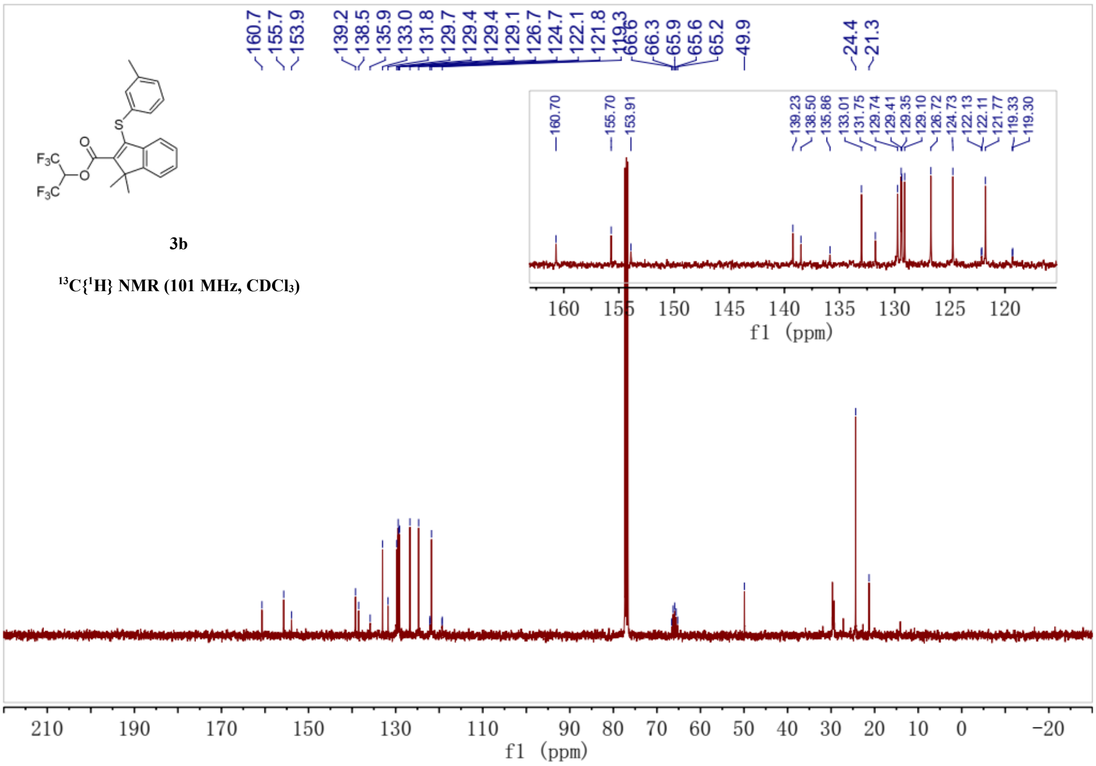
160.7 155.7 153.9 139.2 138.5 135.9 133.0 131.8 129.7 129.4 129.4 129.1 126.7 124.7 122.1 121.8 119.3 66.6 66.3 65.9 65.6 65.2 49.9 24.4 21.3
160.70 155.70 153.91 139.23 138.50 135.86 133.01 131.75 129.74 129.41 129.35 129.10 126.72 124.73 122.13 122.11 121.77 119.33 119.30
3b
13C{1H} NMR (101 MHz, CDCl3)
f1 (ppm)

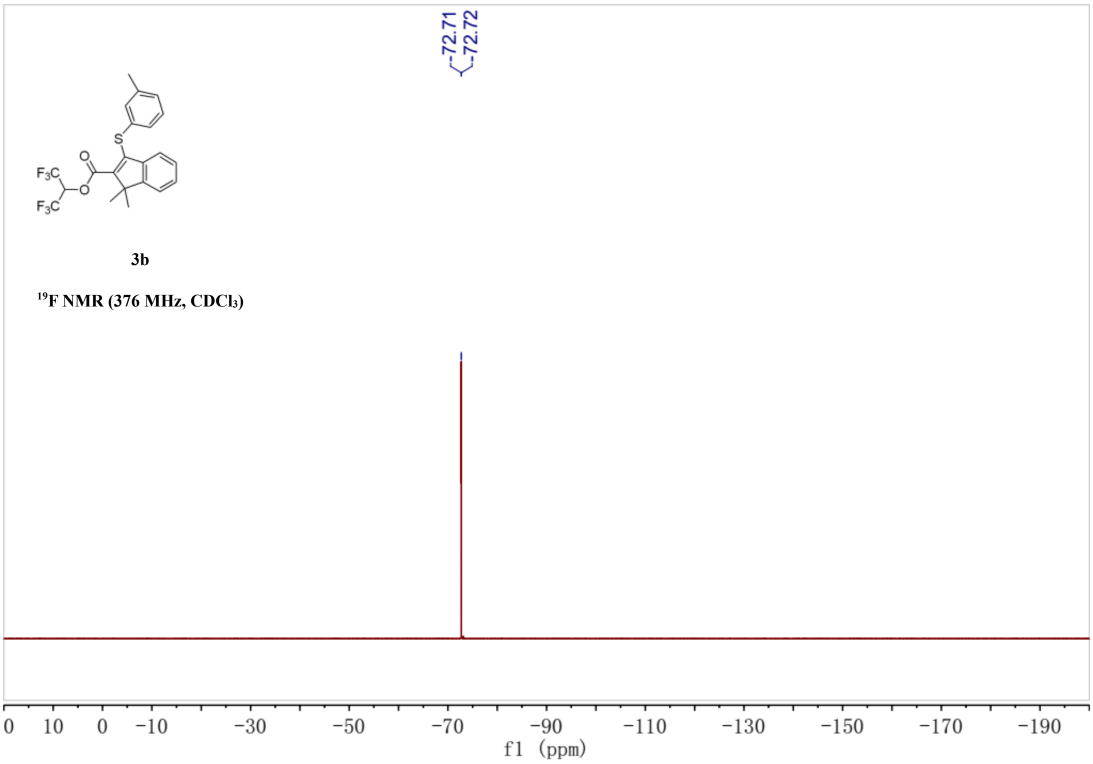

-72.71
-72.72
F_3C
F_3C
O
O
S
3b
^{19}F NMR (376 MHz, CDCl$_3$)
0 10 0 −10 −30 −50 −70 −90 −110 −130 −150 −170 −190
f1 (ppm)

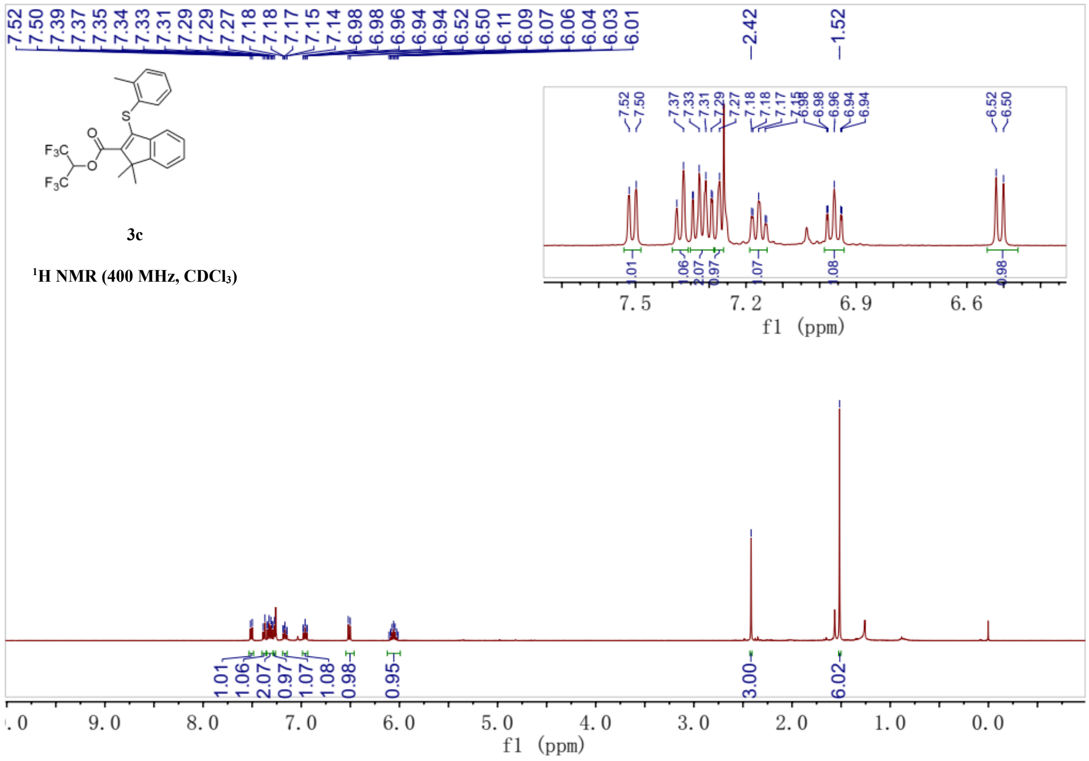
3c
1H NMR (400 MHz, CDCl3)
7.52 7.50 7.39 7.37 7.35 7.34 7.33 7.31 7.29 7.29 7.27 7.18 7.18 7.17 7.15 7.14 6.98 6.98 6.96 6.94 6.94 6.52 6.50 6.11 6.09 6.07 6.06 6.04 6.03 6.01
2.42
1.52
1.01 1.06 2.07 0.97 1.07 1.08 0.98 0.95 3.00 6.02
f1 (ppm)

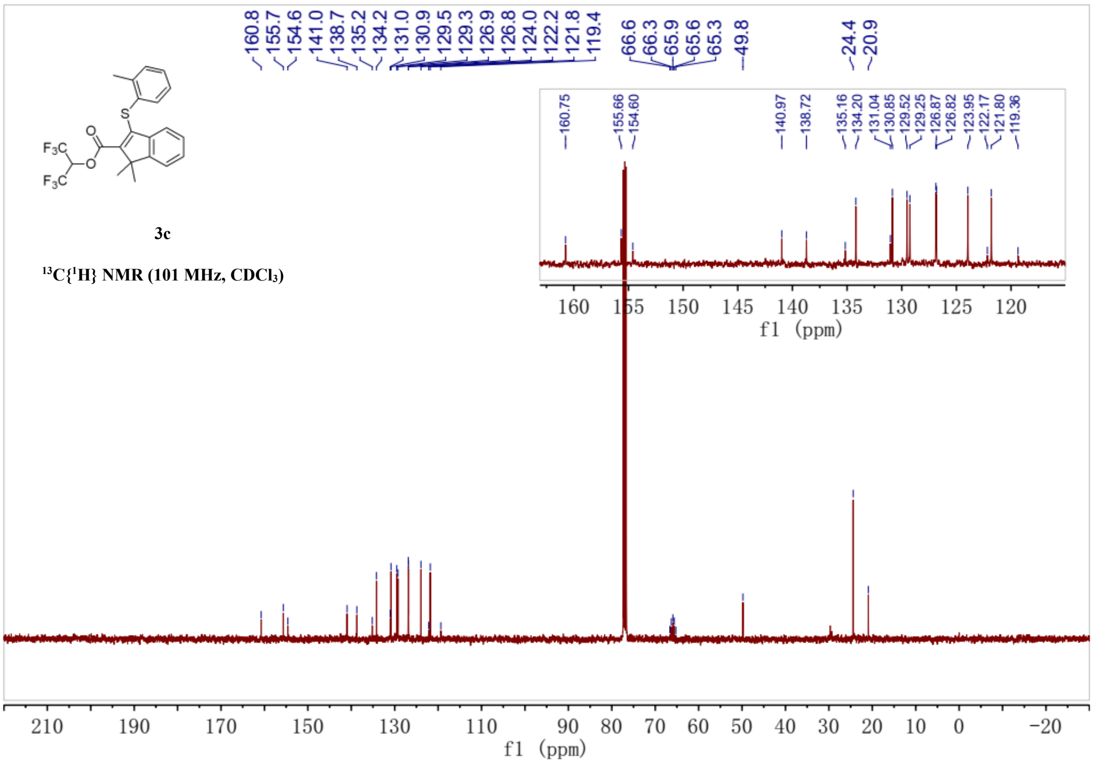
3c
13C{1H} NMR (101 MHz, CDCl3)
160.8 155.7 154.6 141.0 138.7 135.2 134.2 131.0 130.9 129.5 129.3 126.9 126.8 124.0 122.2 121.8 119.4
66.6 66.3 65.9 65.6 65.3 49.8
24.4 20.9
f1 (ppm)

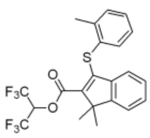

3c

^{19}F NMR (376 MHz, $CDCl_3$)

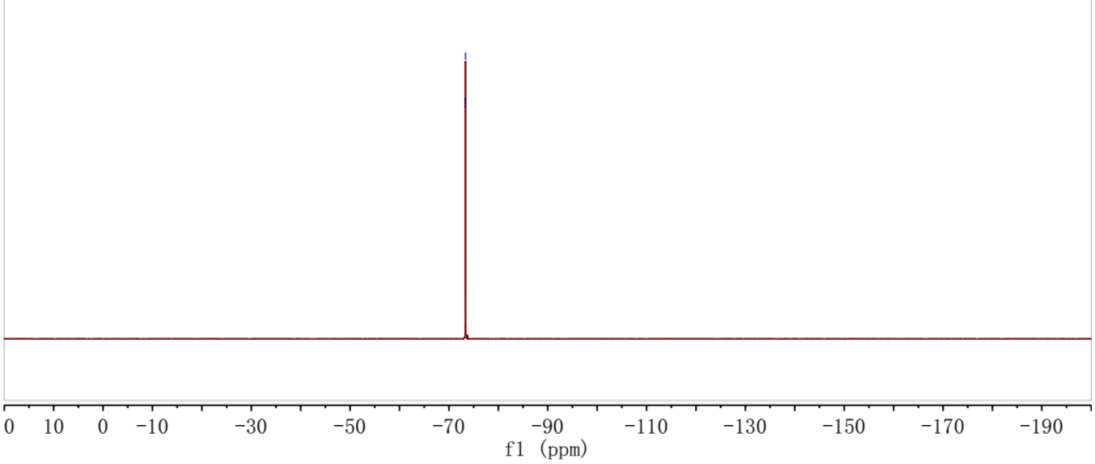

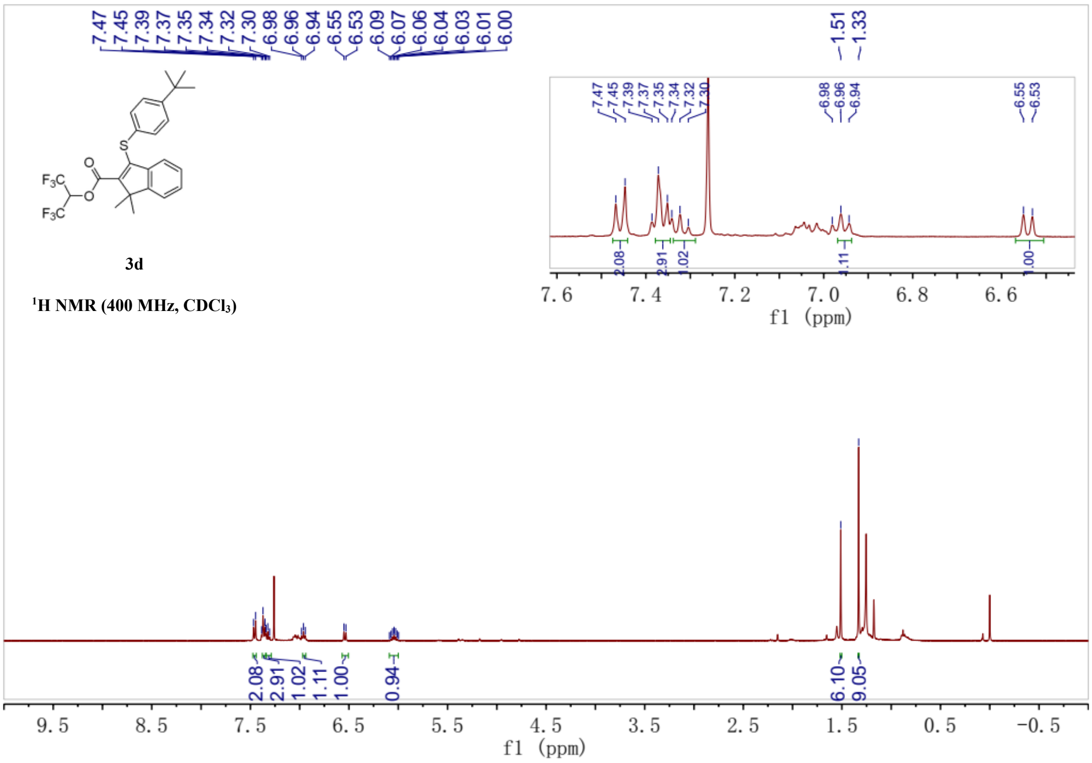
7.47 7.45 7.39 7.37 7.35 7.34 7.32 7.30 6.98 6.96 6.94 6.55 6.53 6.09 6.07 6.06 6.04 6.03 6.01 6.00
1.51 1.33
3d
^{1}H NMR (400 MHz, CDCl$_3$)
2.08 2.91 1.02 1.11 1.00 0.94 6.10 9.05
f1 (ppm)

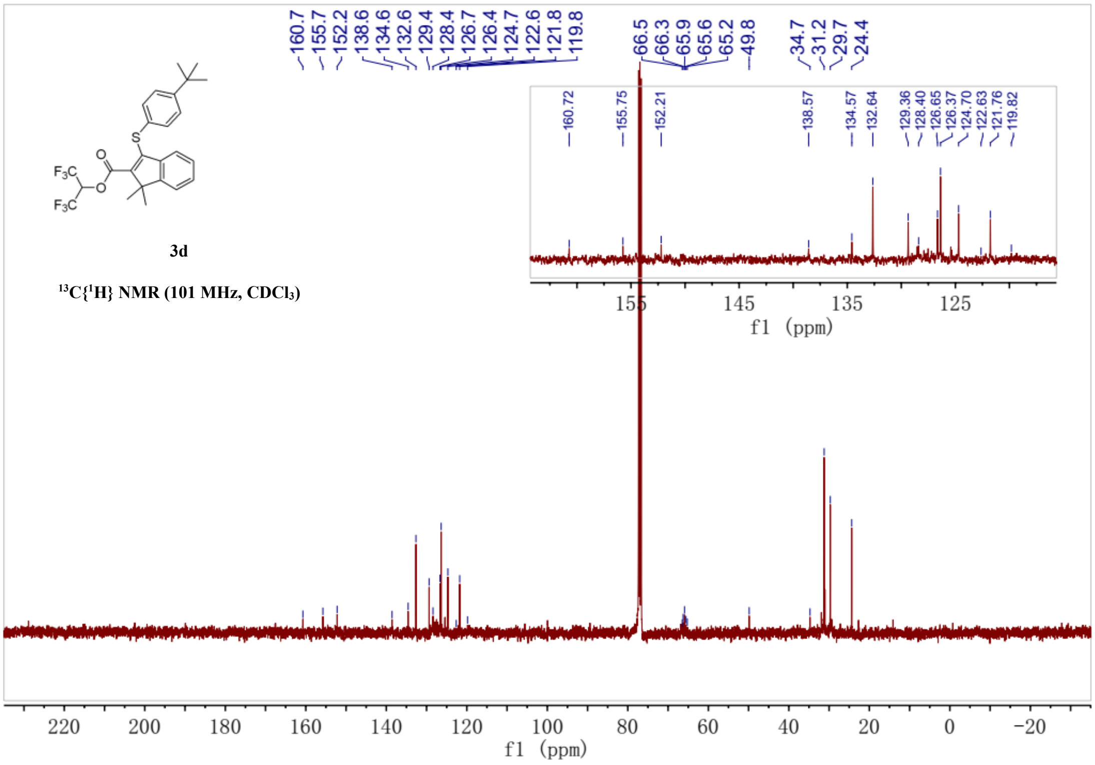
160.7 155.7 152.2 138.6 134.6 132.6 129.4 128.4 126.7 126.4 124.7 122.6 121.8 119.8
66.5 66.3 65.9 65.6 65.2 49.8
34.7 31.2 29.7 24.4
160.72 155.75 152.21 138.57 134.57 132.64 129.36 128.40 126.65 126.37 124.70 122.63 121.76 119.82
3d
^{13}C{^{1}H} NMR (101 MHz, CDCl$_3$)
f1 (ppm)

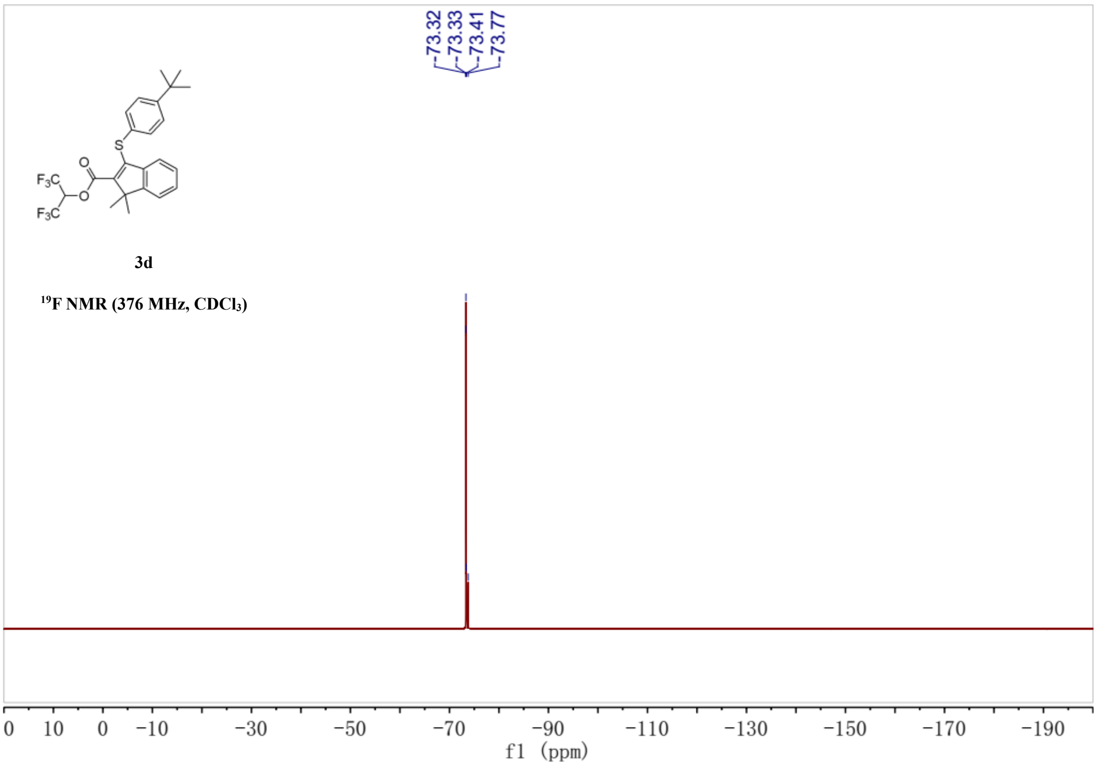
-73.32
-73.33
-73.41
-73.77
F3C
F3C
O
O
S
3d
19F NMR (376 MHz, CDCl3)
0
10
0
-10
-30
-50
-70
-90
-110
-130
-150
-170
-190
f1 (ppm)

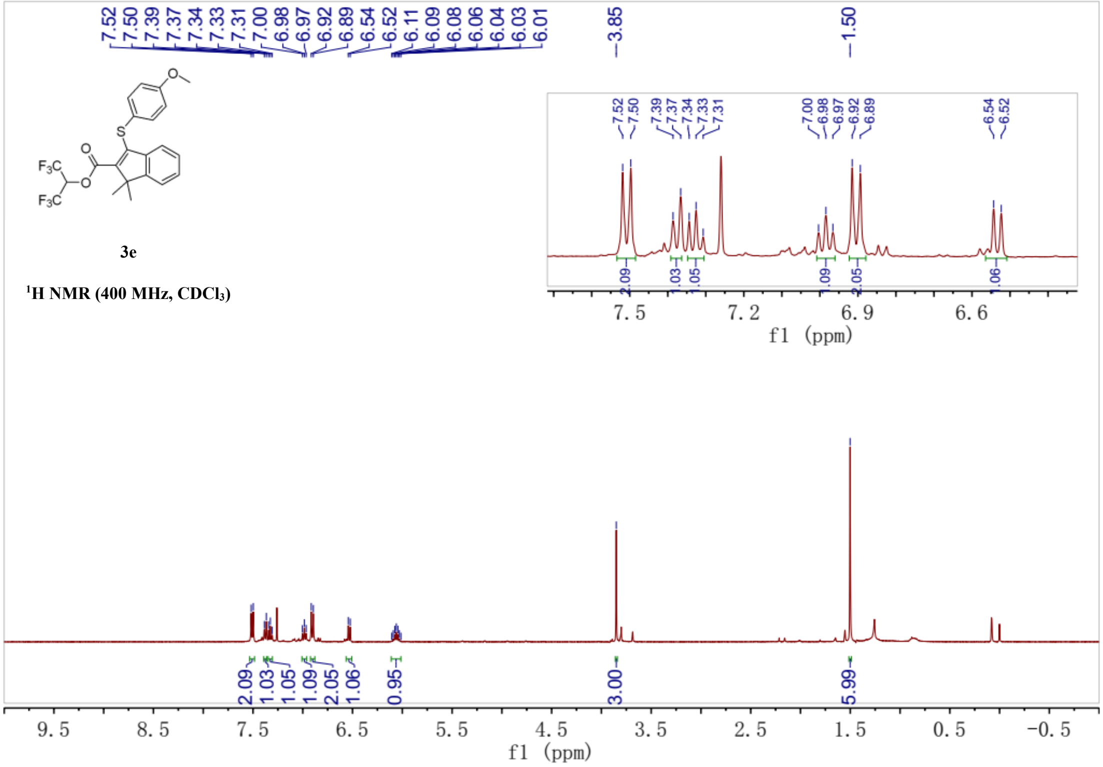
7.52
7.50
7.39
7.37
7.34
7.33
7.31
7.00
6.98
6.97
6.92
6.89
6.54
6.52
6.11
6.09
6.08
6.06
6.04
6.03
6.01
3.85
1.50
3e
^{1}H NMR (400 MHz, $CDCl_3$)
2.09
1.03
1.05
1.09
2.05
1.06
0.95
3.00
5.99
f1 (ppm)

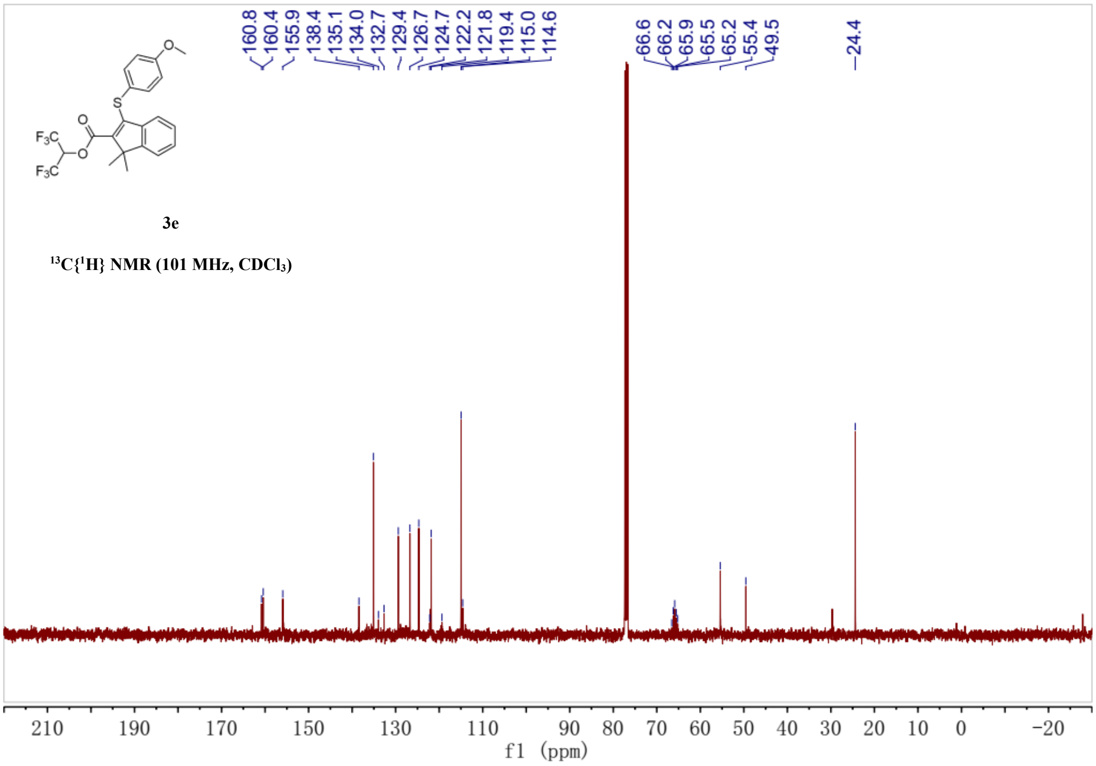
160.8
160.4
155.9
138.4
135.1
134.0
132.7
129.4
126.7
124.7
122.2
121.8
119.4
115.0
114.6
66.6
66.2
65.9
65.5
65.2
55.4
49.5
24.4
3e
$^{13}C\{^{1}H\}$ NMR (101 MHz, $CDCl_3$)
f1 (ppm)

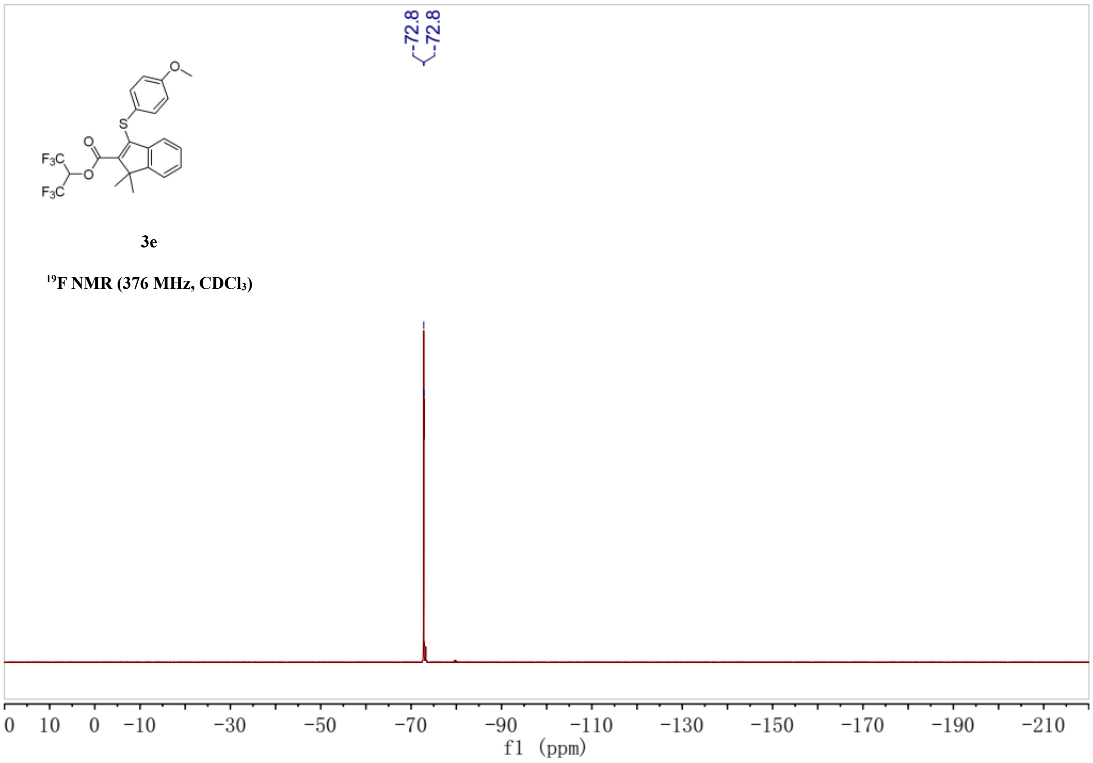
-72.8
-72.8
F3C
F3C
O
O
S
O
3e
19F NMR (376 MHz, CDCl3)
0 10 0 −10 −30 −50 −70 −90 −110 −130 −150 −170 −190 −210
f1 (ppm)

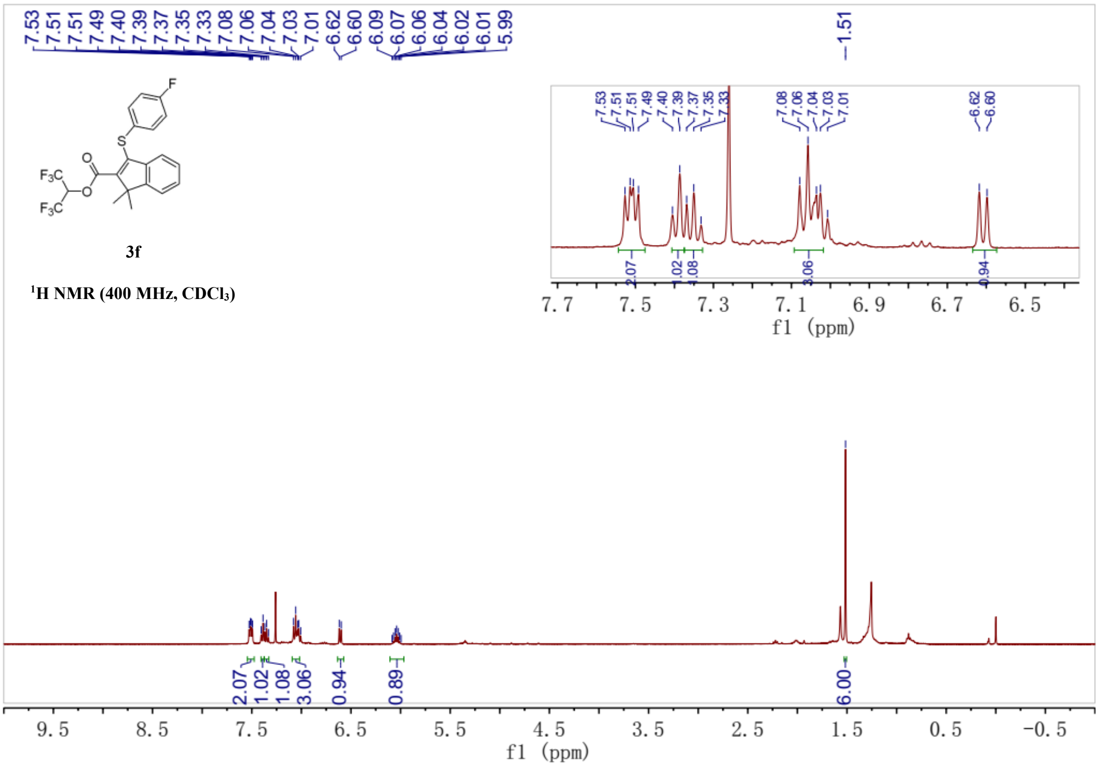

3f

^{1}H NMR (400 MHz, CDCl$_3$)

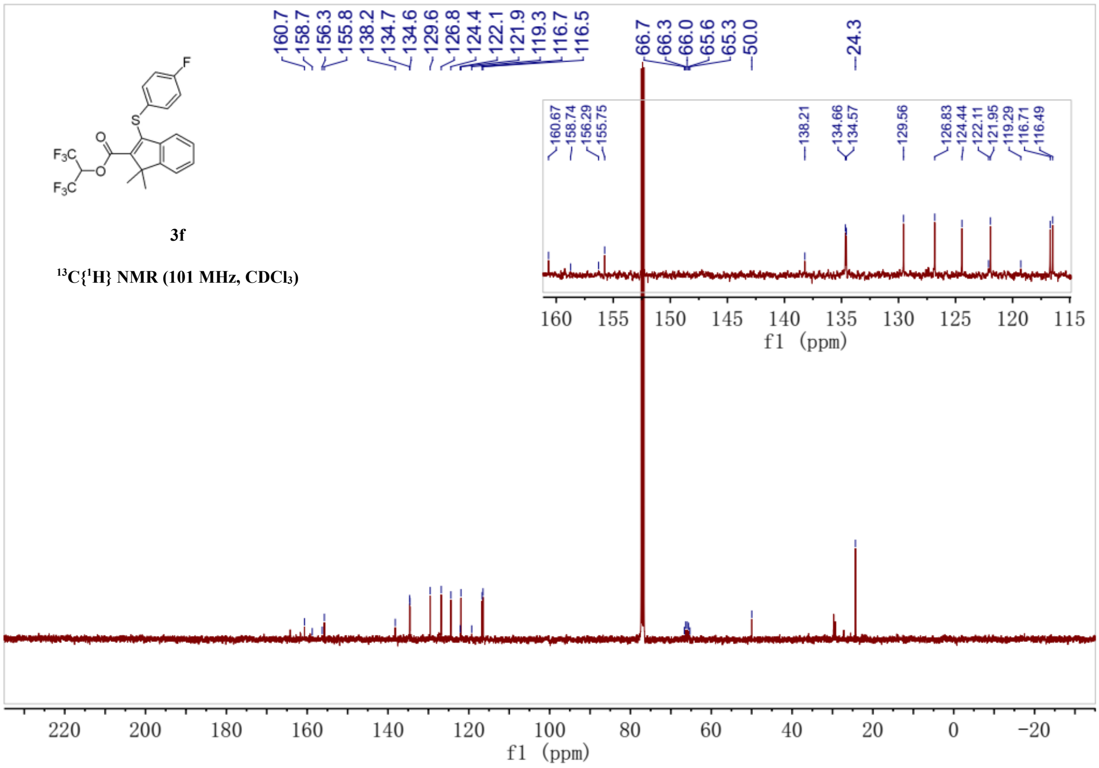

3f

^{13}C{^{1}H} NMR (101 MHz, CDCl$_3$)

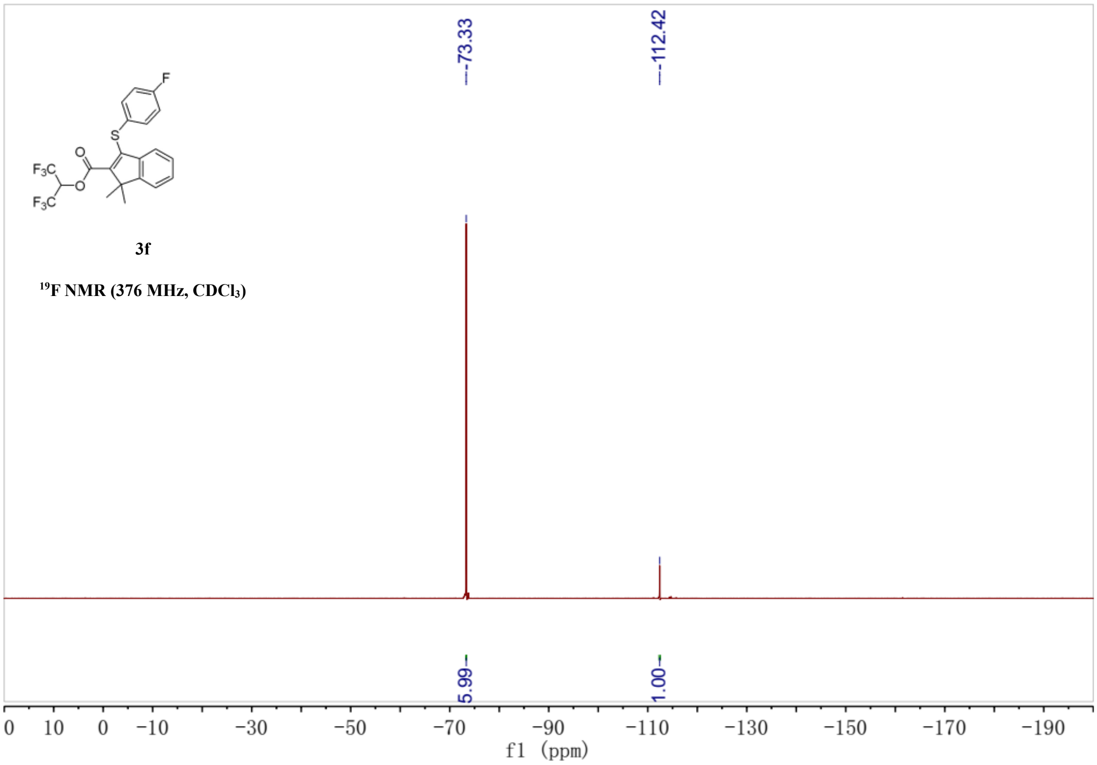

-73.33
-112.42
F
S
O
F3C
O
F3C
3f
19F NMR (376 MHz, CDCl3)
5.99
1.00
0
10
0
−10
−30
−50
−70
−90
−110
−130
−150
−170
−190
f1 (ppm)

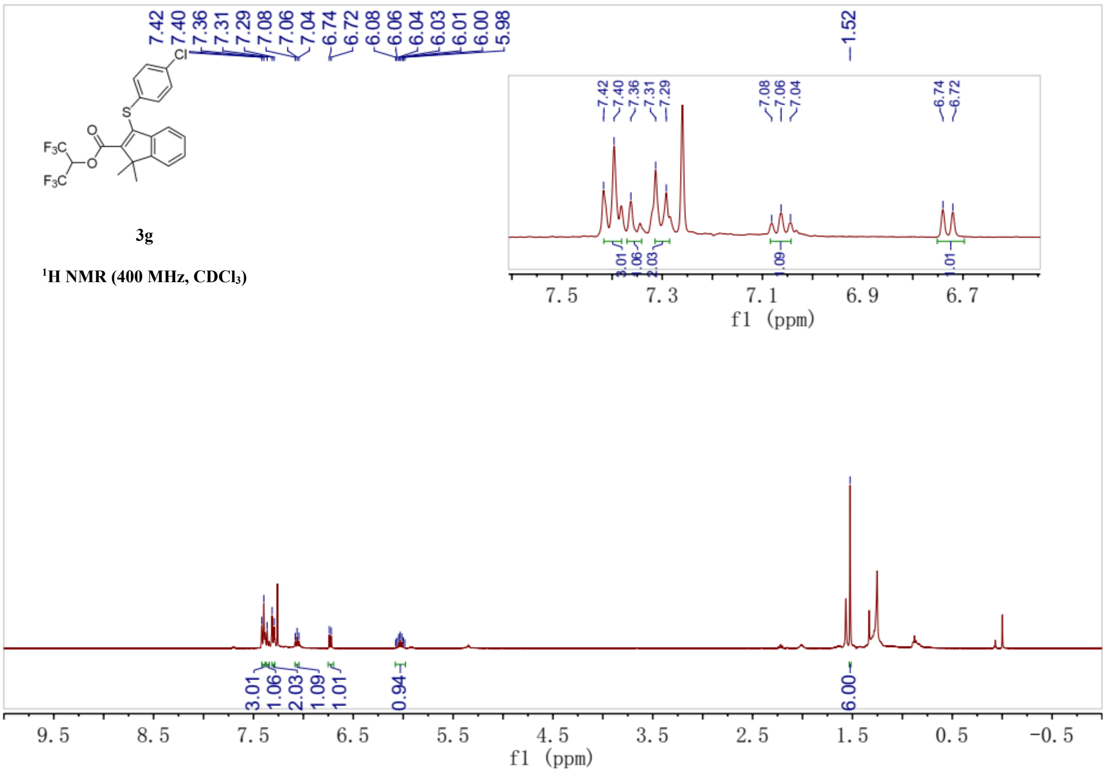
7.42 7.40 7.36 7.31 7.29 7.08 7.06 7.04 6.74 6.72 6.08 6.06 6.04 6.03 6.01 6.00 5.98
1.52
3g
^{1}H NMR (400 MHz, CDCl$_3$)
3.01 1.06 2.03 1.09 1.01 0.94 6.00
f1 (ppm)

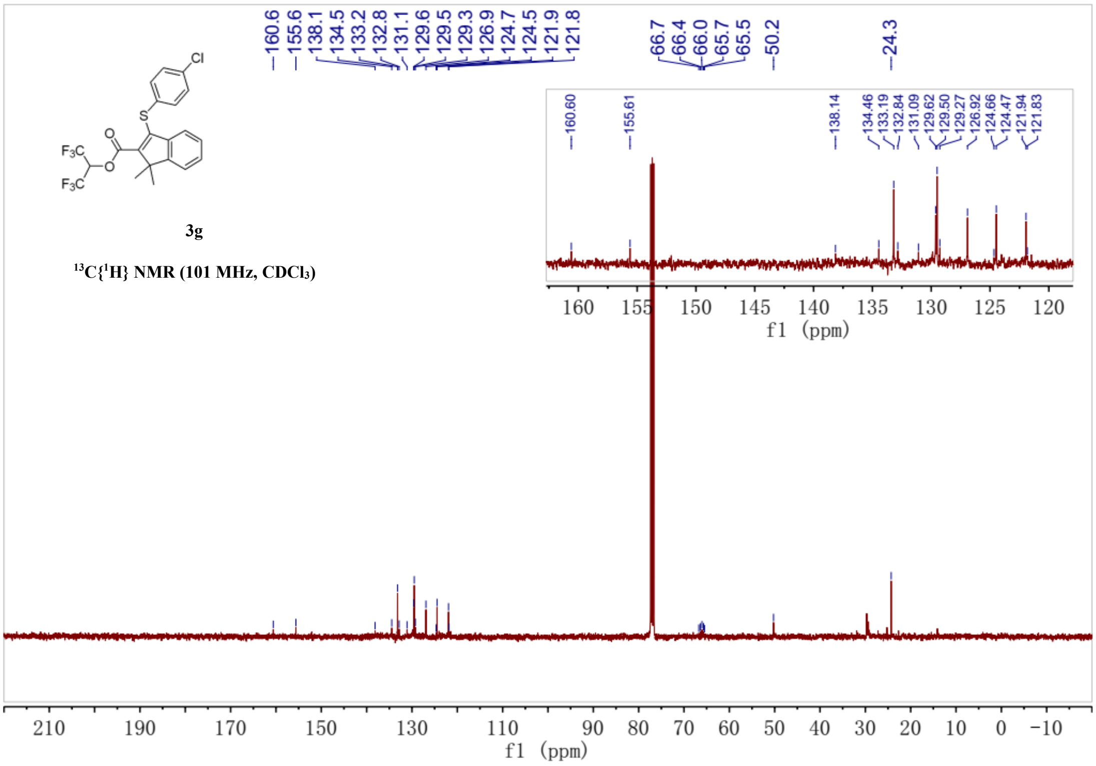
160.6 155.6 138.1 134.5 133.2 132.8 131.1 129.6 129.5 129.3 126.9 124.7 124.5 121.9 121.8
66.7 66.4 66.0 65.7 65.5 50.2 24.3
160.60 155.61 138.14 134.46 133.19 132.84 131.09 129.62 129.50 129.27 126.92 124.66 124.47 121.94 121.83
3g
^{13}C{^{1}H} NMR (101 MHz, CDCl$_3$)
f1 (ppm)

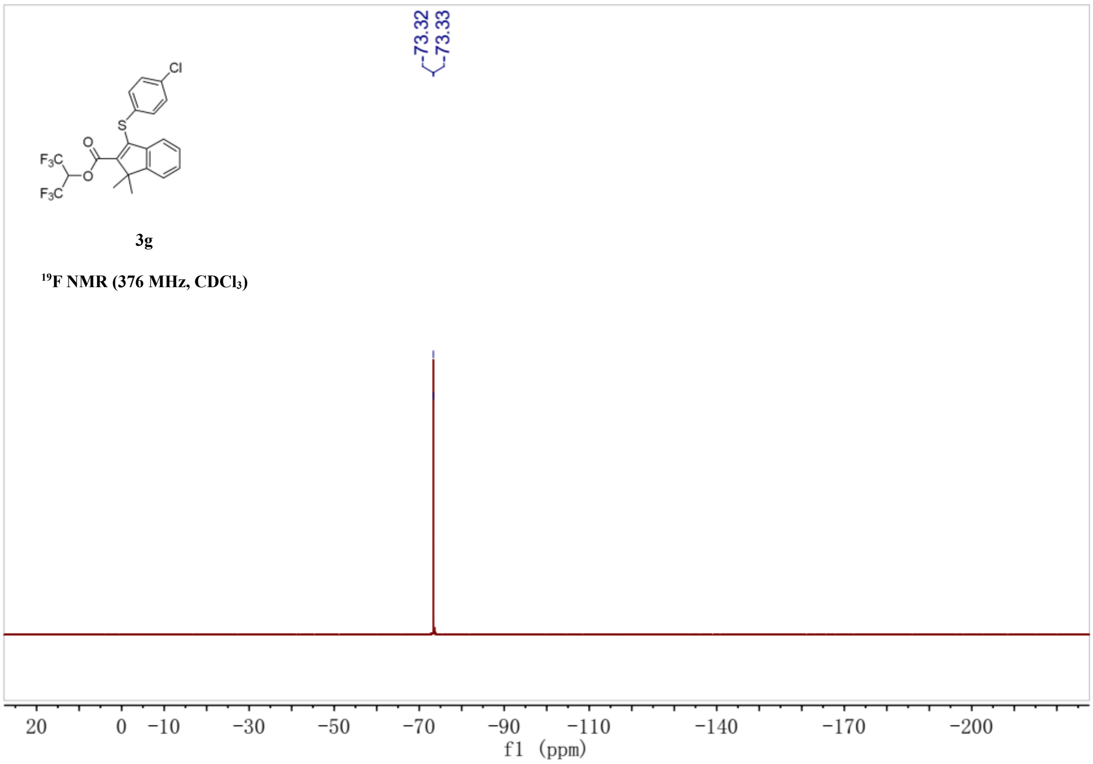
-73.32
-73.33
Cl
S
O
F3C
O
F3C
3g
19F NMR (376 MHz, CDCl3)
20
0
−10
−30
−50
−70
−90
−110
−140
−170
−200
f1 (ppm)

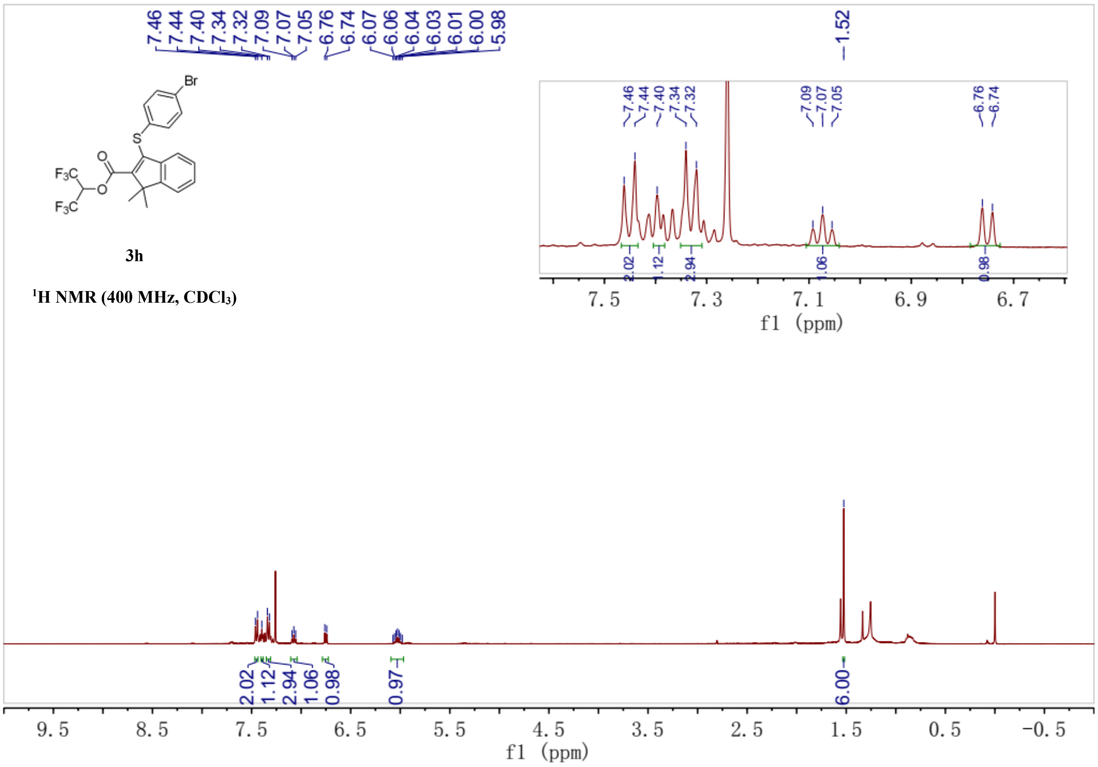

3h

^{1}H NMR (400 MHz, CDCl$_3$)

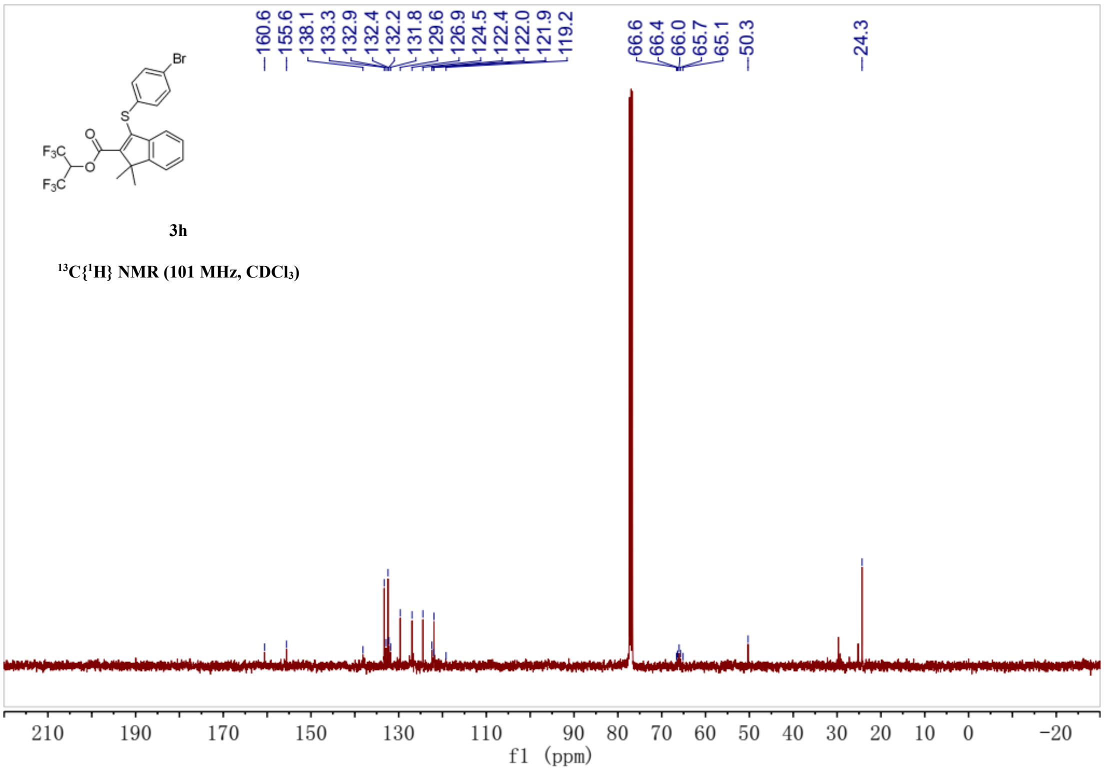

3h

^{13}C{^{1}H} NMR (101 MHz, CDCl$_3$)

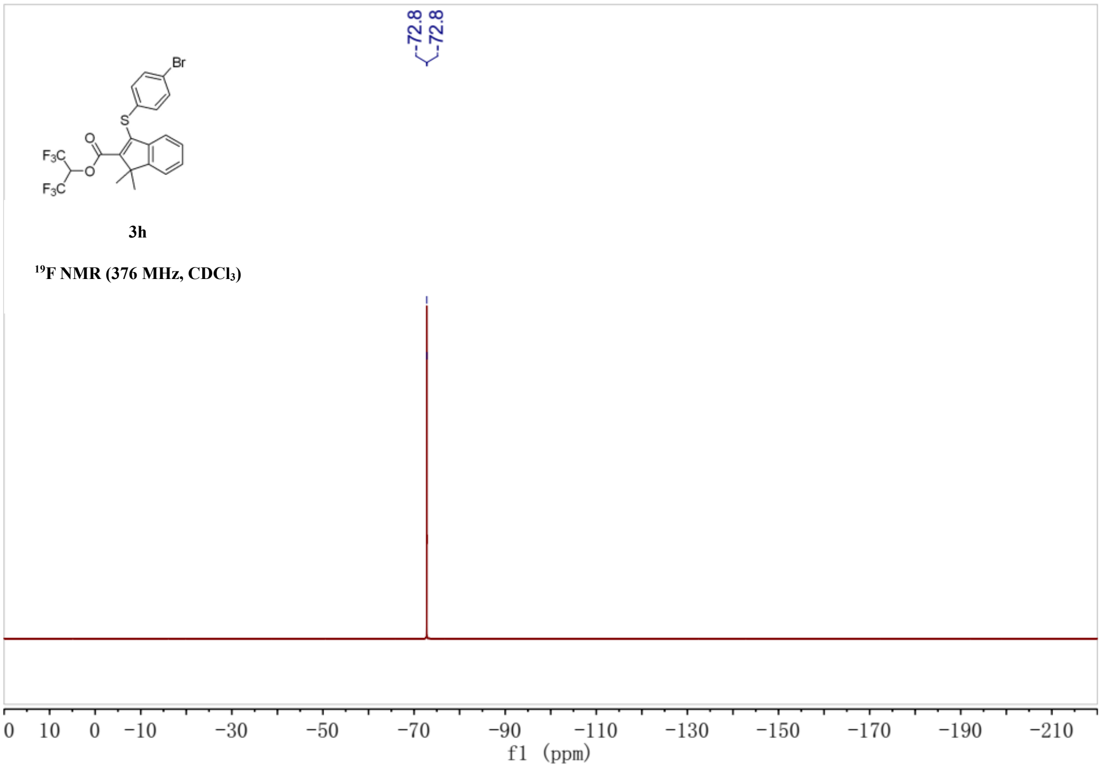
-72.8
-72.8
Br
S
O
F3C
O
F3C
3h
19F NMR (376 MHz, CDCl3)
0 10 0 −10 −30 −50 −70 −90 −110 −130 −150 −170 −190 −210
f1 (ppm)

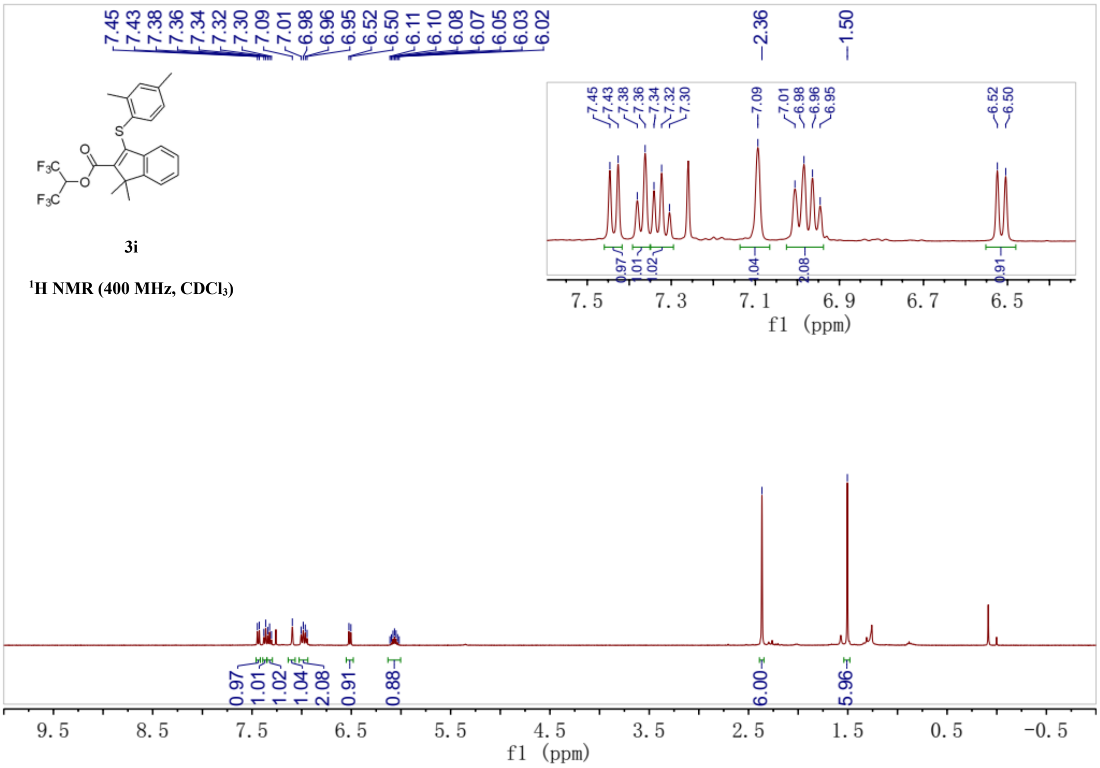

3i
1H NMR (400 MHz, CDCl3)
f1 (ppm)

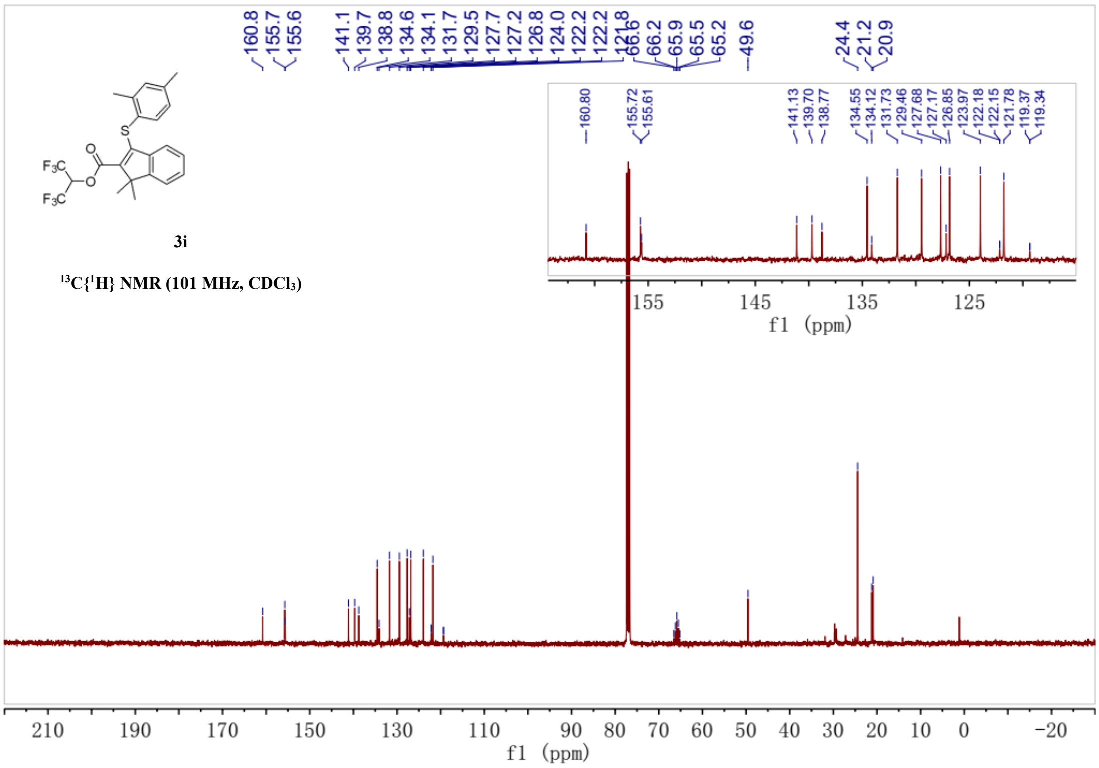

3i
13C{1H} NMR (101 MHz, CDCl3)
f1 (ppm)

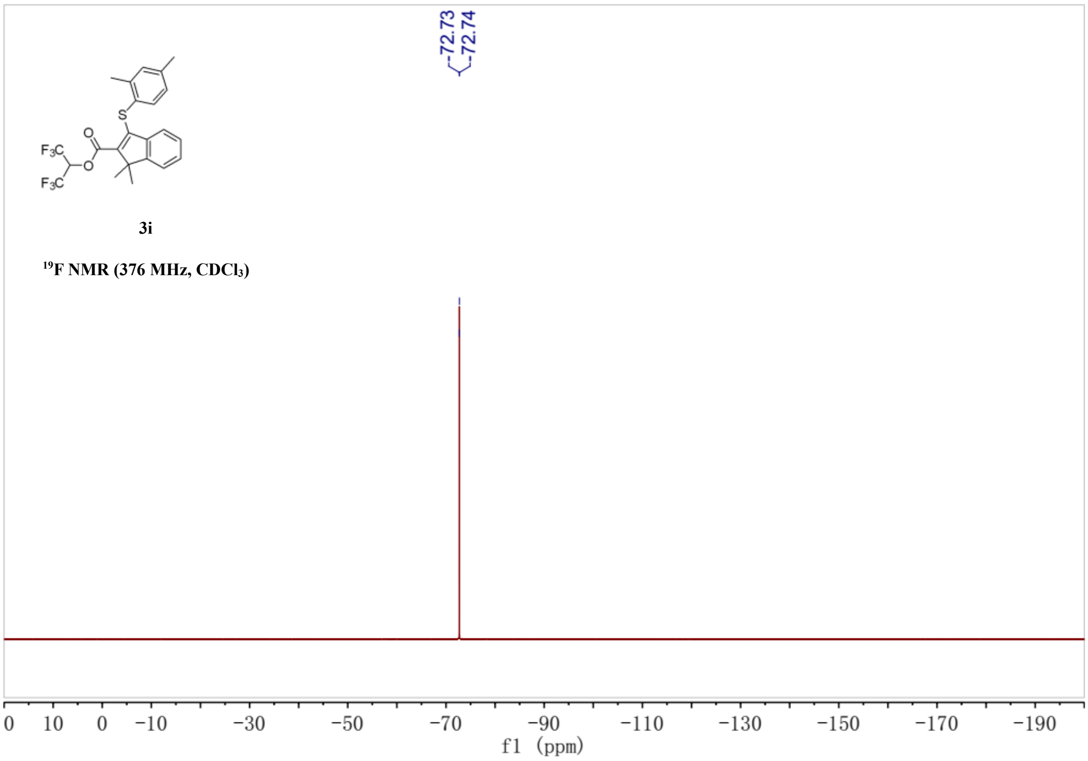
-72.73
-72.74
3i
19F NMR (376 MHz, CDCl3)
0 10 0 −10 −30 −50 −70 −90 −110 −130 −150 −170 −190
f1 (ppm)

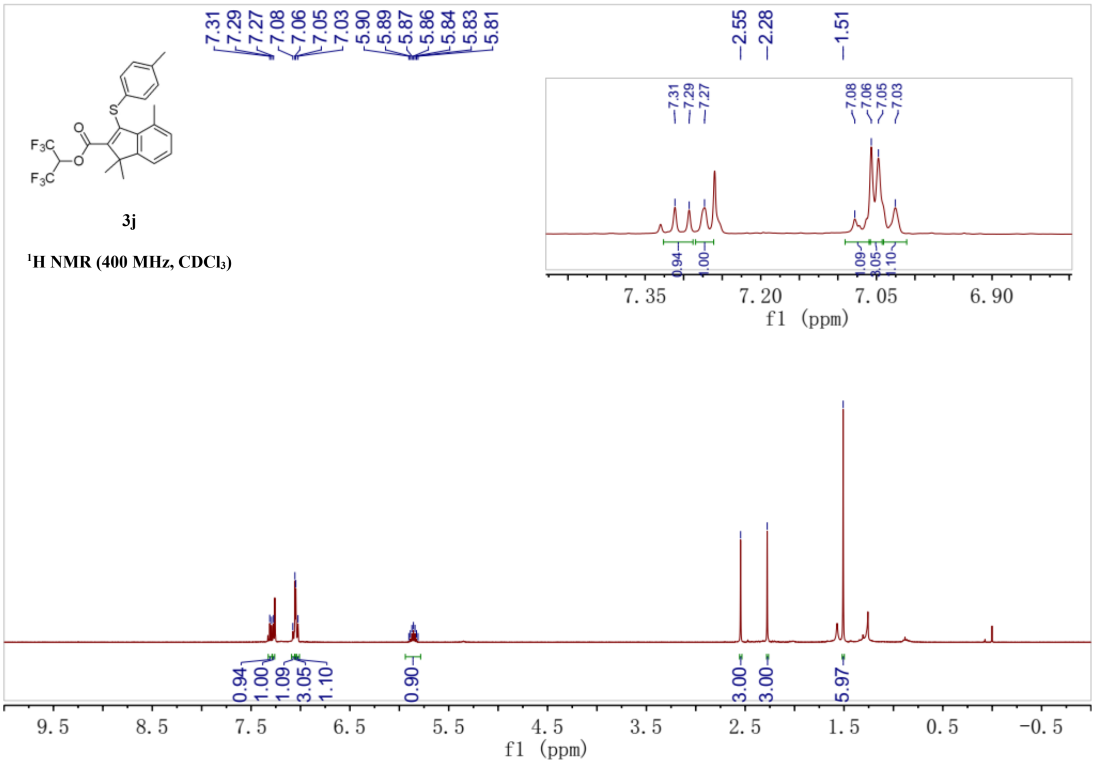
3j
1H NMR (400 MHz, CDCl3)
7.31 7.29 7.27 7.08 7.06 7.05 7.03 5.90 5.89 5.87 5.86 5.84 5.83 5.81
2.55 2.28 1.51
0.94 1.00 1.09 3.05 1.10 0.90 3.00 3.00 5.97
f1 (ppm)

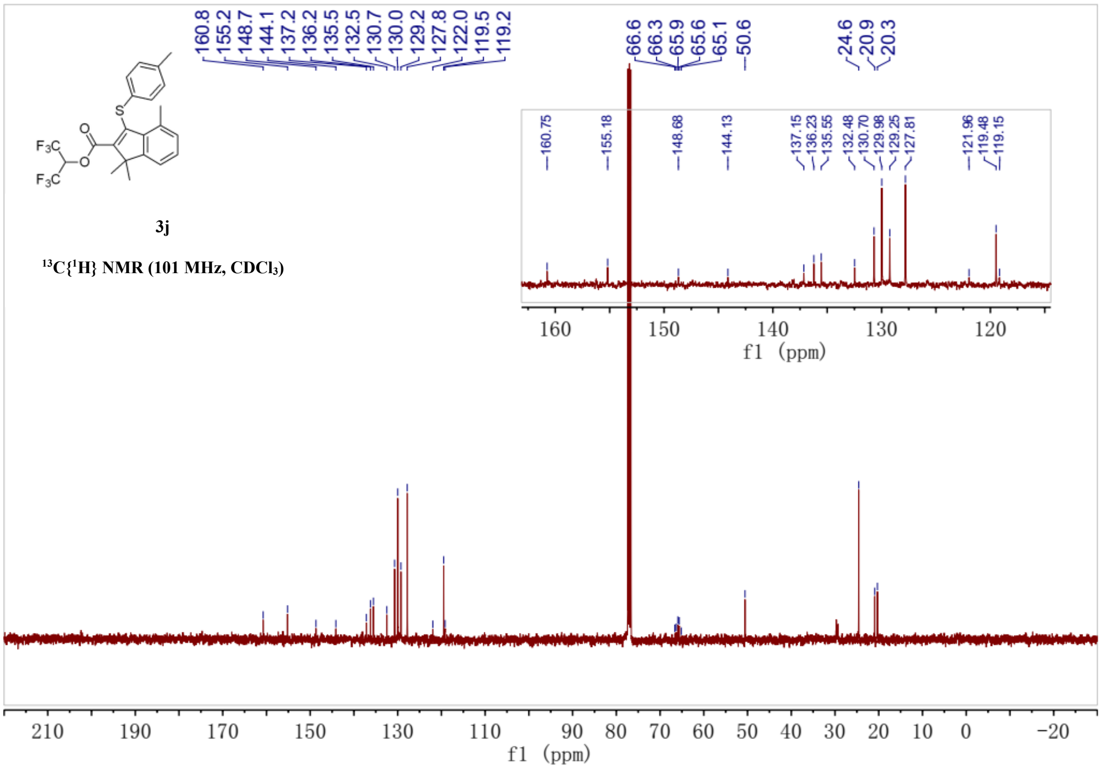
3j
13C{1H} NMR (101 MHz, CDCl3)
160.8 155.2 148.7 144.1 137.2 136.2 135.5 132.5 130.7 130.0 129.2 127.8 122.0 119.5 119.2
66.6 66.3 65.9 65.6 65.1 50.6 24.6 20.9 20.3
160.75 155.18 148.68 144.13 137.15 136.23 135.55 132.48 130.70 129.98 129.25 127.81 121.96 119.48 119.15
f1 (ppm)

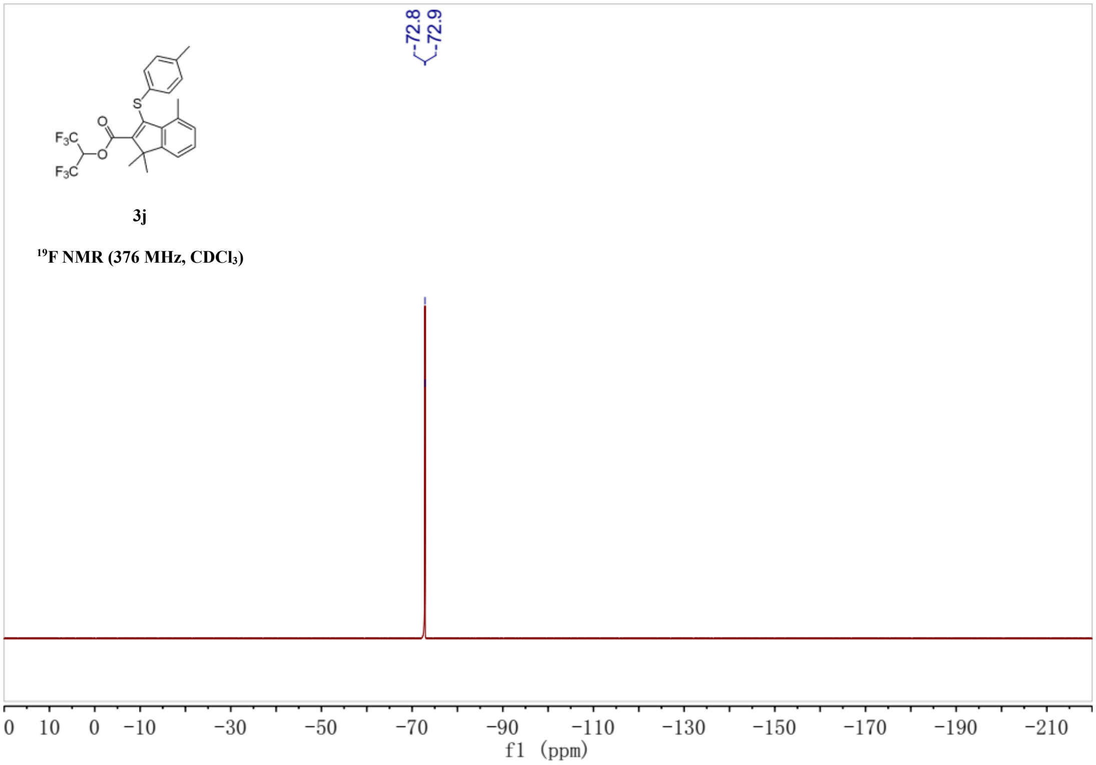

3j

^{19}F NMR (376 MHz, CDCl$_3$)

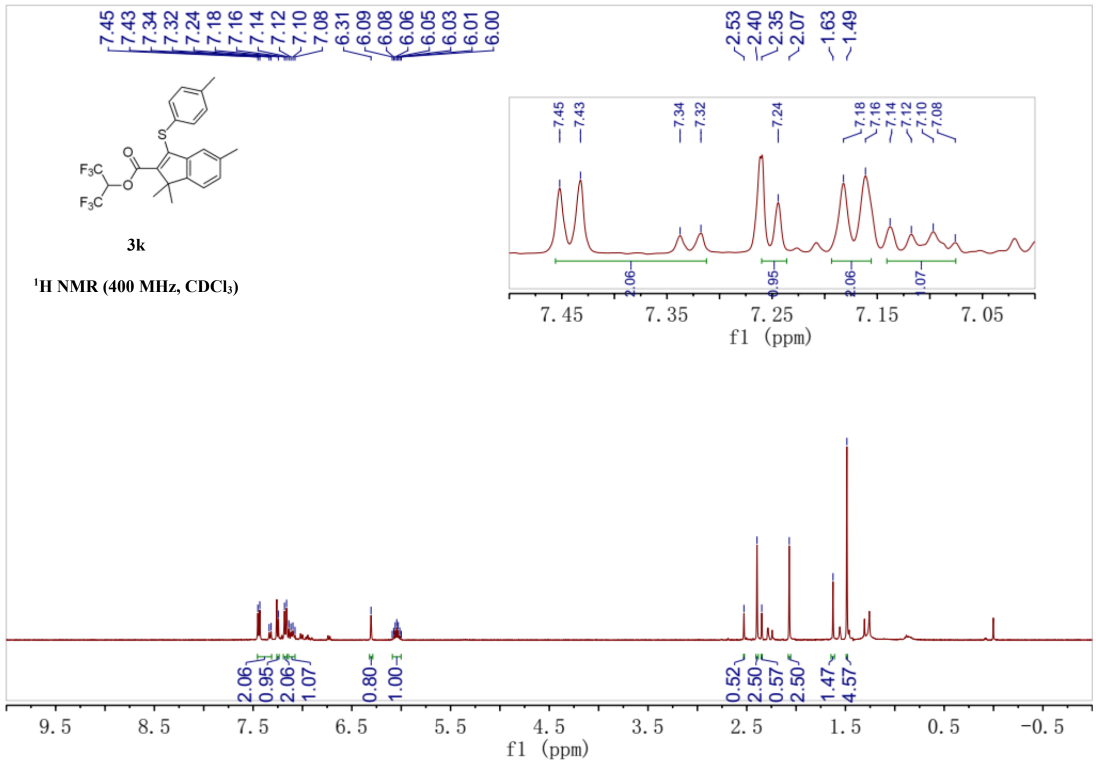
7.45 7.43 7.34 7.32 7.24 7.18 7.16 7.14 7.12 7.10 7.08 6.31 6.09 6.08 6.06 6.05 6.03 6.01 6.00
2.53 2.40 2.35 2.07 1.63 1.49
F_3C
F_3C
S
O
3k
^{1}H NMR (400 MHz, $CDCl_3$)
2.06 0.95 2.06 1.07 0.80 1.00 0.52 2.50 0.57 2.50 1.47 4.57
f1 (ppm)

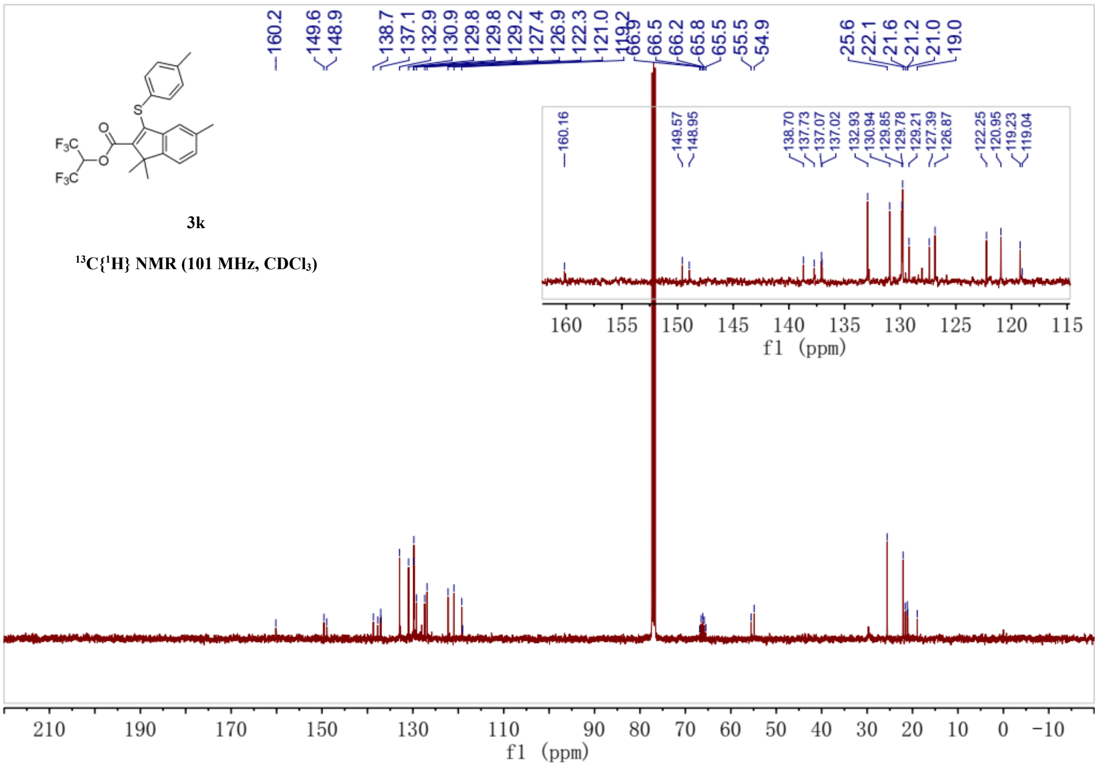
160.2 149.6 148.9 138.7 137.1 132.9 130.9 129.8 129.8 129.2 127.4 126.9 122.3 121.0 119.2 66.9 66.5 66.2 65.8 65.5 55.5 54.9 25.6 22.1 21.6 21.2 21.0 19.0
160.16 149.57 148.95 138.70 137.73 137.07 137.02 132.93 130.94 129.85 129.78 129.21 127.39 126.87 122.25 120.95 119.23 119.04
F_3C
F_3C
S
O
3k
^{13}C{^{1}H} NMR (101 MHz, $CDCl_3$)
f1 (ppm)

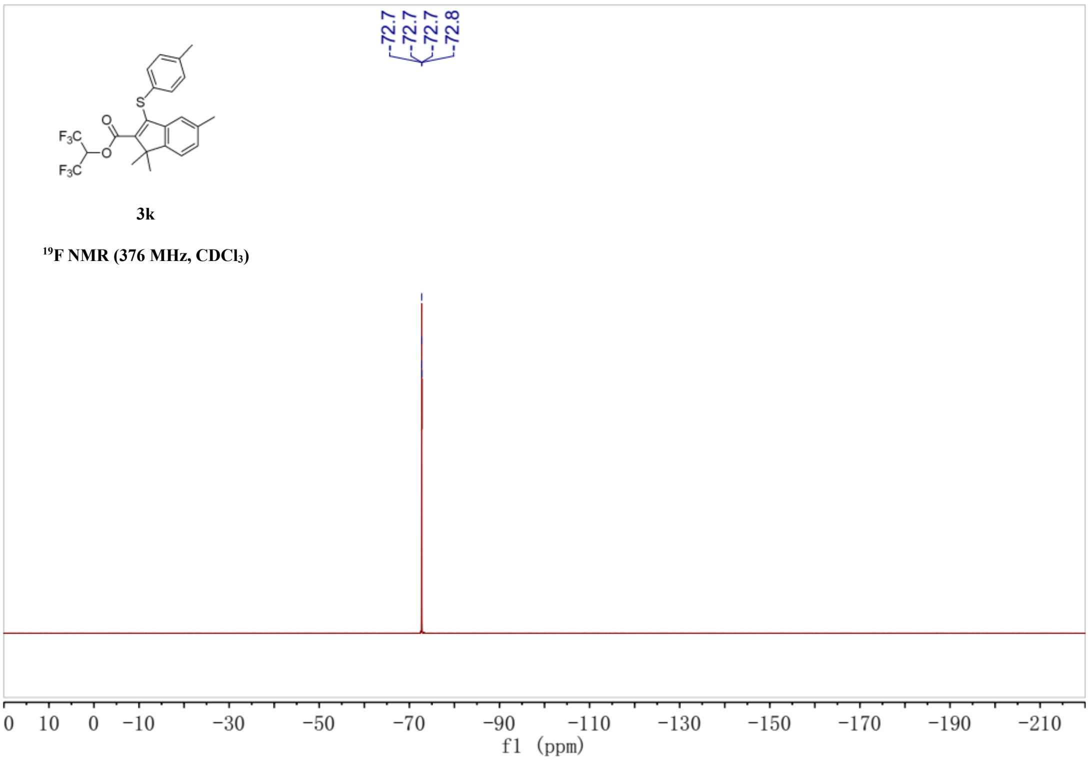
-72.7
-72.7
-72.7
-72.8
3k
19F NMR (376 MHz, CDCl3)
0
10
0
−10
−30
−50
−70
−90
−110
−130
−150
−170
−190
−210
f1 (ppm)

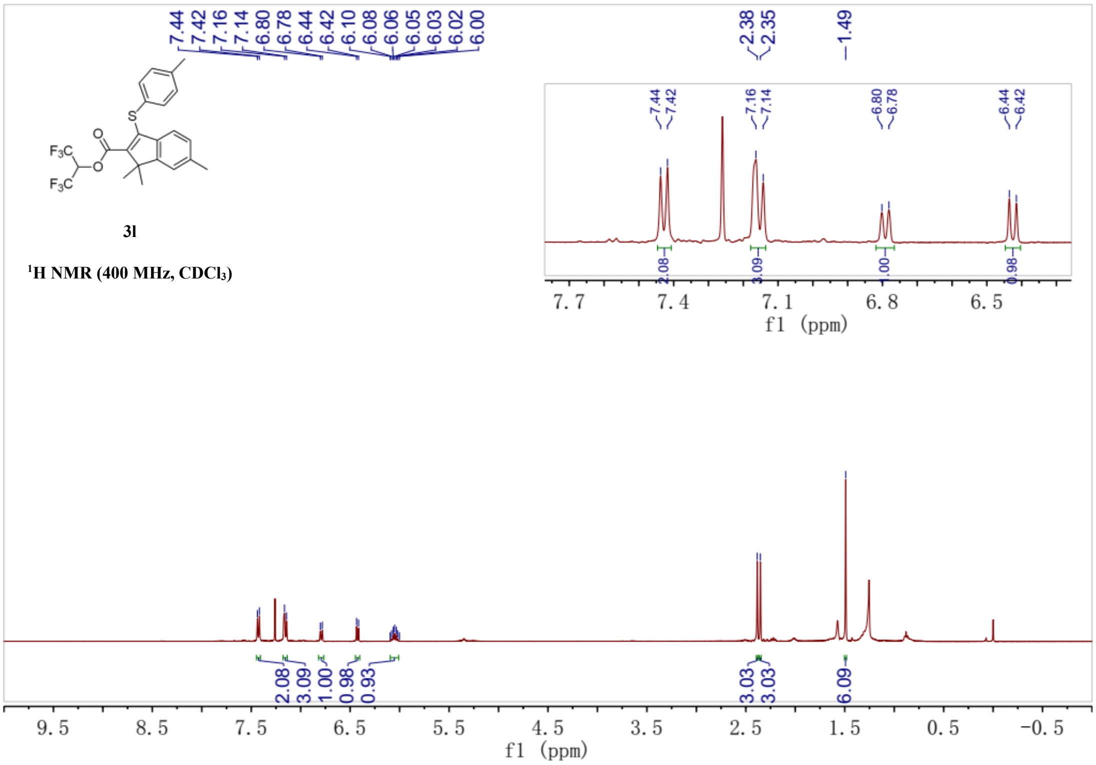
3l
1H NMR (400 MHz, CDCl3)
f1 (ppm)

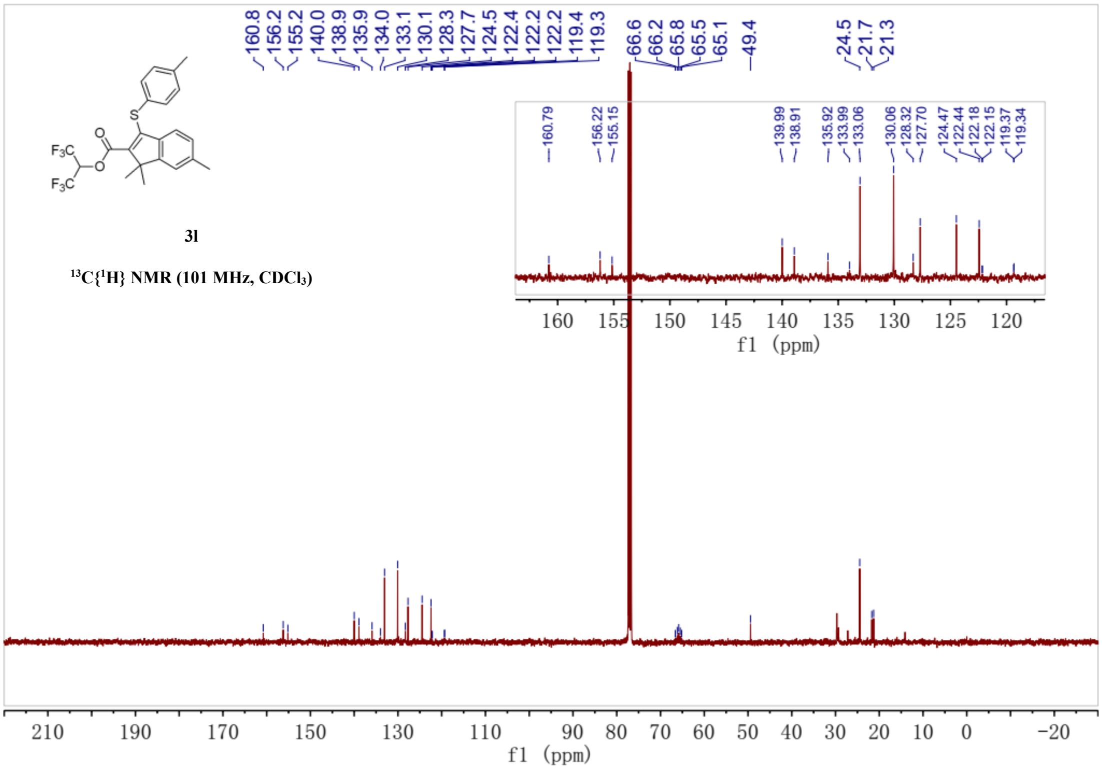
3l
13C{1H} NMR (101 MHz, CDCl3)
f1 (ppm)

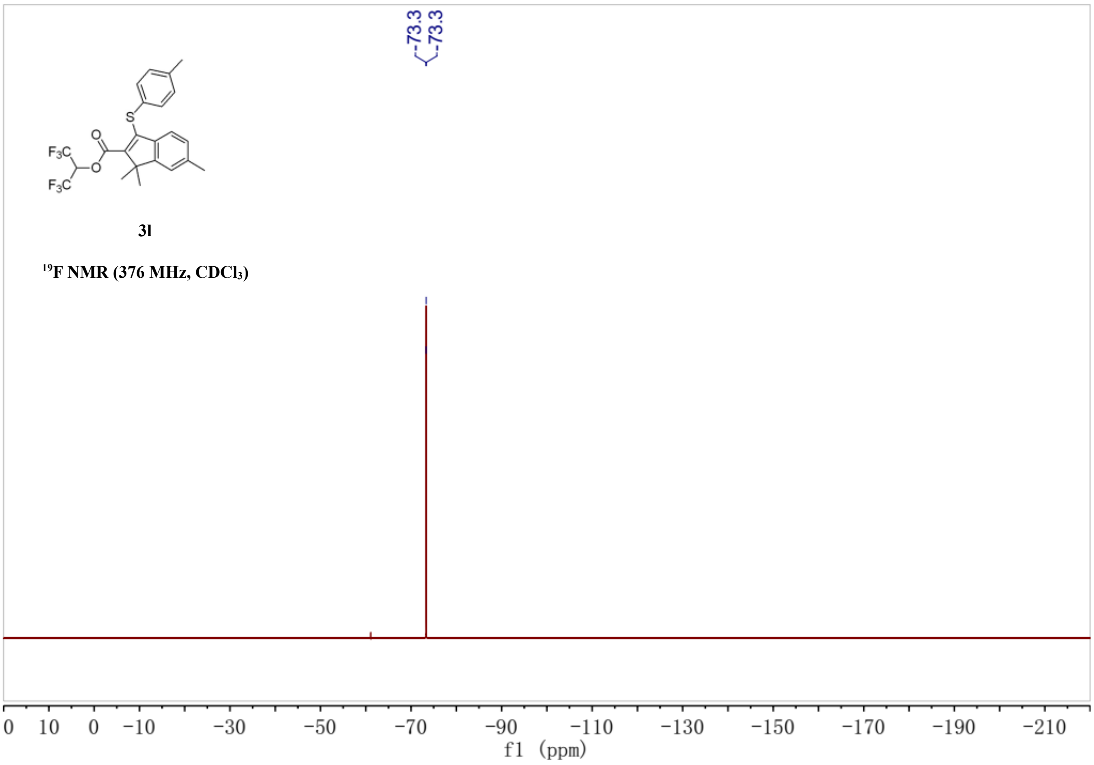
-73.3
-73.3
F3C
F3C
O
O
S
3l
19F NMR (376 MHz, CDCl3)
0 10 0 −10 −30 −50 −70 −90 −110 −130 −150 −170 −190 −210
f1 (ppm)

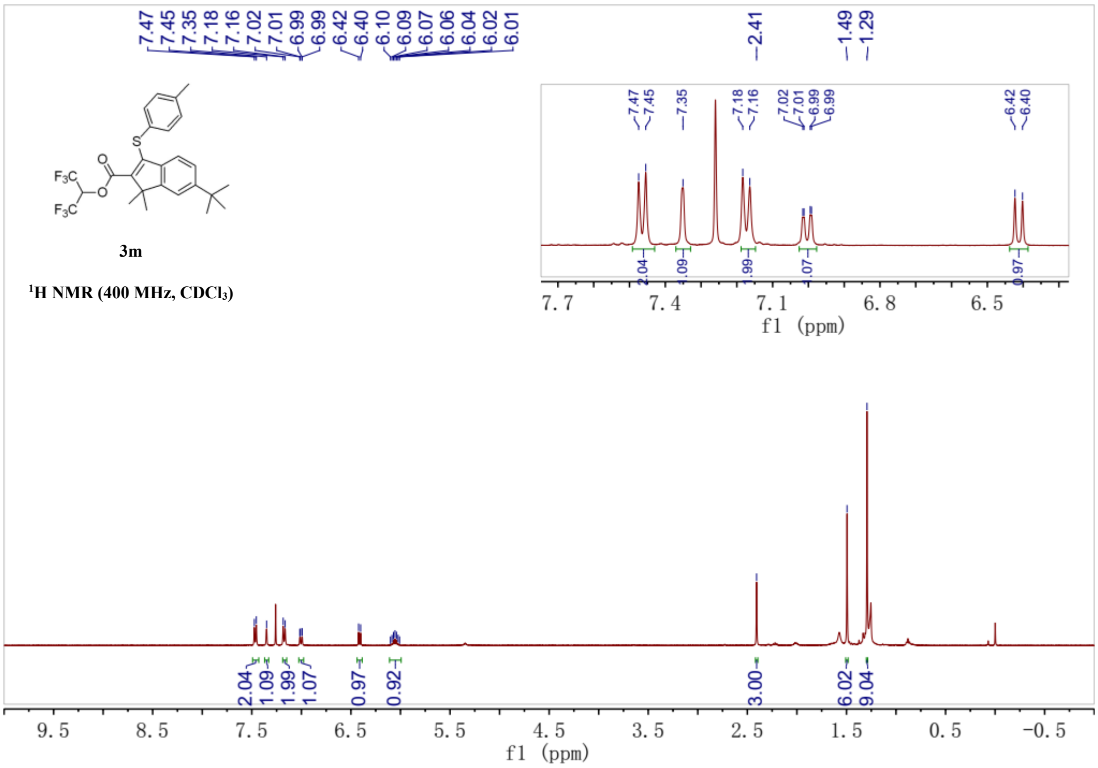
7.47 7.45 7.35 7.18 7.16 7.02 7.01 6.99 6.99 6.42 6.40 6.10 6.09 6.07 6.06 6.04 6.02 6.01
2.41
1.49 1.29
F3C
F3C
S
O
O
3m
1H NMR (400 MHz, CDCl3)
2.04 1.09 1.99 1.07 0.97 0.92 3.00 6.02 9.04
f1 (ppm)

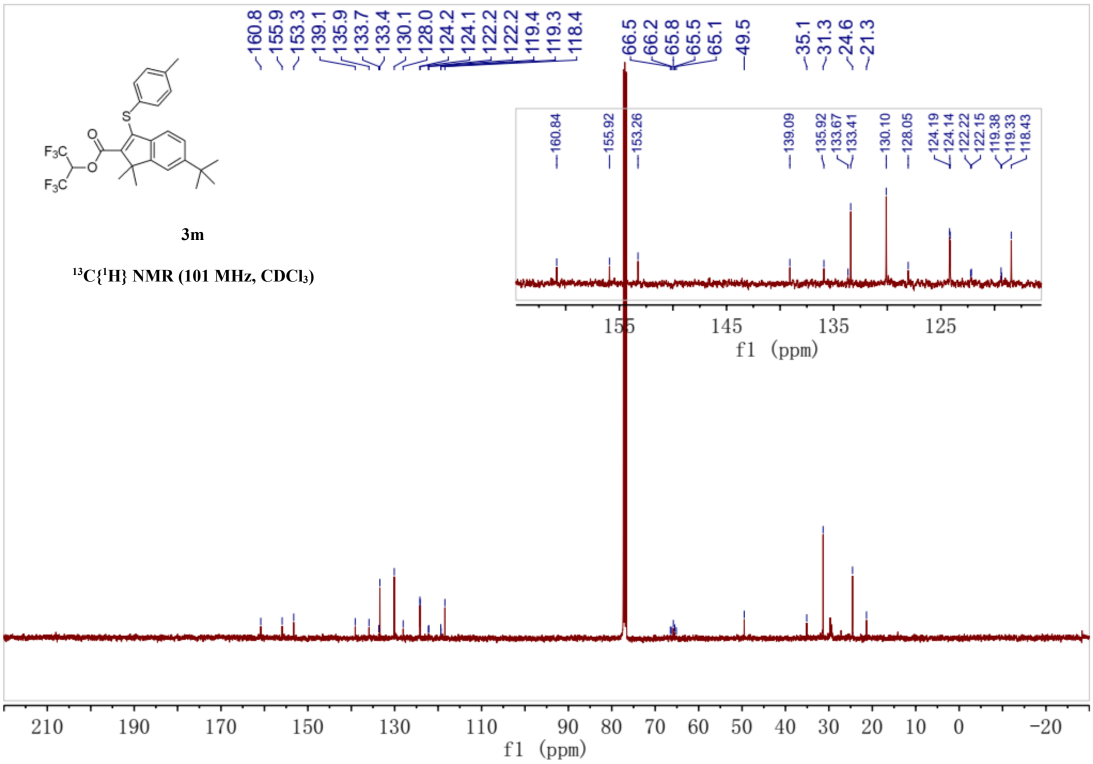
160.8 155.9 153.3 139.1 135.9 133.7 133.4 130.1 128.0 124.2 124.1 122.2 122.2 119.4 119.3 118.4
66.5 66.2 65.8 65.5 65.1 49.5
35.1 31.3 24.6 21.3
160.84 155.92 153.26 139.09 135.92 133.67 133.41 130.10 128.05 124.19 124.14 122.22 122.15 119.38 119.33 118.43
F3C
F3C
S
O
O
3m
13C{1H} NMR (101 MHz, CDCl3)
f1 (ppm)

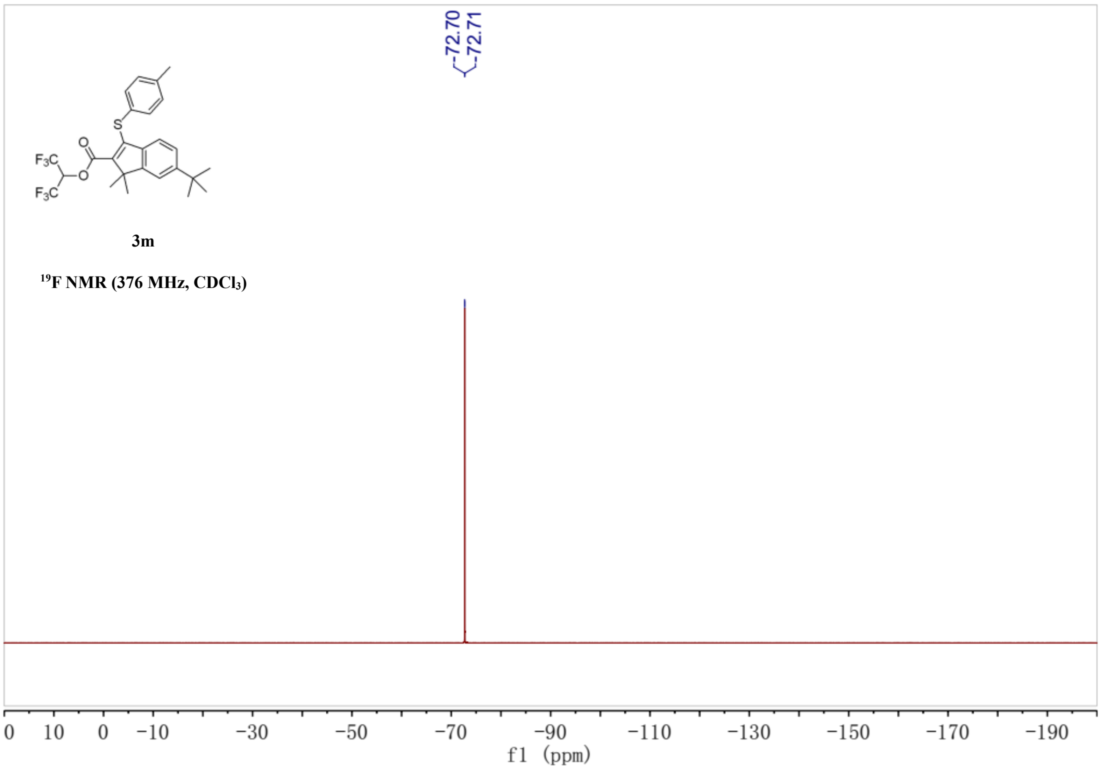
-72.70
-72.71
3m
19F NMR (376 MHz, CDCl3)
0 10 0 −10 −30 −50 −70 −90 −110 −130 −150 −170 −190
f1 (ppm)

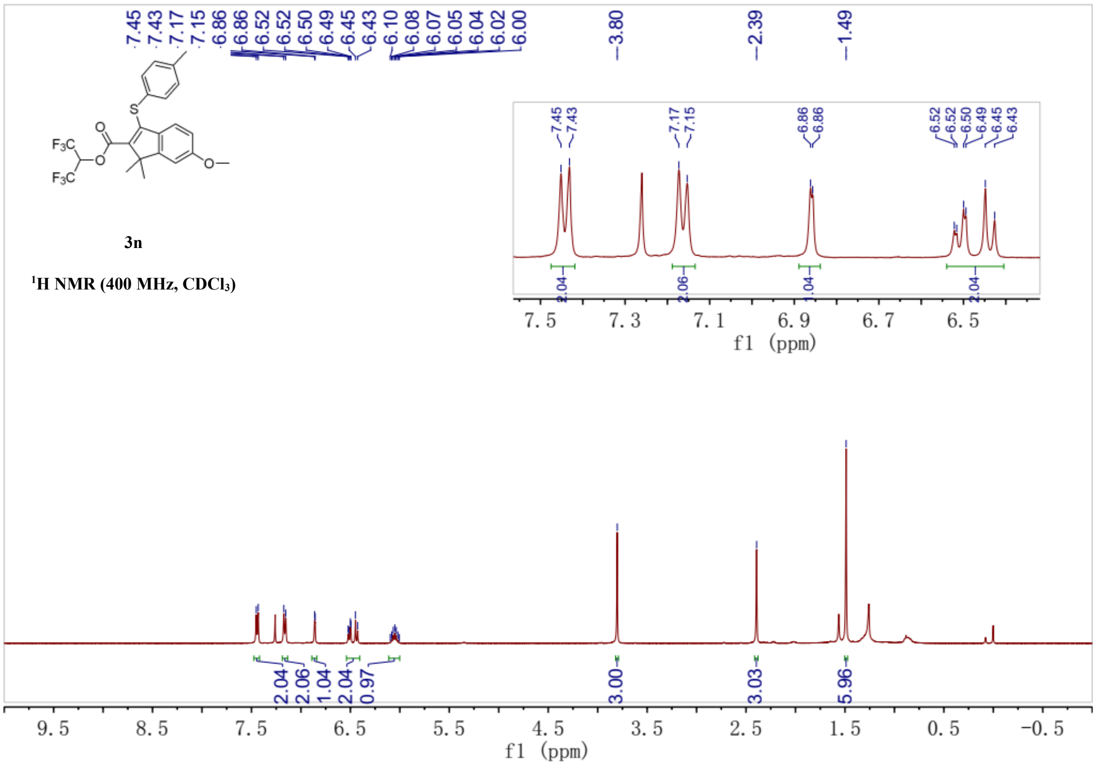

3n

^{1}H NMR (400 MHz, $CDCl_3$)

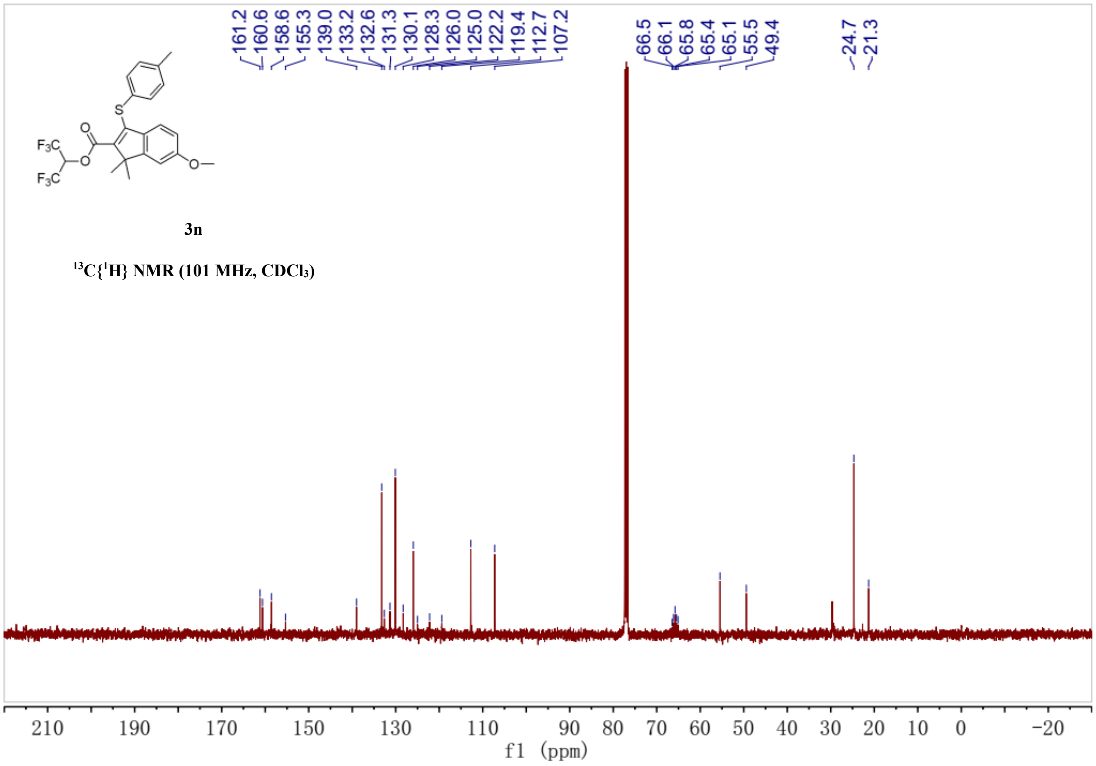

3n

^{13}C{^{1}H} NMR (101 MHz, $CDCl_3$)

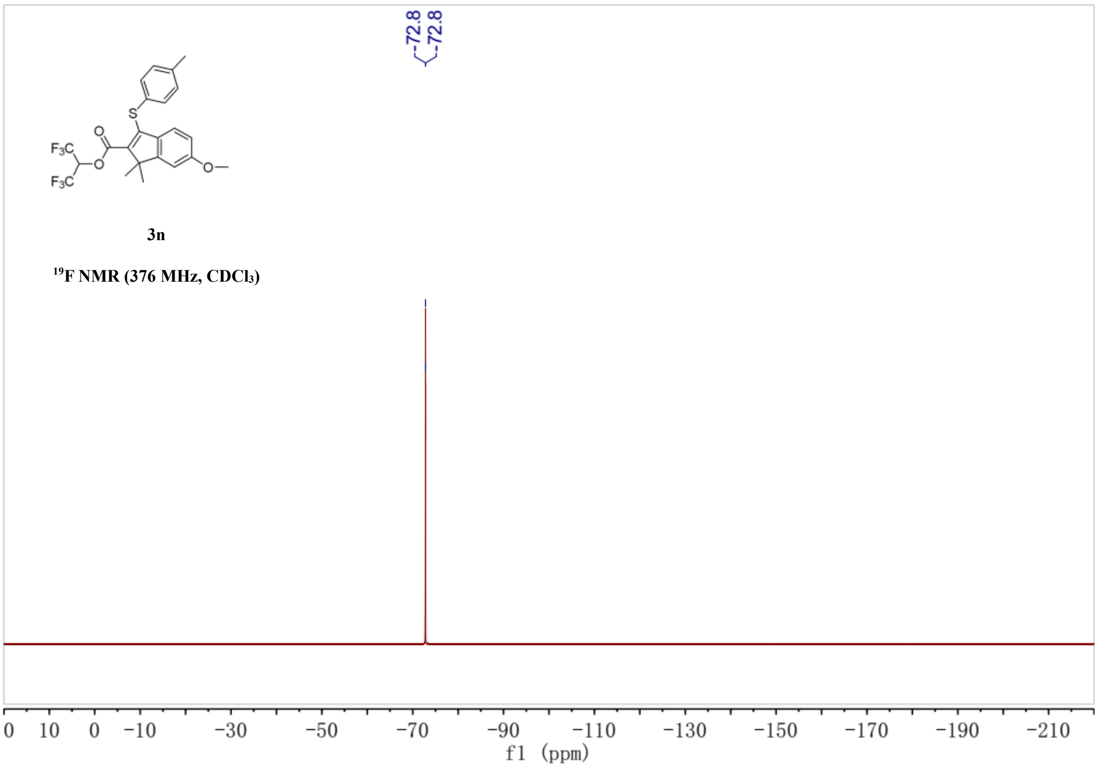

-72.8
-72.8
F3C
F3C
O
O
S
O
3n
19F NMR (376 MHz, CDCl3)
0
10
0
−10
−30
−50
−70
−90
−110
−130
−150
−170
−190
−210
f1 (ppm)

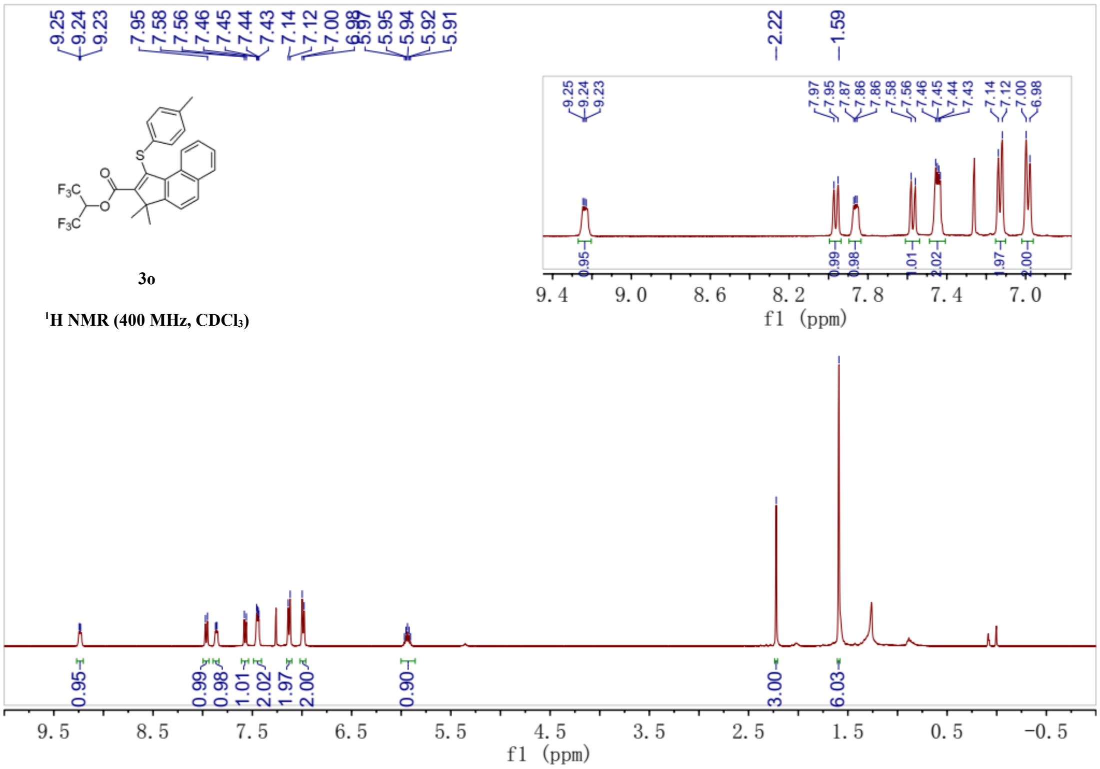
3o
¹H NMR (400 MHz, CDCl₃)
f1 (ppm)

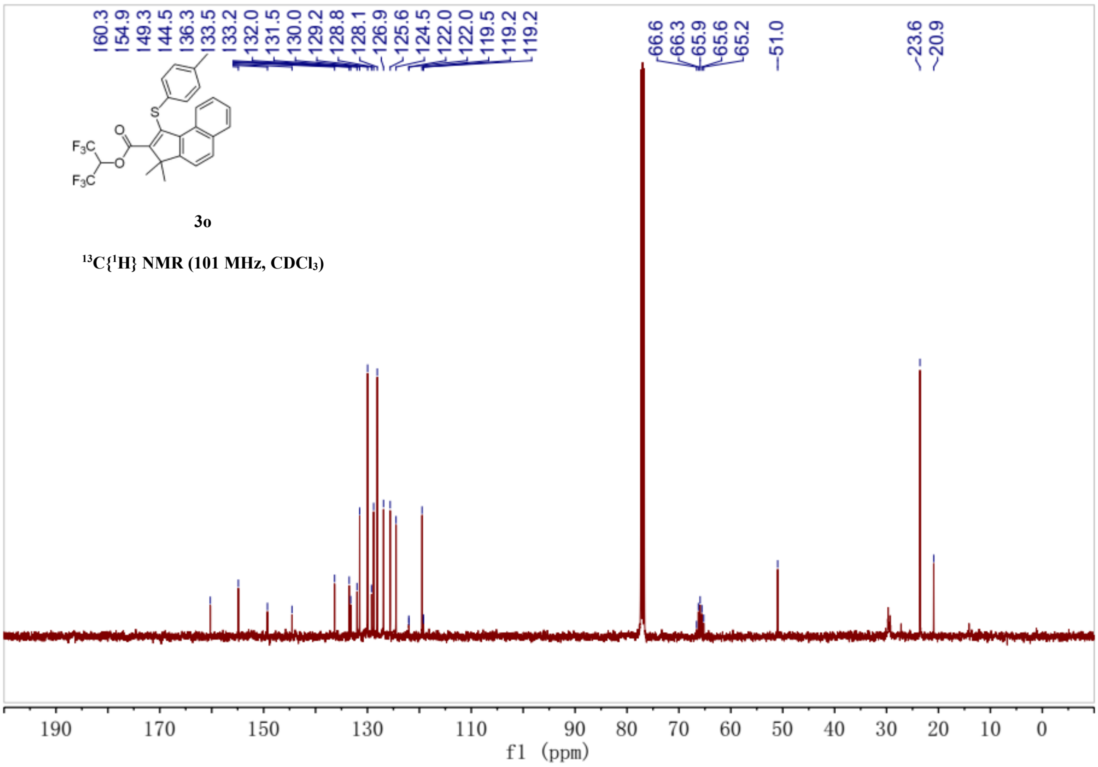
3o
¹³C{¹H} NMR (101 MHz, CDCl₃)
f1 (ppm)

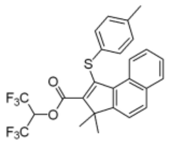

3o

19F NMR (376 MHz, CDCl3)

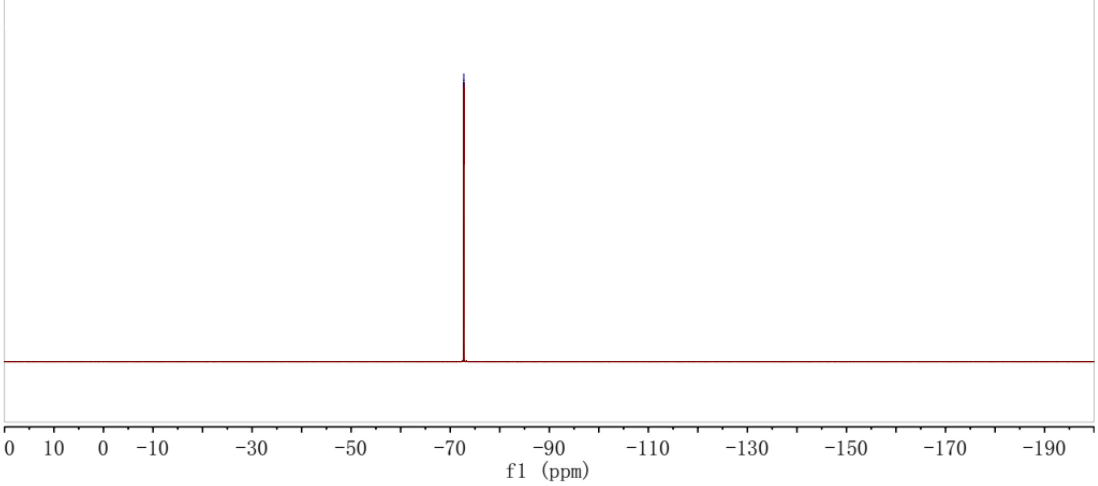

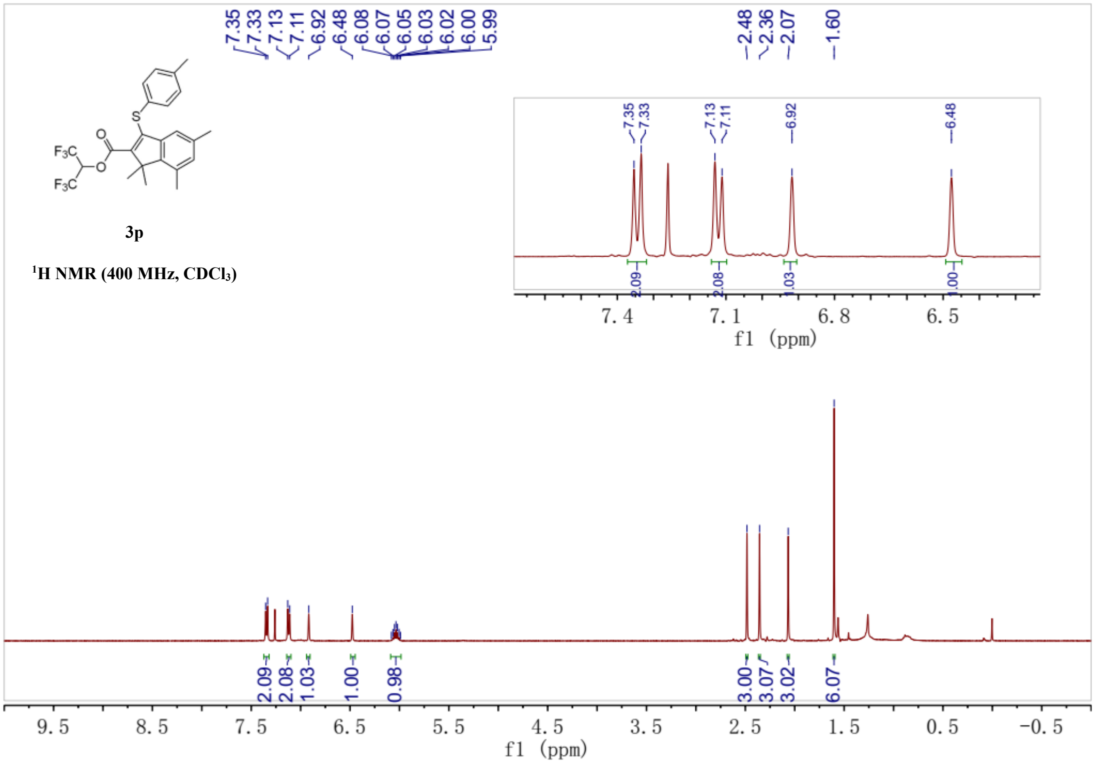

7.35 7.33 7.13 7.11 6.92 6.48 6.08 6.07 6.05 6.03 6.02 6.00 5.99
2.48 2.36 2.07
1.60
S
O
O
F3C
F3C
3p
1H NMR (400 MHz, CDCl3)
2.09 2.08 1.03 1.00 0.98
3.00 3.07 3.02 6.07
f1 (ppm)

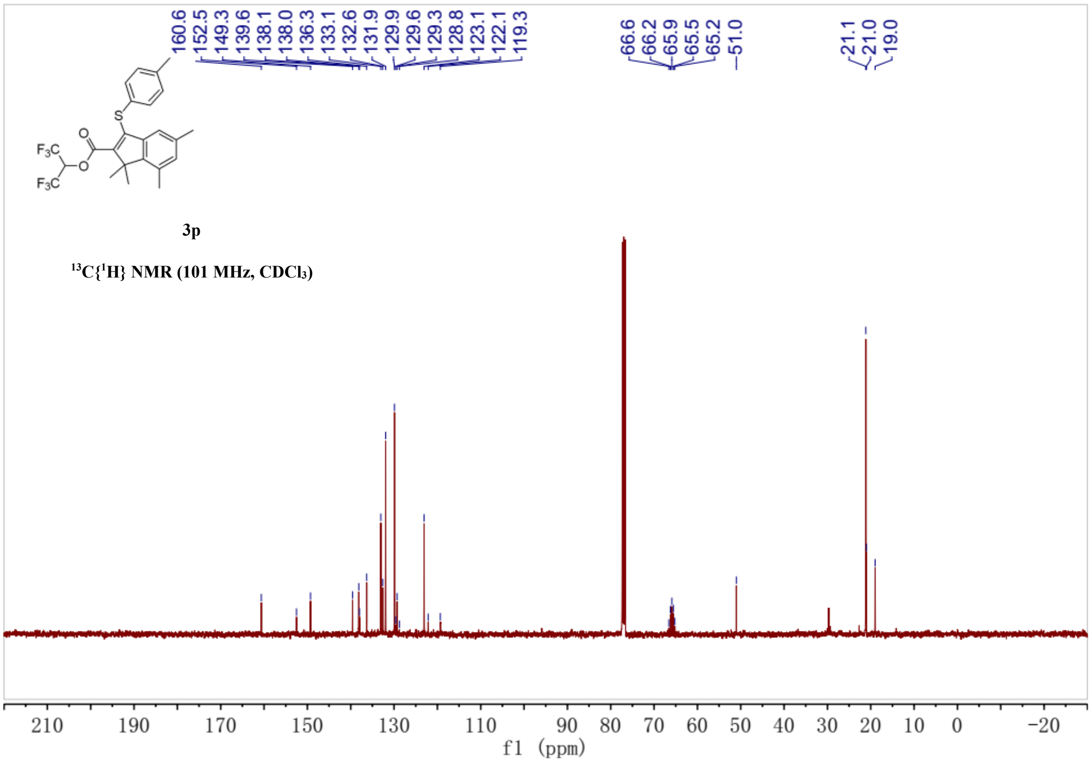

160.6 152.5 149.3 139.6 138.1 138.0 136.3 133.1 132.6 131.9 129.9 129.6 129.3 128.8 123.1 122.1 119.3
66.6 66.2 65.9 65.5 65.2
51.0
21.1 21.0 19.0
S
O
O
F3C
F3C
3p
13C{1H} NMR (101 MHz, CDCl3)
f1 (ppm)

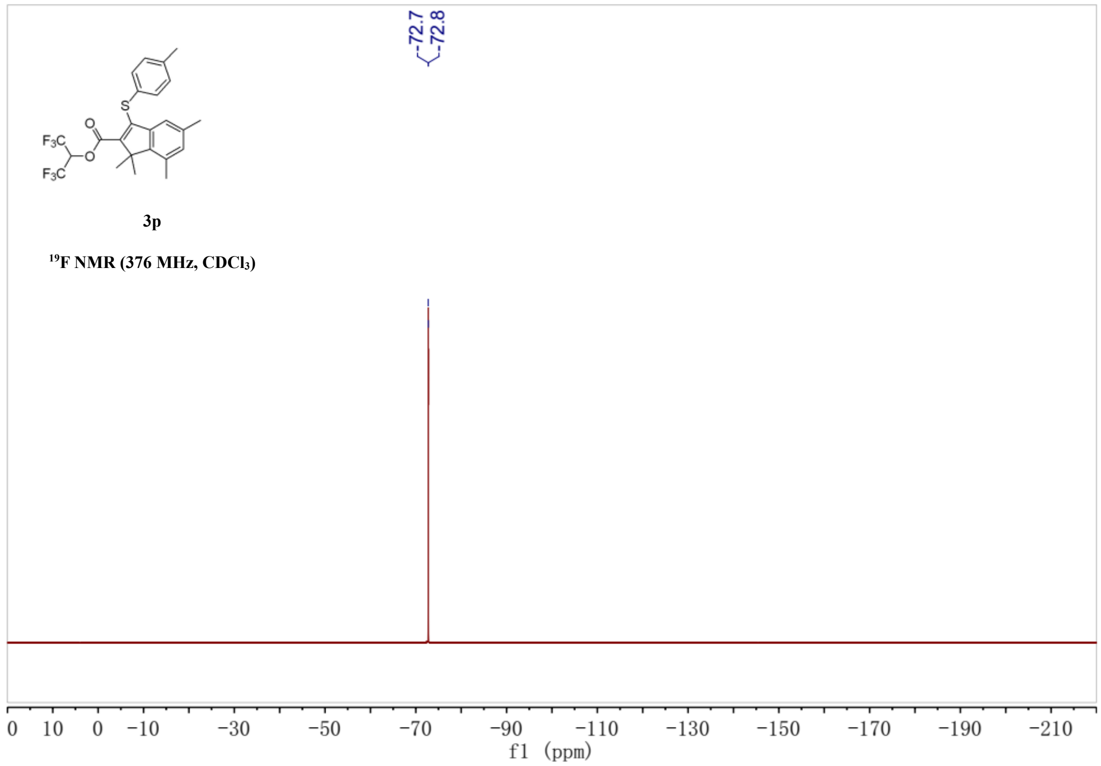
-72.7
-72.8
F3C
O
S
F3C
3p
19F NMR (376 MHz, CDCl3)
0 10 0 −10 −30 −50 −70 −90 −110 −130 −150 −170 −190 −210
f1 (ppm)

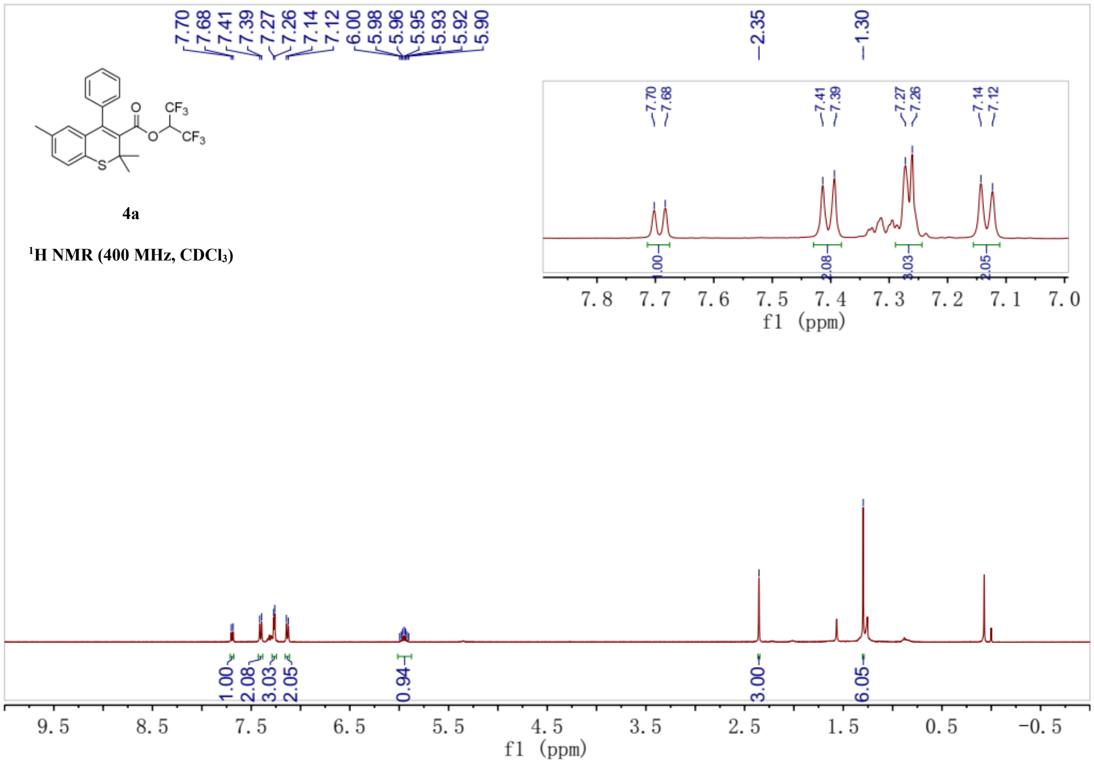

4a
1H NMR (400 MHz, CDCl3)
7.70 7.68 7.41 7.39 7.27 7.26 7.14 7.12 6.00 5.98 5.96 5.95 5.93 5.92 5.90
2.35
1.30
1.00 2.08 3.03 2.05
0.94
3.00
6.05
f1 (ppm)

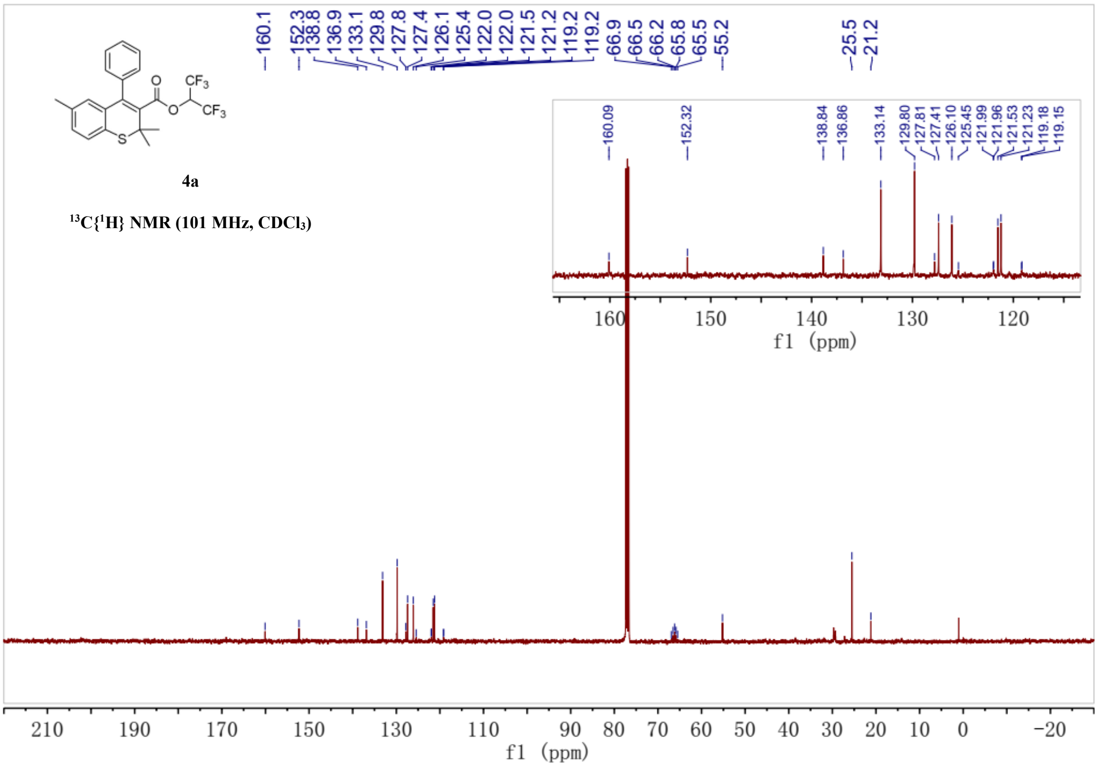

4a
13C{1H} NMR (101 MHz, CDCl3)
160.1 152.3 138.8 136.9 133.1 129.8 127.8 127.4 126.1 125.4 122.0 122.0 121.5 121.2 119.2 119.2 66.9 66.5 66.2 65.8 65.5 55.2
25.5 21.2
160.09 152.32 138.84 136.86 133.14 129.80 127.81 127.41 126.10 125.45 121.99 121.96 121.53 121.23 119.18 119.15
f1 (ppm)

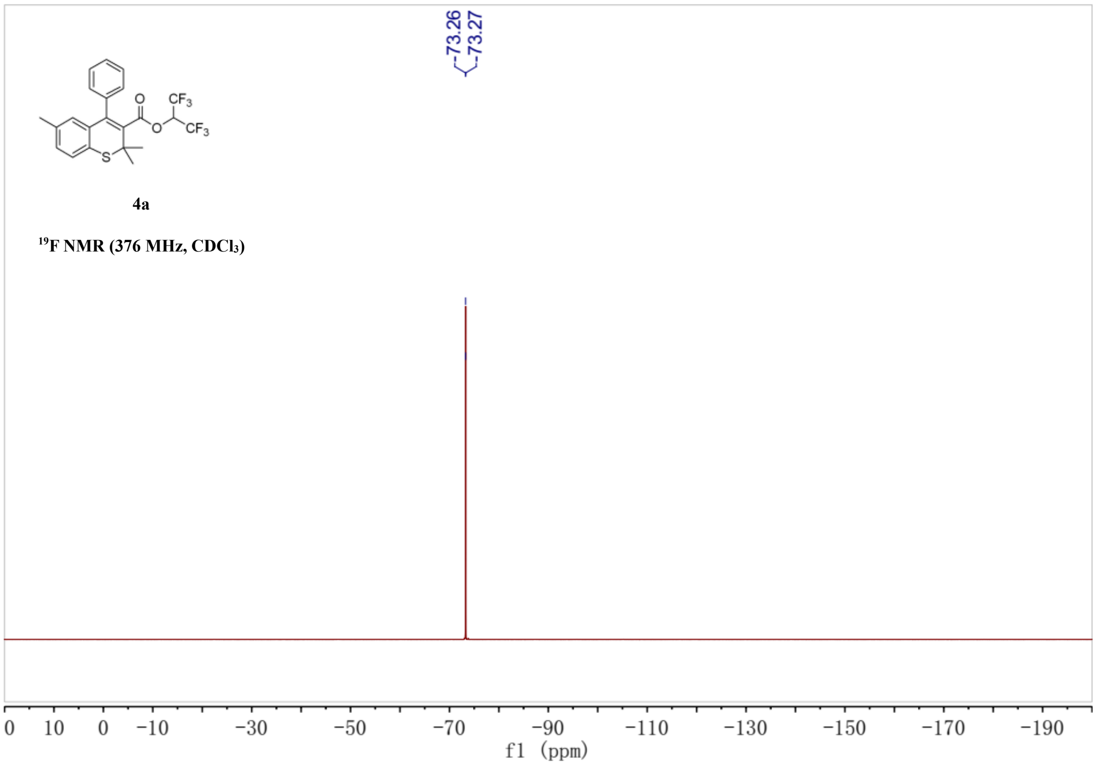
-73.26
-73.27
CF3
CF3
O
O
S
4a
19F NMR (376 MHz, CDCl3)
0
10
0
−10
−30
−50
−70
−90
−110
−130
−150
−170
−190
f1 (ppm)

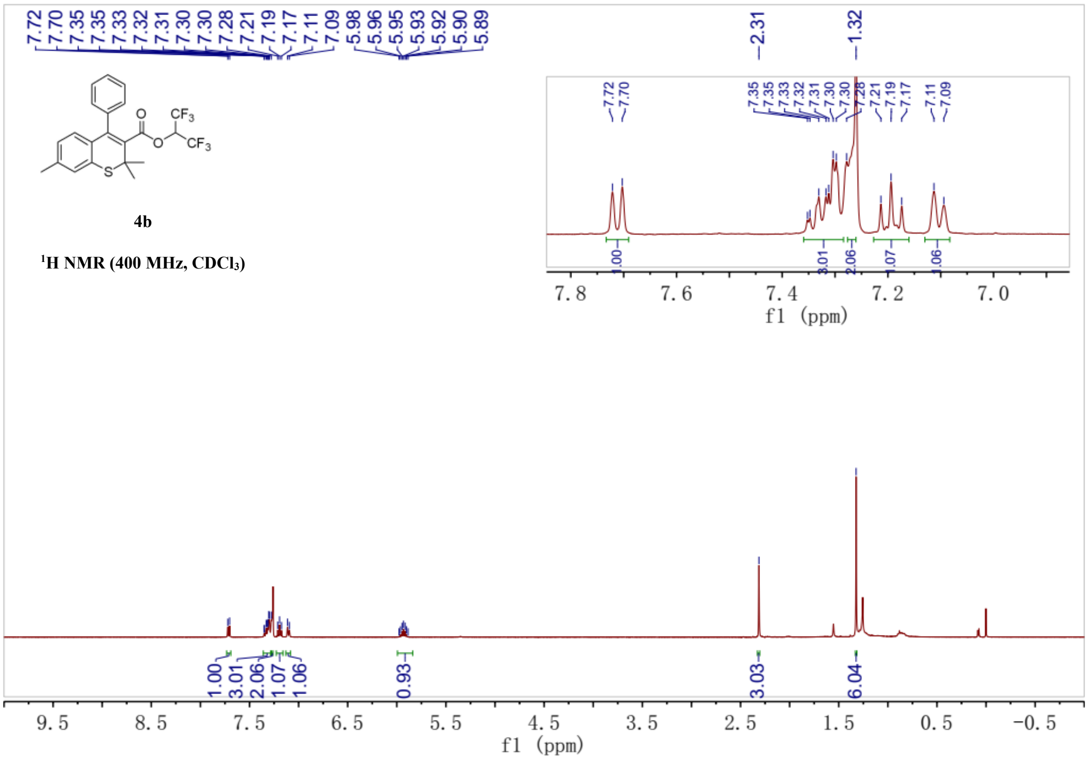
7.72 7.70 7.35 7.35 7.33 7.32 7.31 7.30 7.30 7.28 7.21 7.19 7.17 7.11 7.09 5.98 5.96 5.95 5.93 5.92 5.90 5.89
2.31
1.32
4b
^{1}H NMR (400 MHz, CDCl$_3$)
1.00 3.01 2.06 1.07 1.06
0.93
3.03
6.04
9.5 8.5 7.5 6.5 5.5 4.5 3.5 2.5 1.5 0.5 −0.5
f1 (ppm)
7.8 7.6 7.4 7.2 7.0
f1 (ppm)

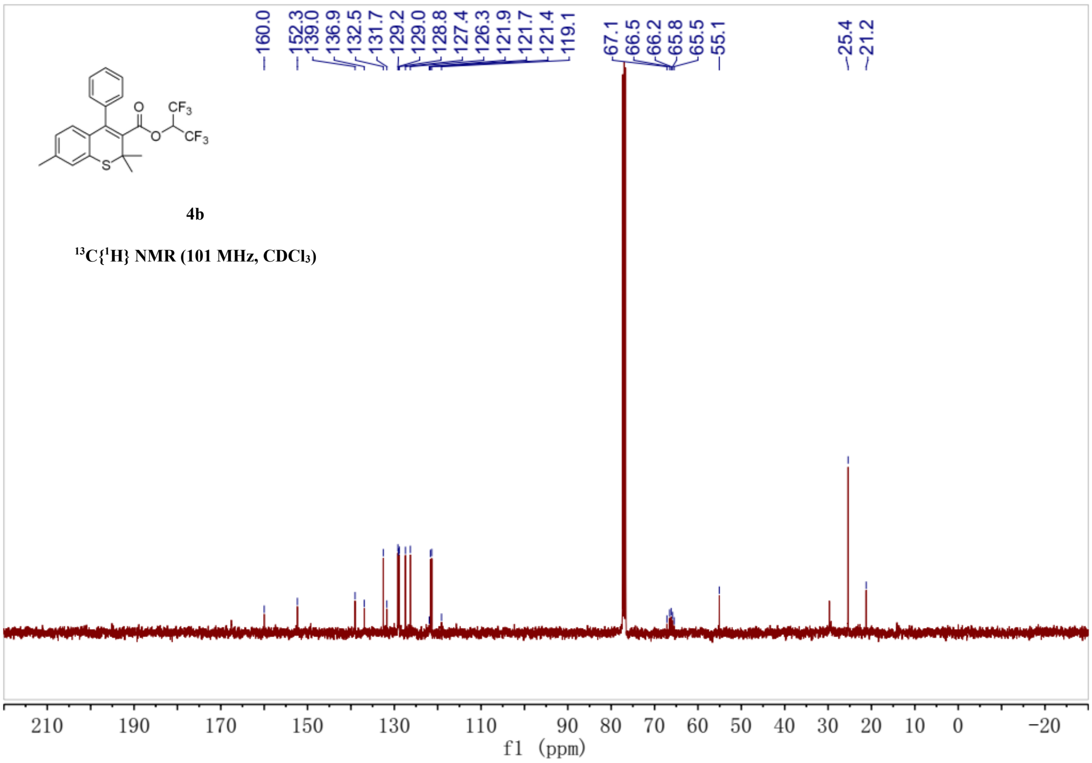
160.0
152.3 139.0 136.9 132.5 131.7 129.2 129.0 128.8 127.4 126.3 121.9 121.7 121.4 119.1
67.1 66.5 66.2 65.8 65.5 55.1
25.4 21.2
4b
^{13}C{^{1}H} NMR (101 MHz, CDCl$_3$)
210 190 170 150 130 110 90 80 70 60 50 40 30 20 10 0 −20
f1 (ppm)

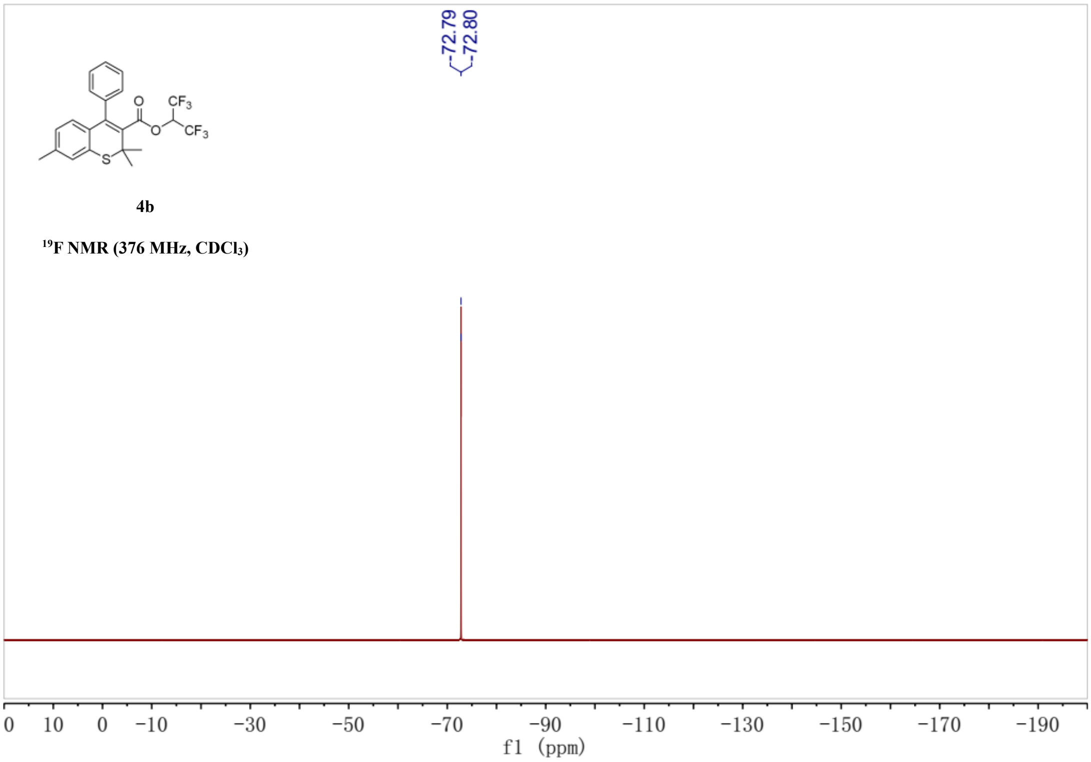
-72.79
-72.80
CF3
O
O
CF3
S
4b
19F NMR (376 MHz, CDCl3)
0 10 0 −10 −30 −50 −70 −90 −110 −130 −150 −170 −190
f1 (ppm)

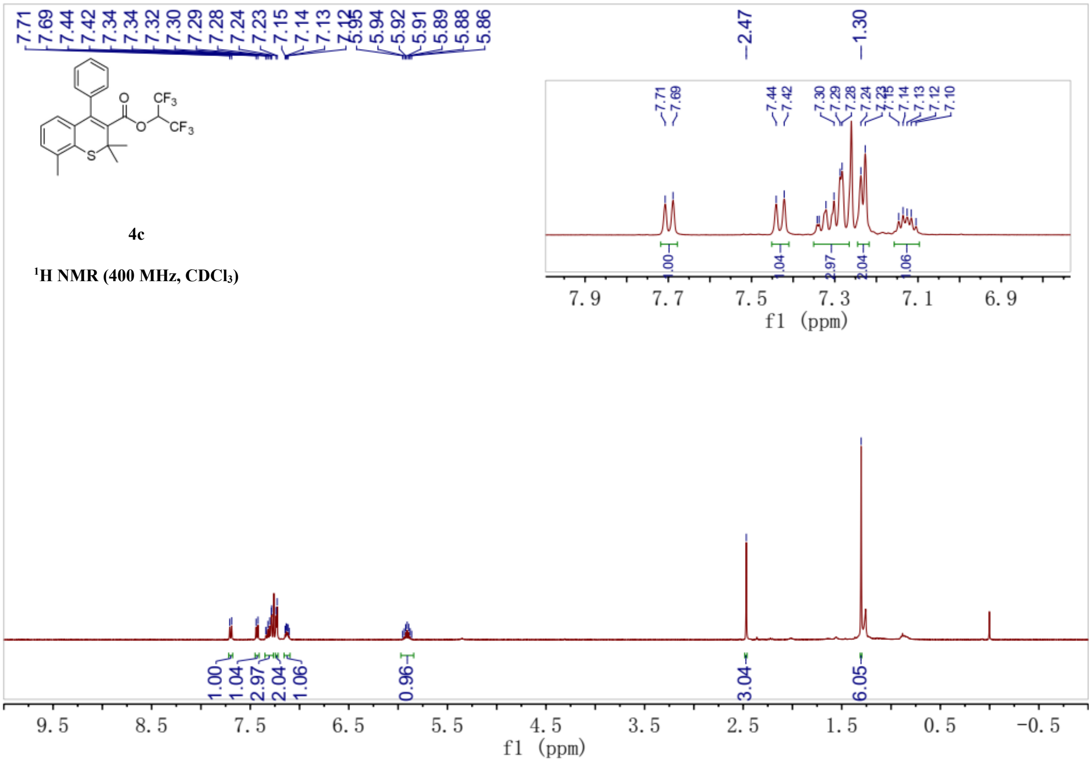
4c
^{1}H NMR (400 MHz, CDCl$_3$)
7.71 7.69 7.44 7.42 7.34 7.34 7.32 7.30 7.29 7.28 7.24 7.23 7.15 7.14 7.13 7.12 5.95 5.94 5.92 5.91 5.89 5.88 5.86
2.47
1.30
1.00 1.04 2.97 2.04 1.06 0.96 3.04 6.05
f1 (ppm)

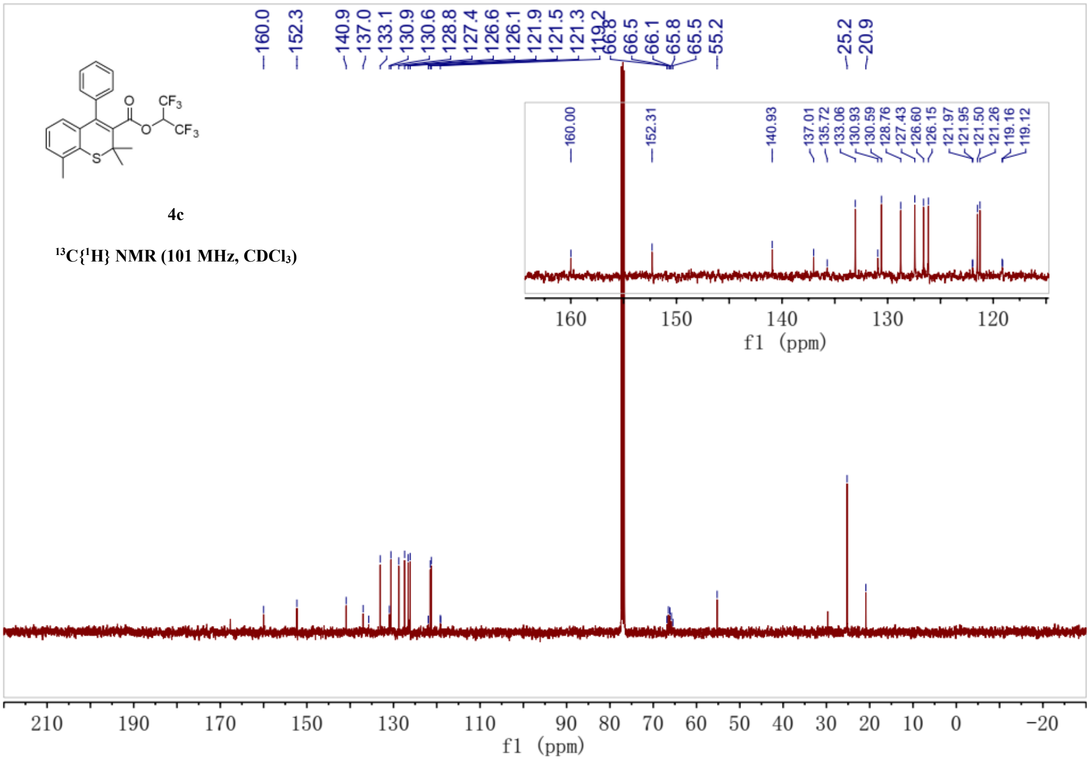
4c
^{13}C{^{1}H} NMR (101 MHz, CDCl$_3$)
160.0 152.3 140.9 137.0 133.1 130.9 130.6 128.8 127.4 126.6 126.1 121.9 121.5 121.3 119.2 66.8 66.5 66.1 65.8 65.5 55.2
25.2 20.9
160.00 152.31 140.93 137.01 135.72 133.06 130.93 130.59 128.76 127.43 126.60 126.15 121.97 121.95 121.50 121.26 119.16 119.12
f1 (ppm)

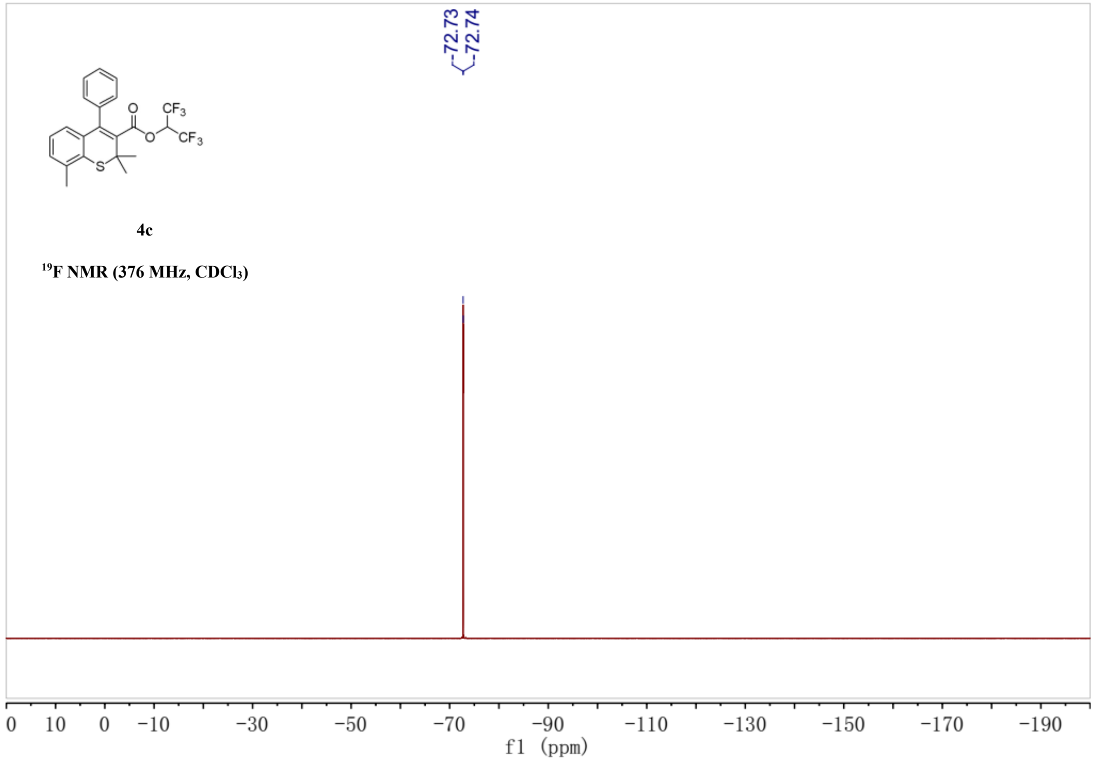
-72.73
-72.74
CF3
O
O
CF3
S
4c
19F NMR (376 MHz, CDCl3)
0
10
0
−10
−30
−50
−70
−90
−110
−130
−150
−170
−190
f1 (ppm)

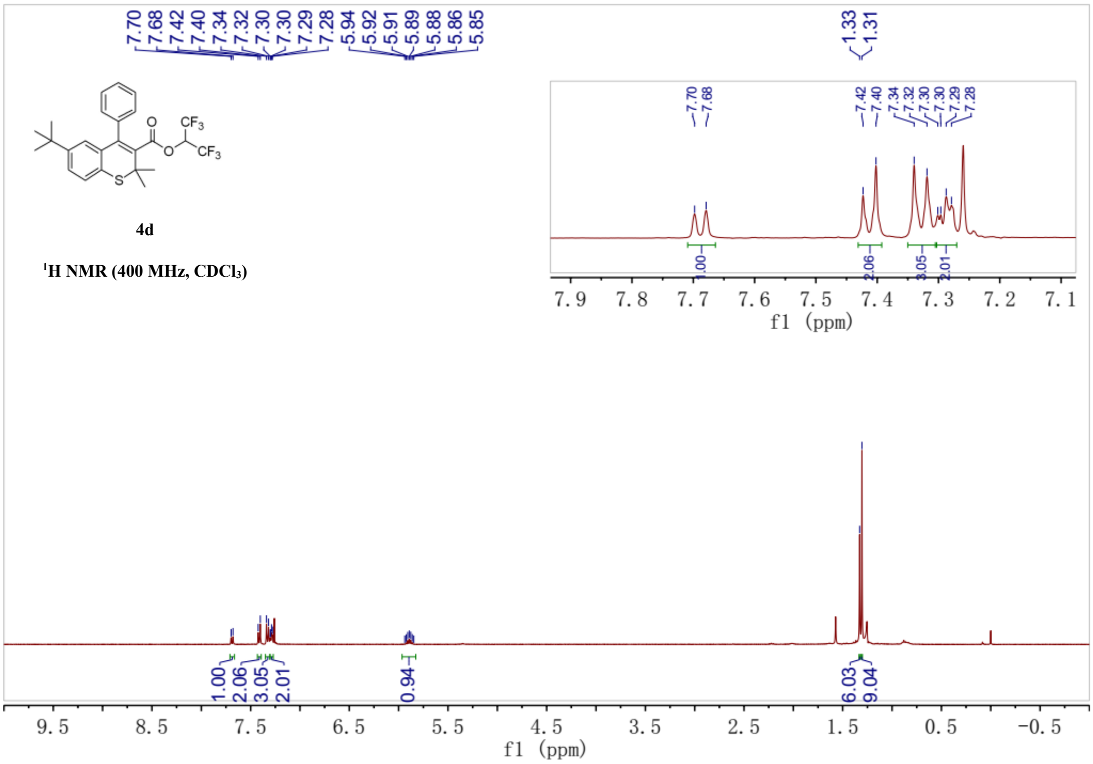

4d

^{1}H NMR (400 MHz, $CDCl_3$)

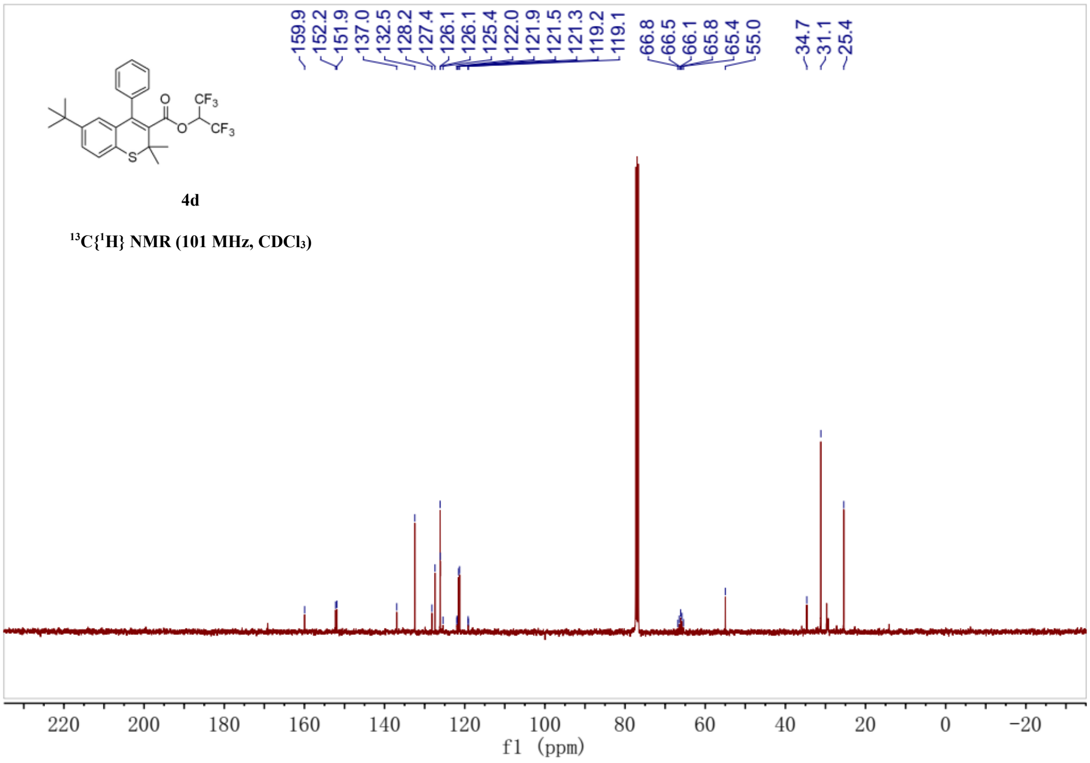

4d

^{13}C{^{1}H} NMR (101 MHz, $CDCl_3$)

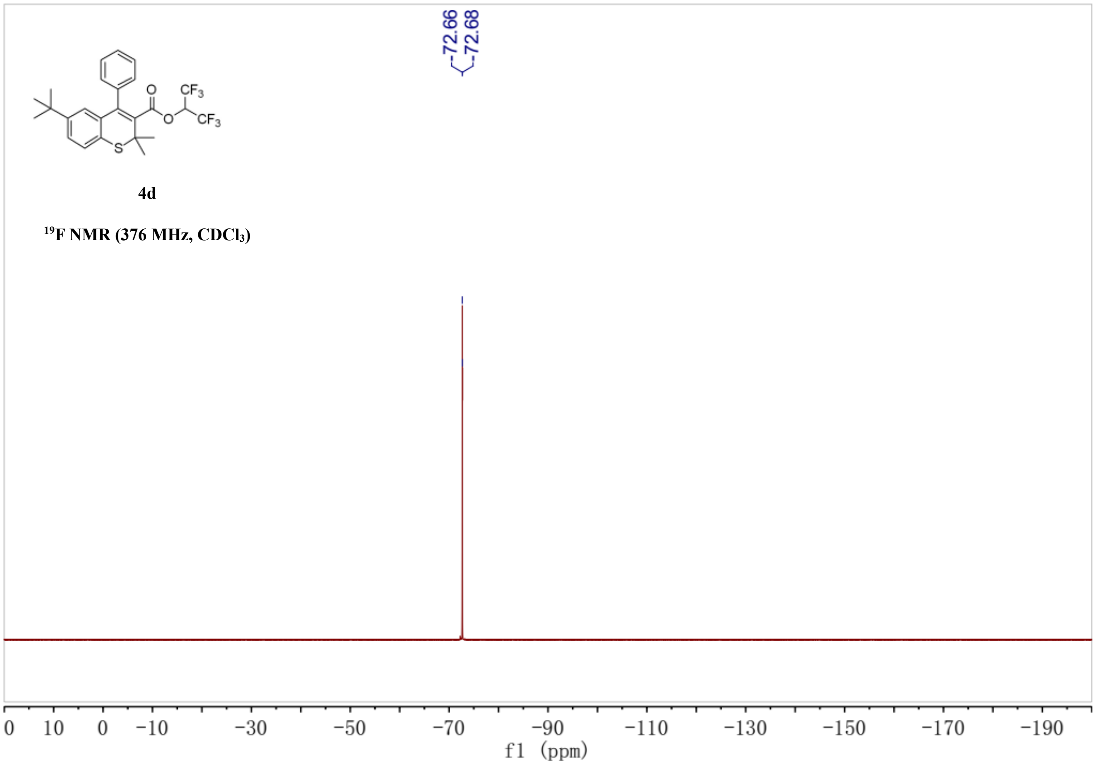
-72.66
-72.68
4d
19F NMR (376 MHz, CDCl3)
0 10 0 −10 −30 −50 −70 −90 −110 −130 −150 −170 −190
f1 (ppm)

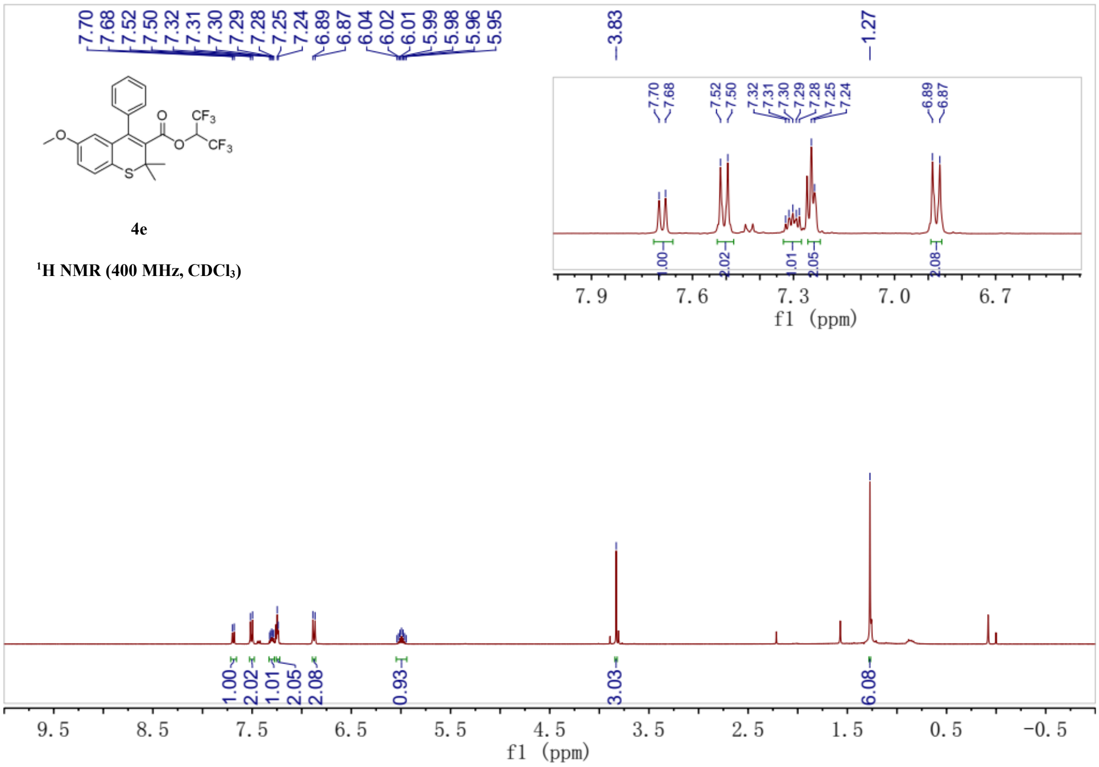
4e
1H NMR (400 MHz, CDCl3)
f1 (ppm)

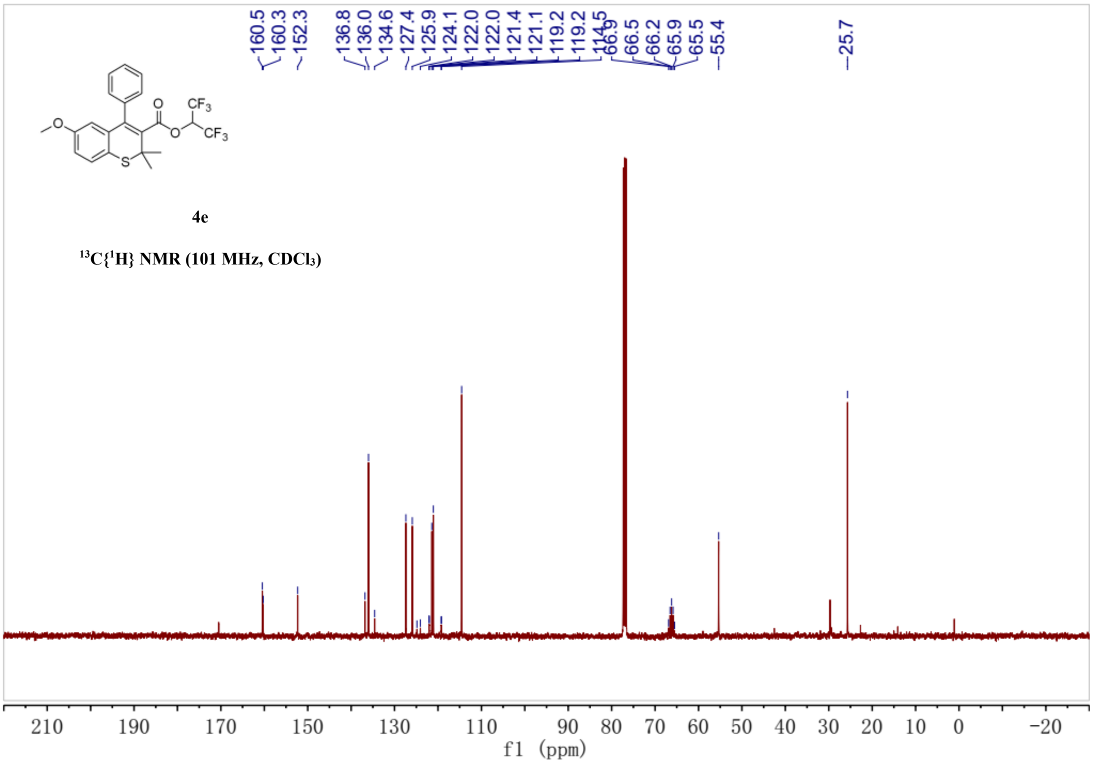
4e
13C{1H} NMR (101 MHz, CDCl3)
f1 (ppm)

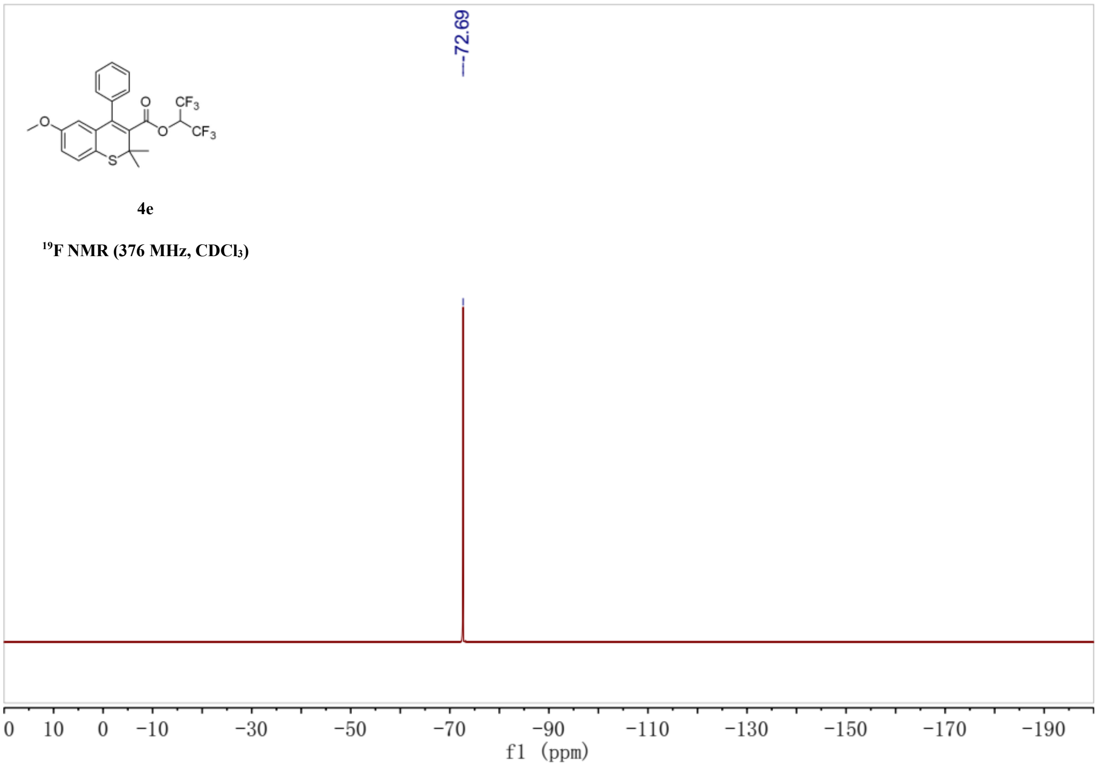

-72.69
CF3
O
O
CF3
O
S
4e
19F NMR (376 MHz, CDCl3)
0 10 0 −10 −30 −50 −70 −90 −110 −130 −150 −170 −190
f1 (ppm)

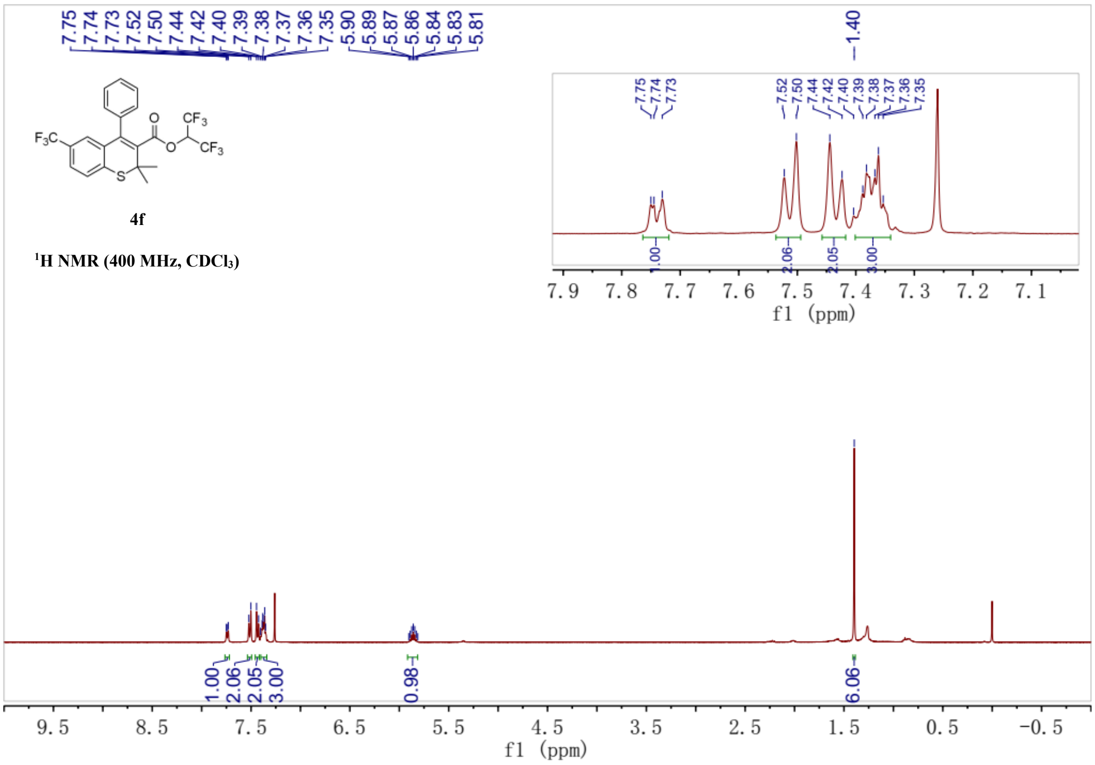
7.75 7.74 7.73 7.52 7.50 7.44 7.42 7.40 7.39 7.38 7.37 7.36 7.35 5.90 5.89 5.87 5.86 5.84 5.83 5.81
1.40
F3C
CF3
CF3
O
S
4f
1H NMR (400 MHz, CDCl3)
1.00 2.06 2.05 3.00
0.98
6.06
9.5 8.5 7.5 6.5 5.5 4.5 3.5 2.5 1.5 0.5 -0.5
f1 (ppm)
7.9 7.8 7.7 7.6 7.5 7.4 7.3 7.2 7.1

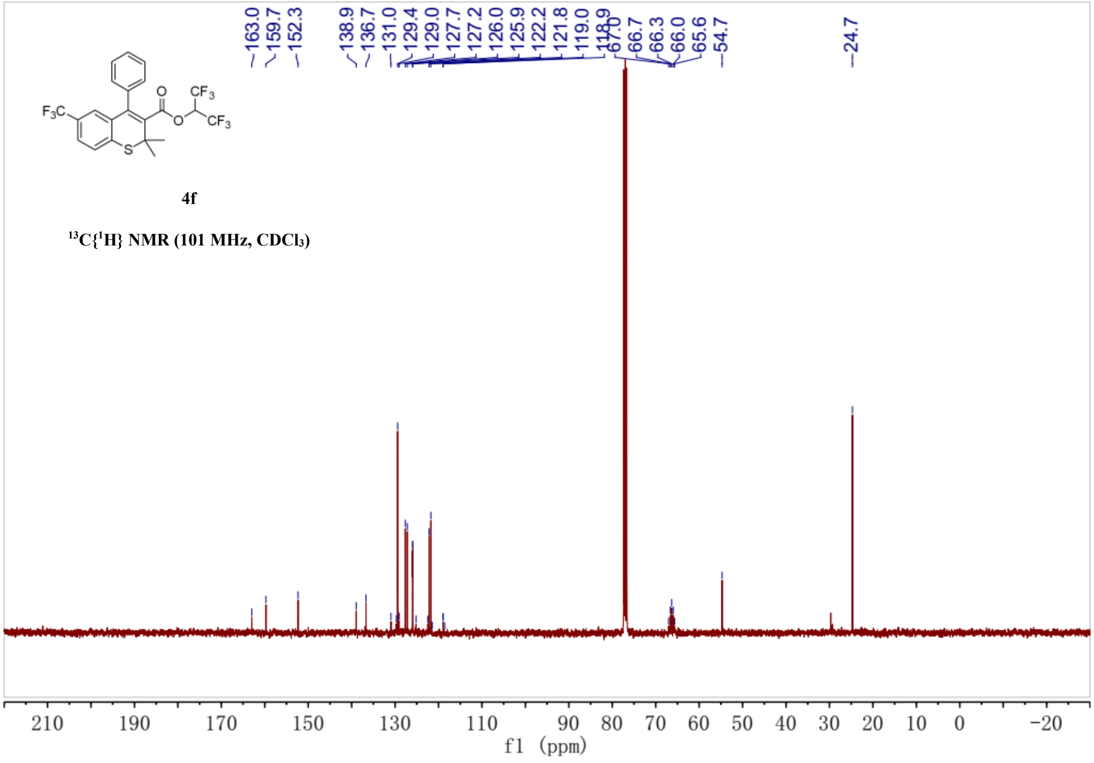
163.0 159.7 152.3 138.9 136.7 131.0 129.4 129.0 127.7 127.2 126.0 125.9 122.2 121.8 119.0 118.9 67.0 66.7 66.3 66.0 65.6 54.7
24.7
F3C
CF3
CF3
O
S
4f
13C{1H} NMR (101 MHz, CDCl3)
210 190 170 150 130 110 90 80 70 60 50 40 30 20 10 0 -20
f1 (ppm)

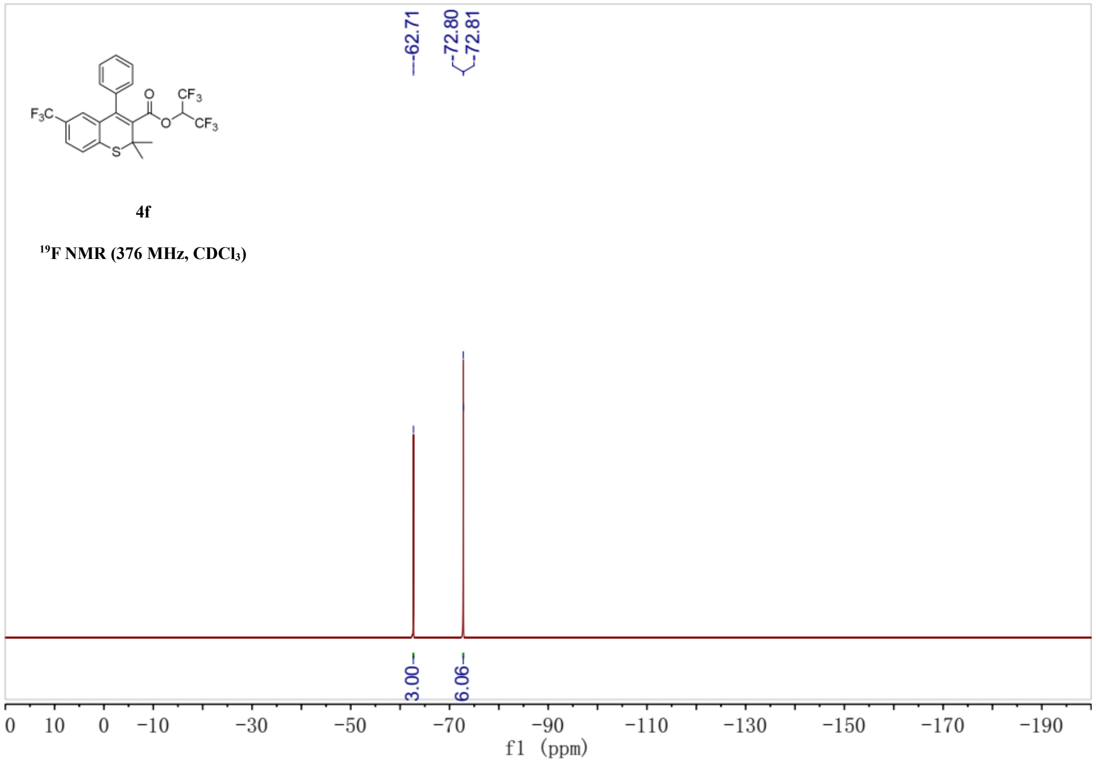

4f

^{19}F NMR (376 MHz, CDCl$_3$)

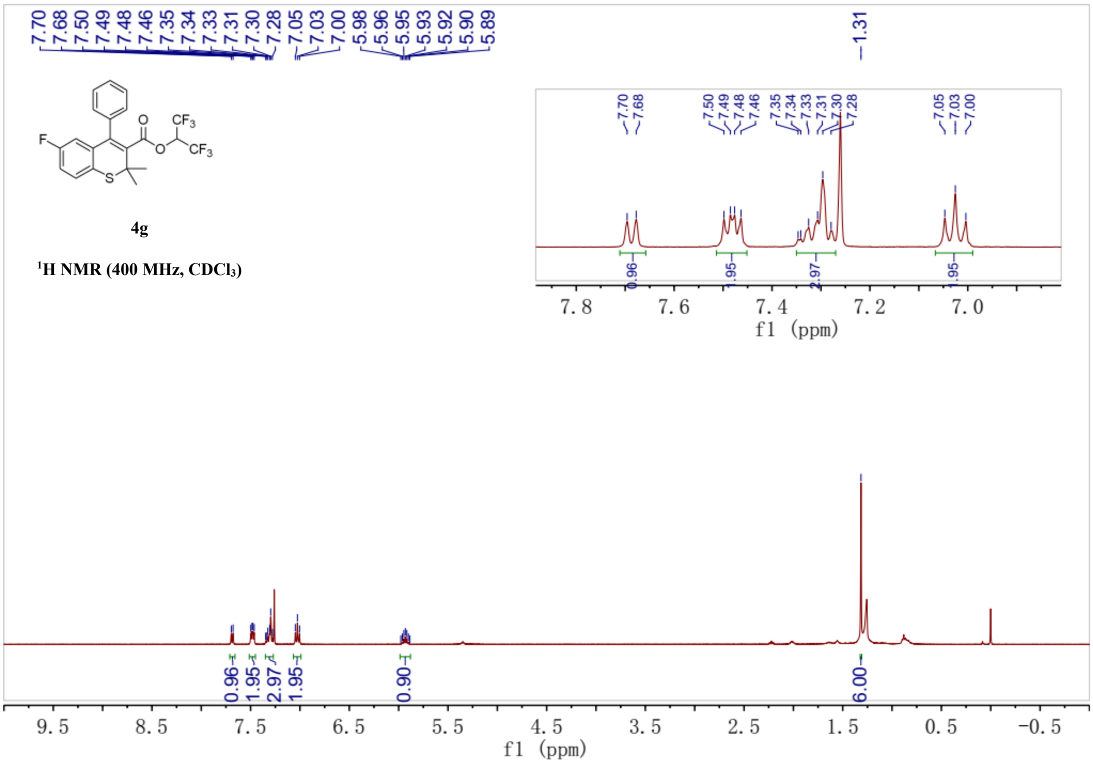
4g
^{1}H NMR (400 MHz, $CDCl_3$)
f1 (ppm)

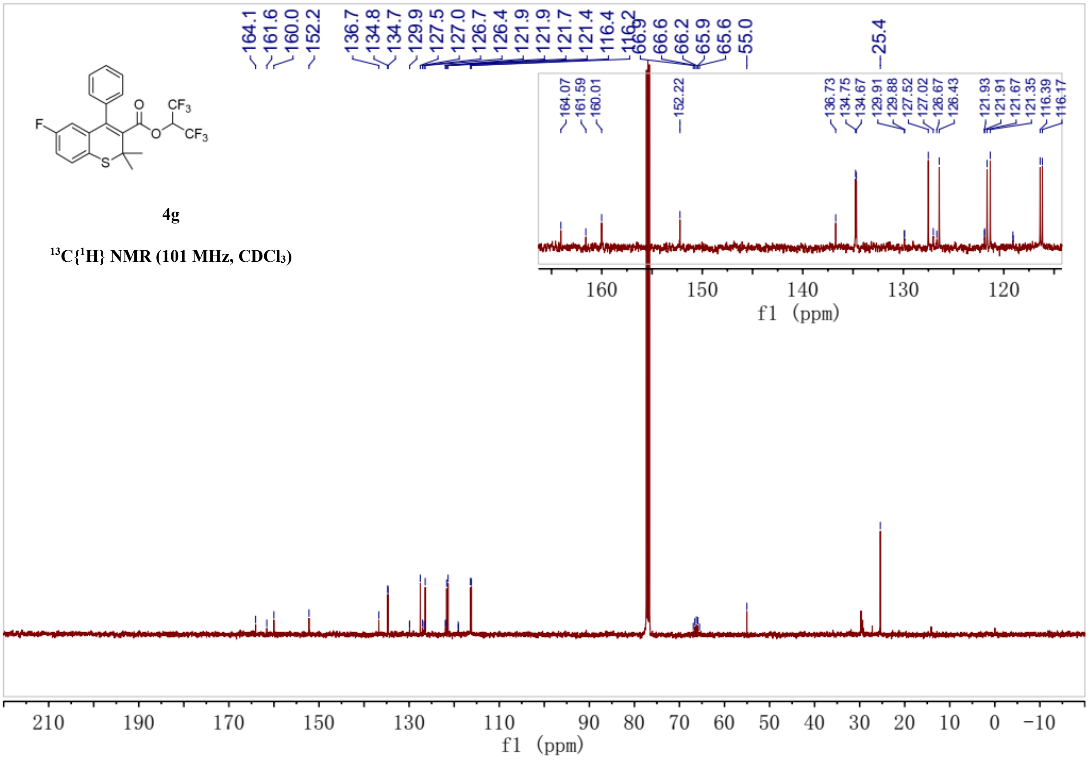
4g
$^{13}C\{^{1}H\}$ NMR (101 MHz, $CDCl_3$)
f1 (ppm)

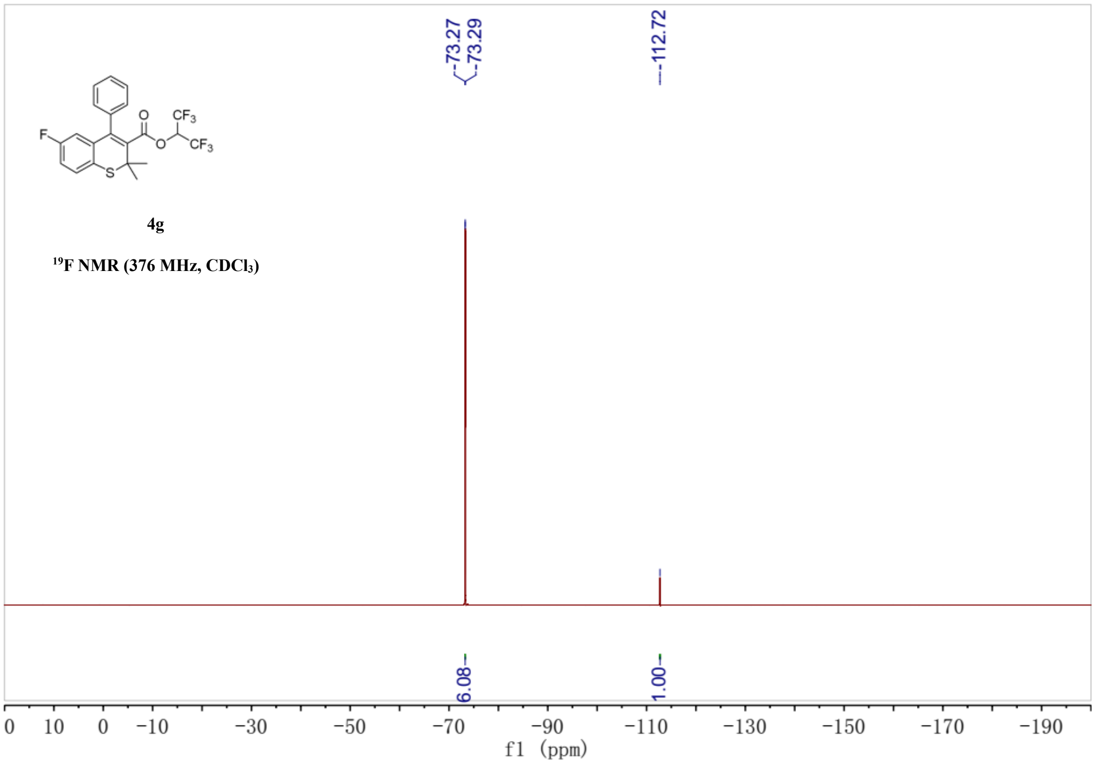

4g

^{19}F NMR (376 MHz, $CDCl_3$)

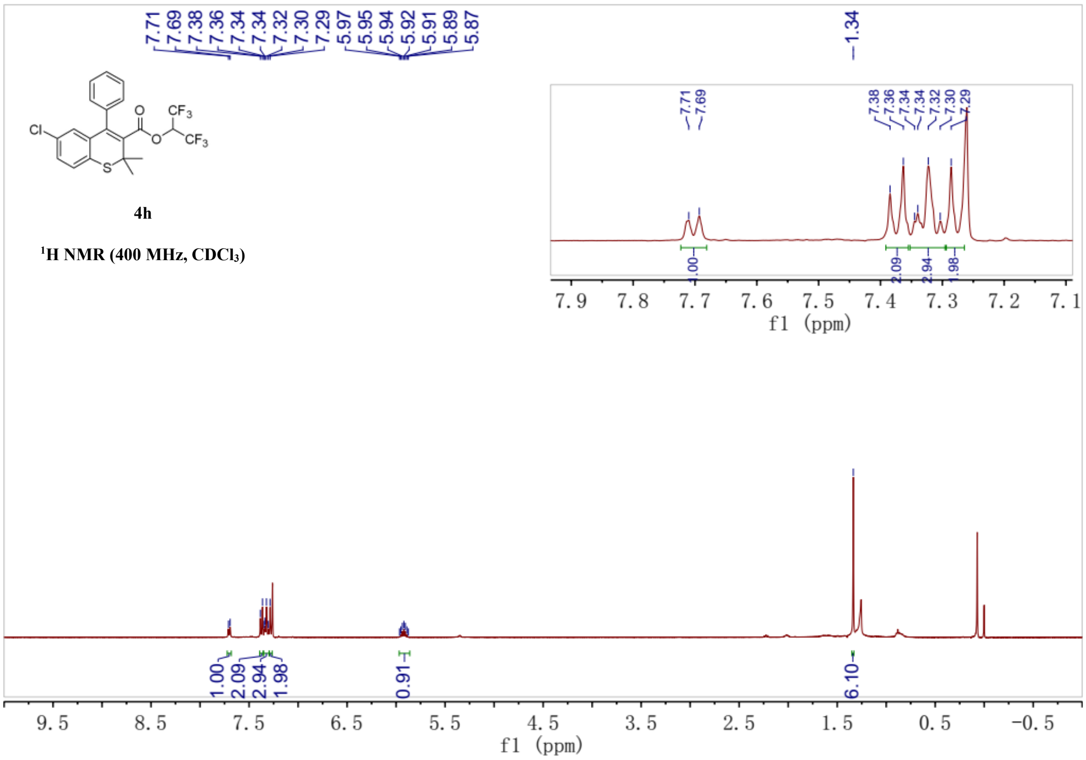
Cl
S
O
O
CF3
CF3
4h
1H NMR (400 MHz, CDCl3)
7.71 7.69 7.38 7.36 7.34 7.34 7.32 7.30 7.29 5.97 5.95 5.94 5.92 5.91 5.89 5.87
1.34
1.00 2.09 2.94 1.98 0.91 6.10
f1 (ppm)

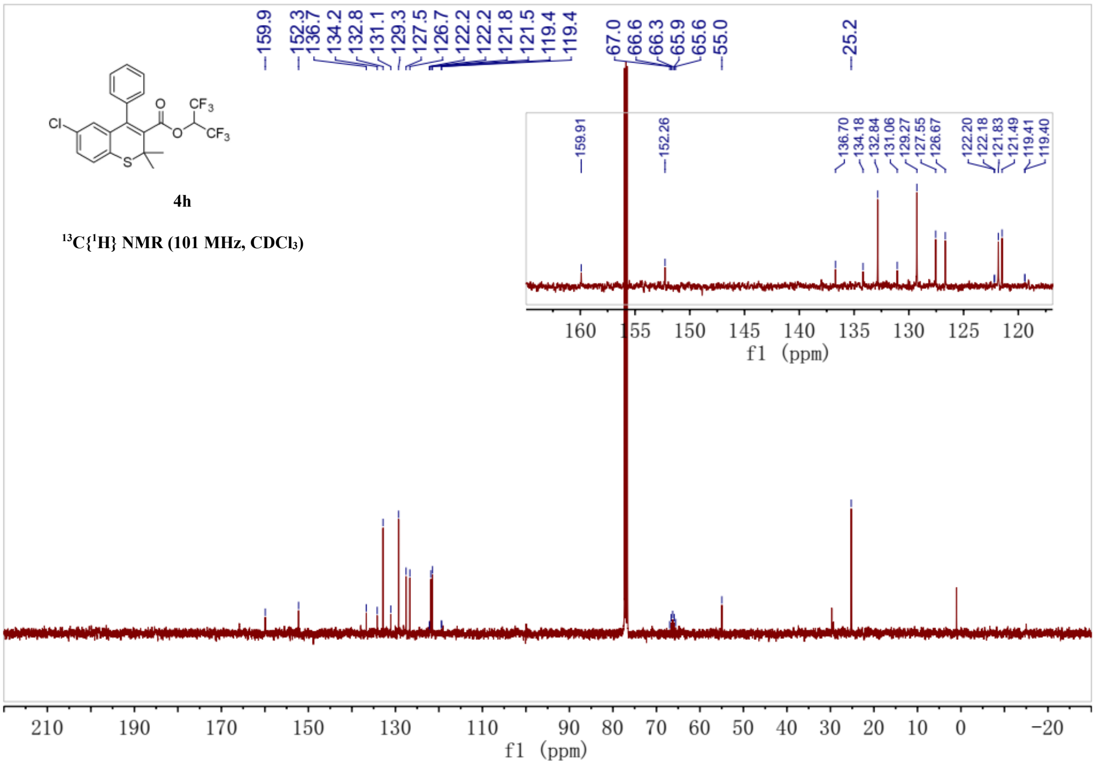
Cl
S
O
O
CF3
CF3
4h
13C{1H} NMR (101 MHz, CDCl3)
159.9 152.3 136.7 134.2 132.8 131.1 129.3 127.5 126.7 122.2 122.2 121.8 121.5 119.4 119.4 67.0 66.6 66.3 65.9 65.6 55.0 25.2
159.91 152.26 136.70 134.18 132.84 131.06 129.27 127.55 126.67 122.20 122.18 121.83 121.49 119.41 119.40
f1 (ppm)

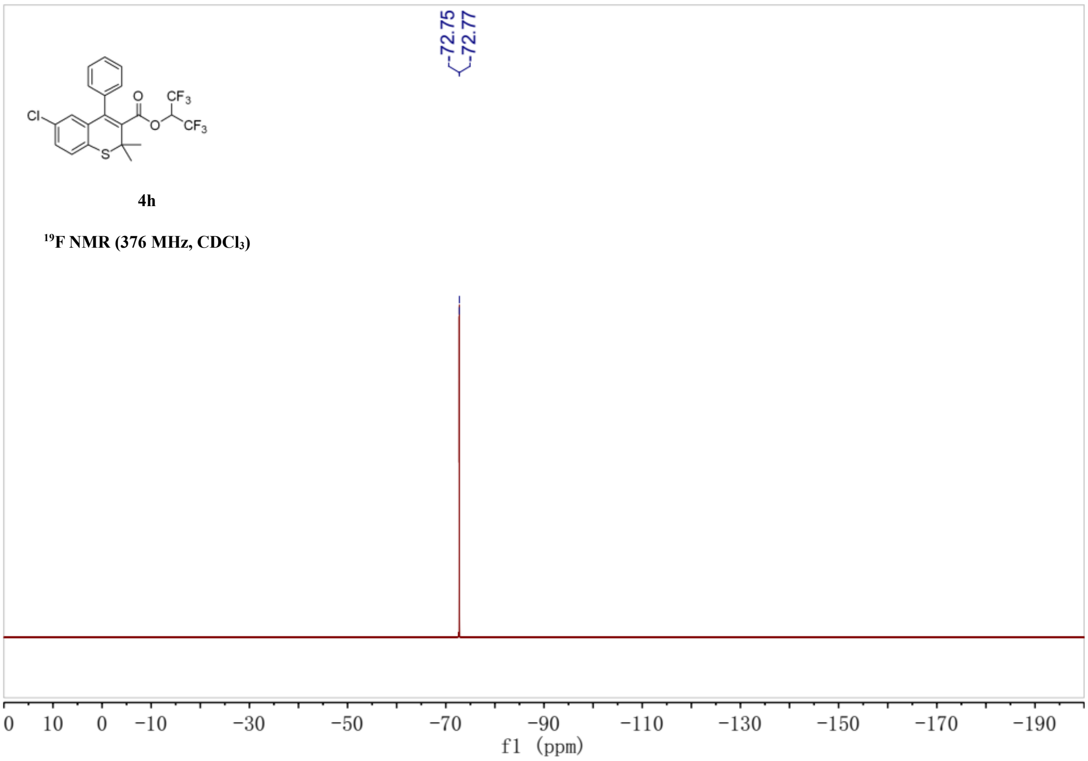
-72.75
-72.77
Cl
O
O
CF3
CF3
S
4h
19F NMR (376 MHz, CDCl3)
0 10 0 −10 −30 −50 −70 −90 −110 −130 −150 −170 −190
f1 (ppm)

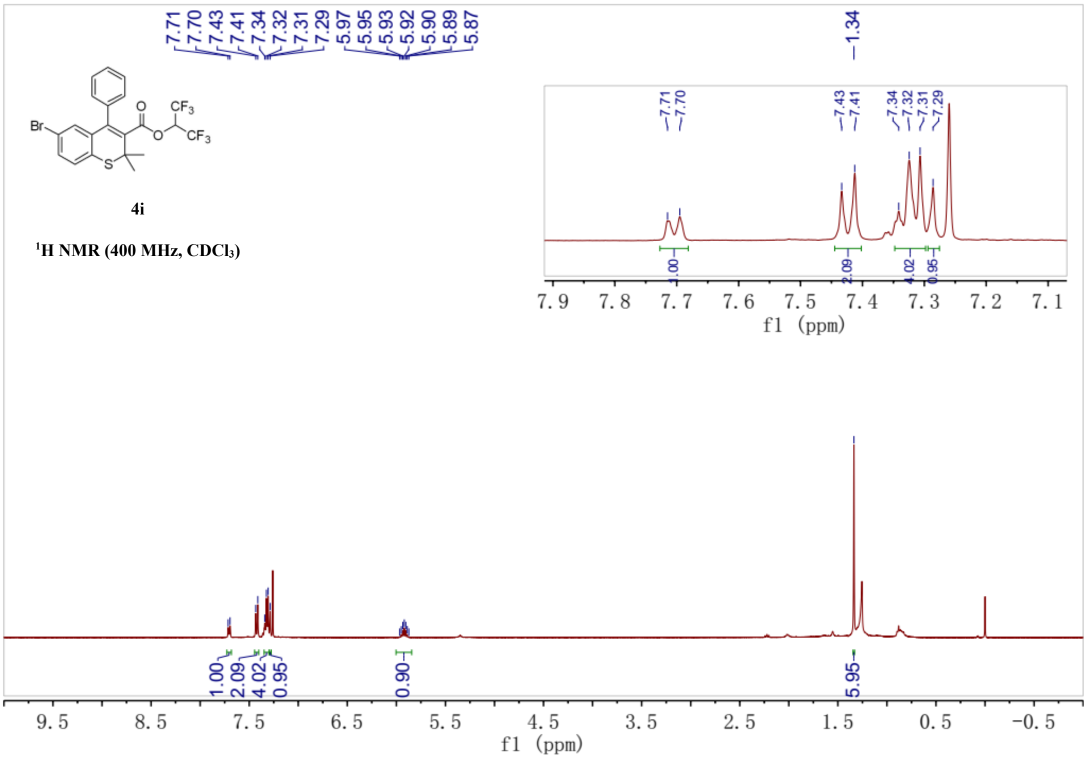

4i
1H NMR (400 MHz, CDCl3)
7.71 7.70 7.43 7.41 7.34 7.32 7.31 7.29 5.97 5.95 5.93 5.92 5.90 5.89 5.87
1.34
1.00 2.09 4.02 0.95 0.90 5.95
f1 (ppm)

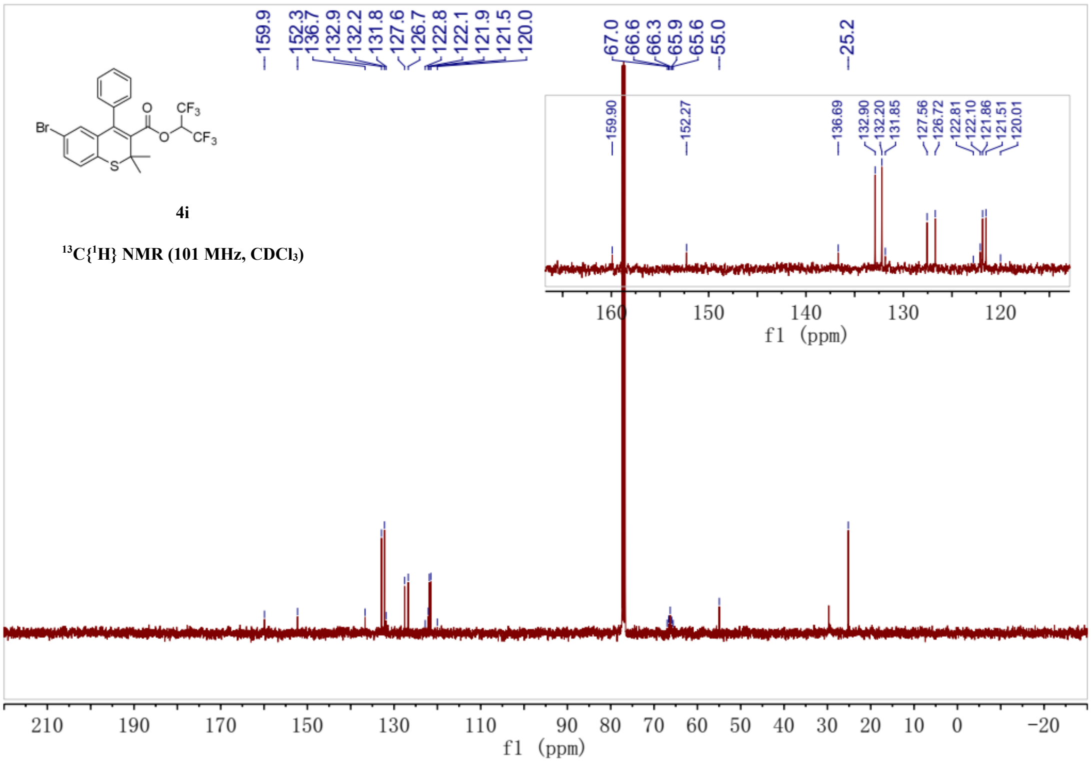

4i
13C{1H} NMR (101 MHz, CDCl3)
159.9 152.3 136.7 132.9 132.2 131.8 127.6 126.7 122.8 122.1 121.9 121.5 120.0
67.0 66.6 66.3 65.9 65.6 55.0
25.2
159.90 152.27 136.69 132.90 132.20 131.85 127.56 126.72 122.81 122.10 121.86 121.51 120.01
f1 (ppm)

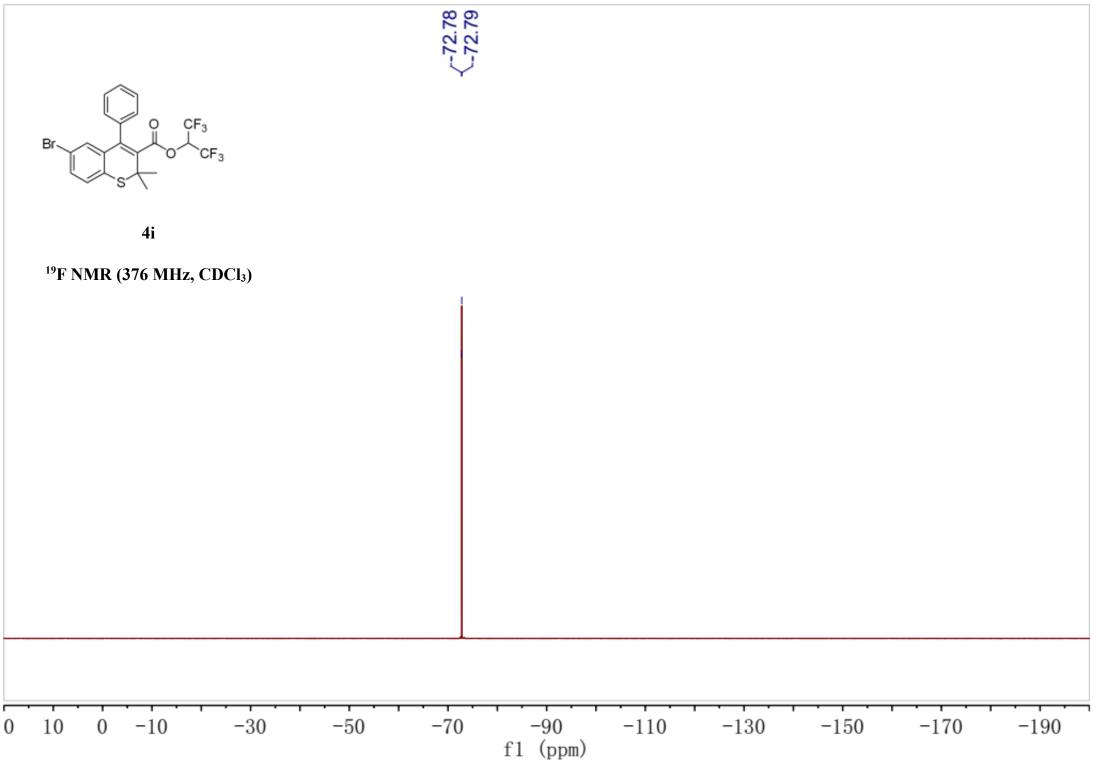
-72.78
-72.79
4i
19F NMR (376 MHz, CDCl3)
0 10 0 −10 −30 −50 −70 −90 −110 −130 −150 −170 −190
f1 (ppm)

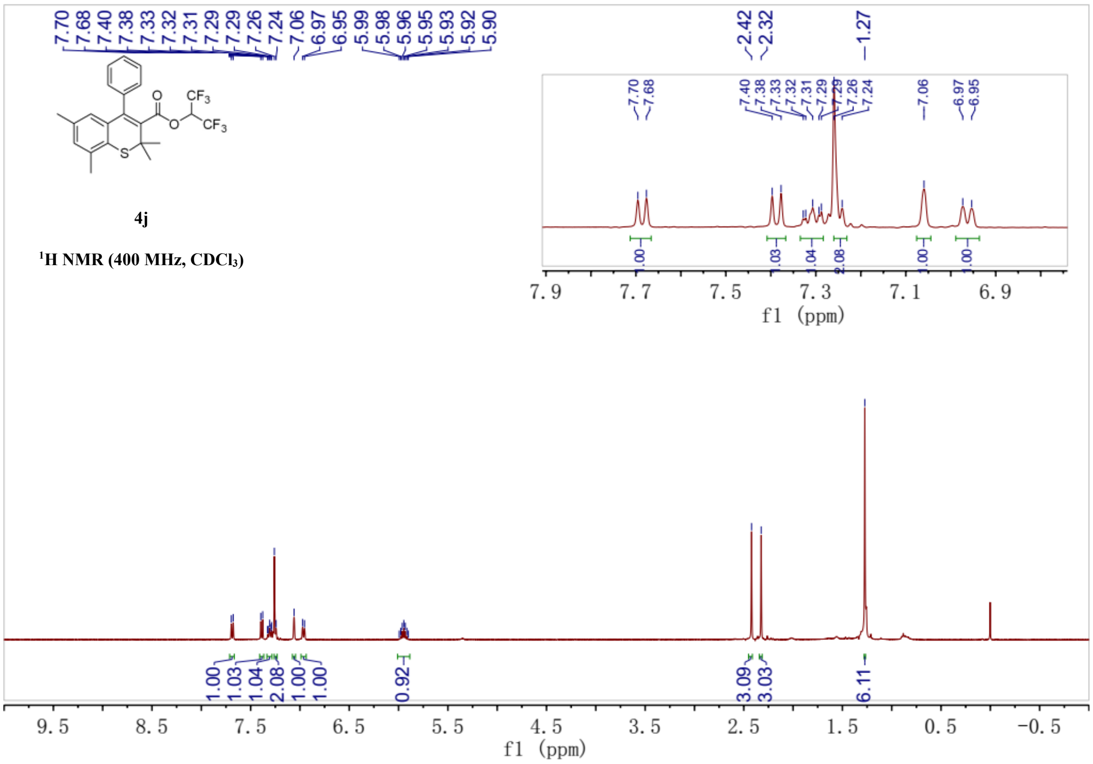
7.70 7.68 7.40 7.38 7.33 7.32 7.31 7.29 7.29 7.26 7.24 7.06 6.97 6.95 5.99 5.98 5.96 5.95 5.93 5.92 5.90
2.42 2.32
1.27
CF3
CF3
O
O
S
4j
1H NMR (400 MHz, CDCl3)
1.00 1.03 1.04 2.08 1.00 1.00
0.92
3.09 3.03
6.11
f1 (ppm)

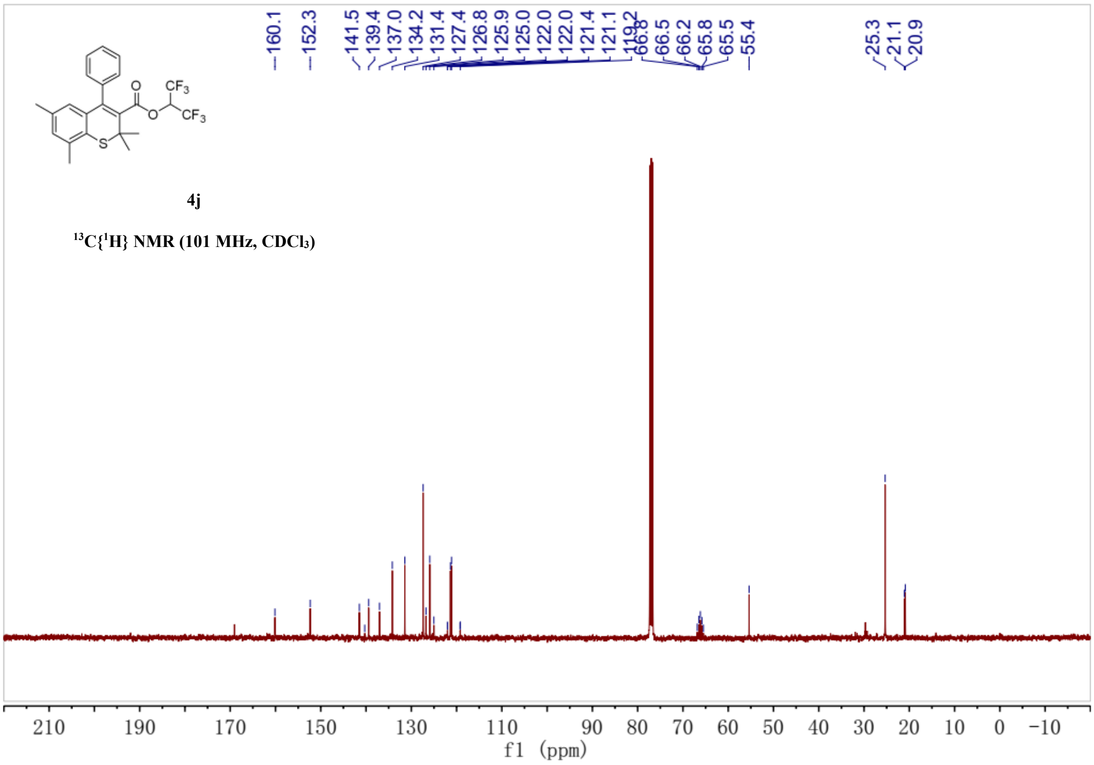
160.1 152.3 141.5 139.4 137.0 134.2 131.4 127.4 126.8 125.9 125.0 122.0 121.4 121.1 119.2 66.8 66.5 66.2 65.8 65.5 55.4
25.3 21.1 20.9
CF3
CF3
O
O
S
4j
13C{1H} NMR (101 MHz, CDCl3)
f1 (ppm)

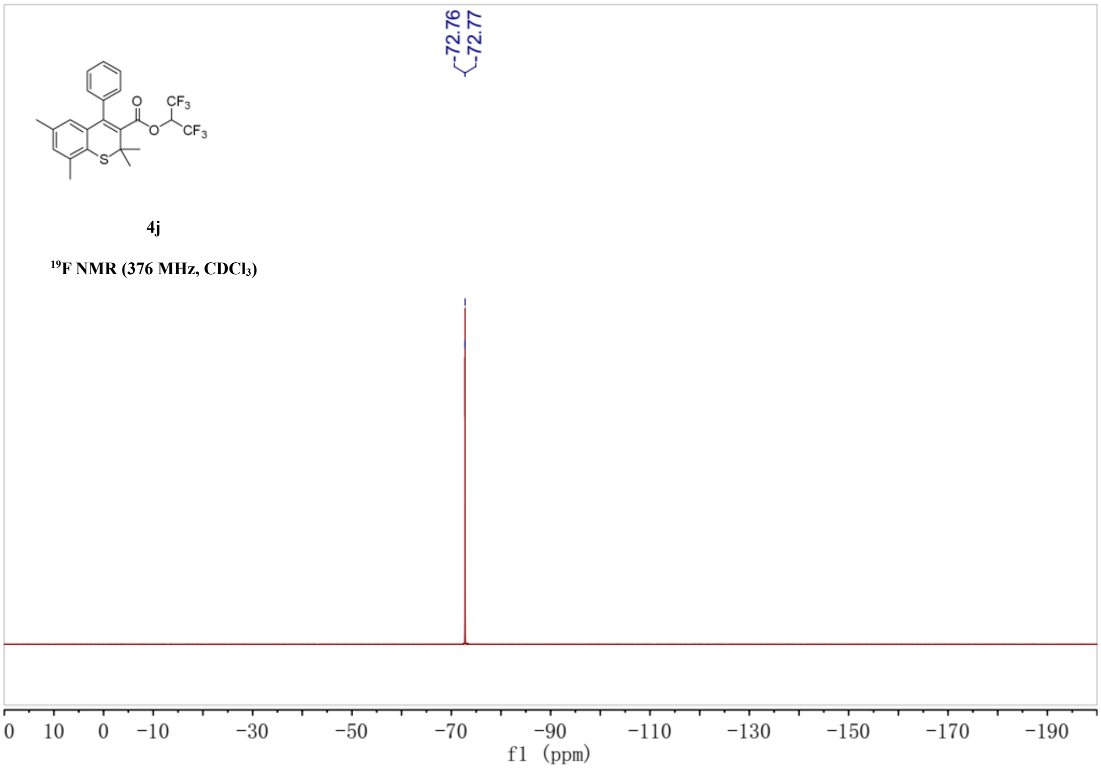

4j

^{19}F NMR (376 MHz, CDCl$_3$)

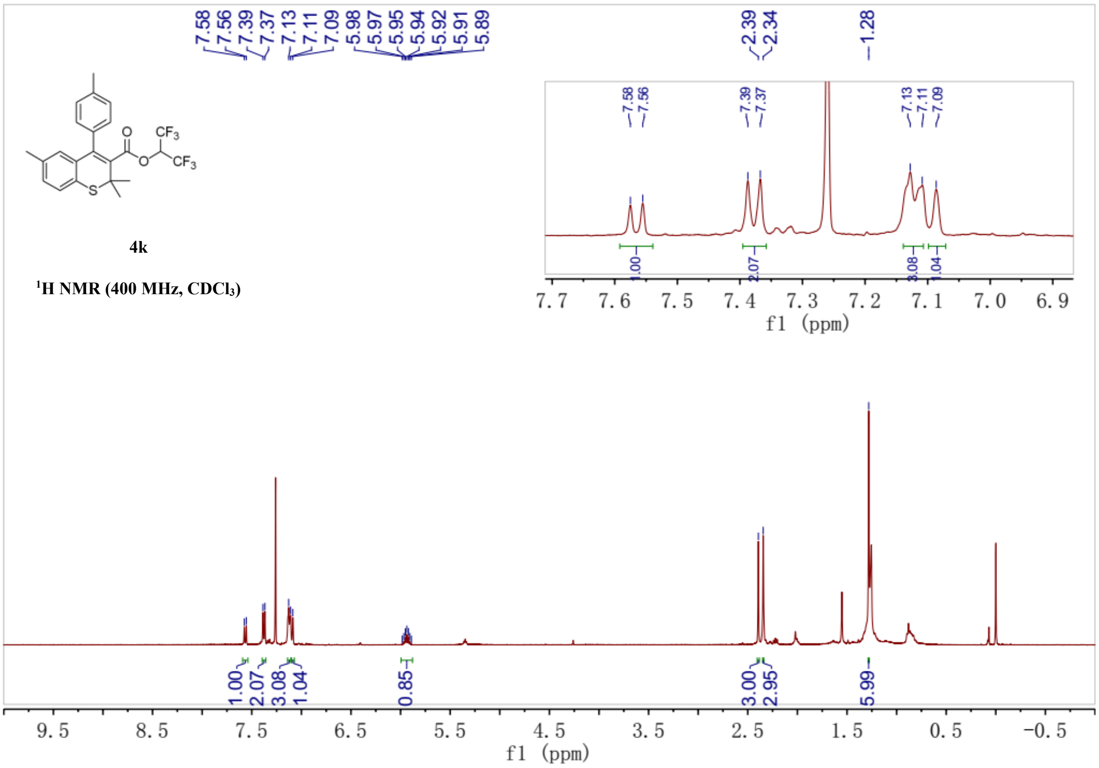
7.58 7.56 7.39 7.37 7.13 7.11 7.09 5.98 5.97 5.95 5.94 5.92 5.91 5.89
2.39 2.34
1.28
CF3
CF3
O
O
S
4k
1H NMR (400 MHz, CDCl3)
1.00 2.07 3.08 1.04 0.85 3.00 2.95 5.99
f1 (ppm)

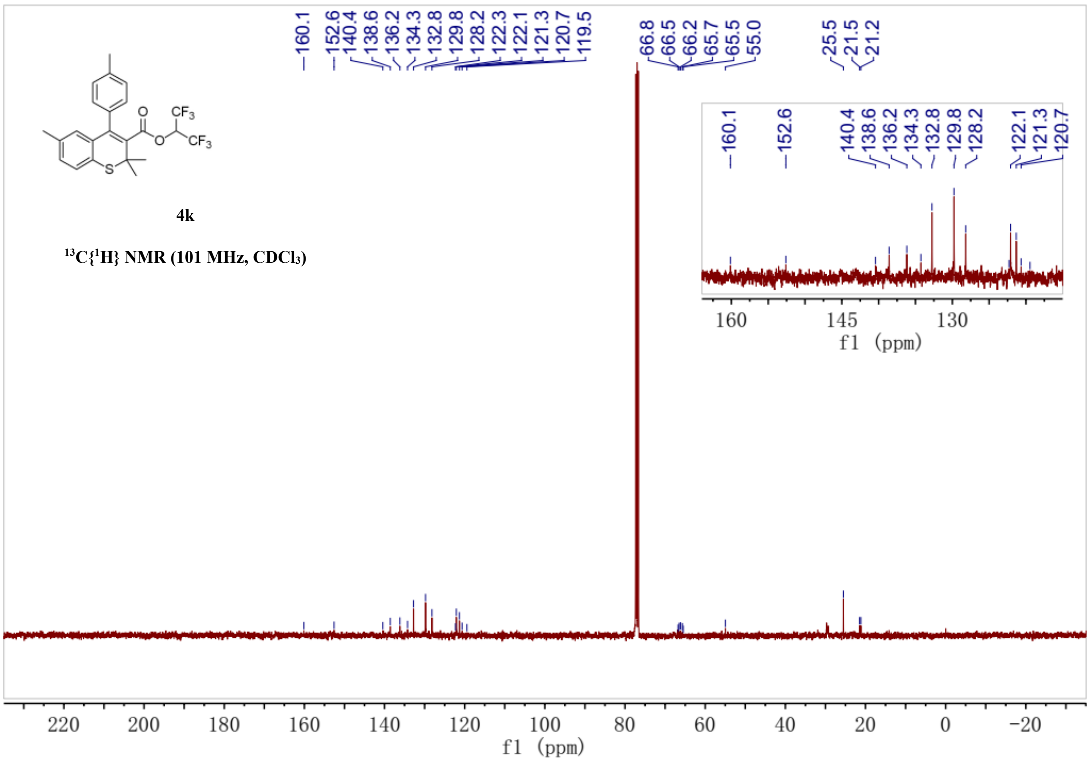
160.1 152.6 140.4 138.6 136.2 134.3 132.8 129.8 128.2 122.3 122.1 121.3 120.7 119.5
66.8 66.5 66.2 65.7 65.5 55.0
25.5 21.5 21.2
CF3
CF3
O
O
S
4k
13C{1H} NMR (101 MHz, CDCl3)
f1 (ppm)

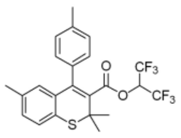

4k

^{19}F NMR (376 MHz, $CDCl_3$)

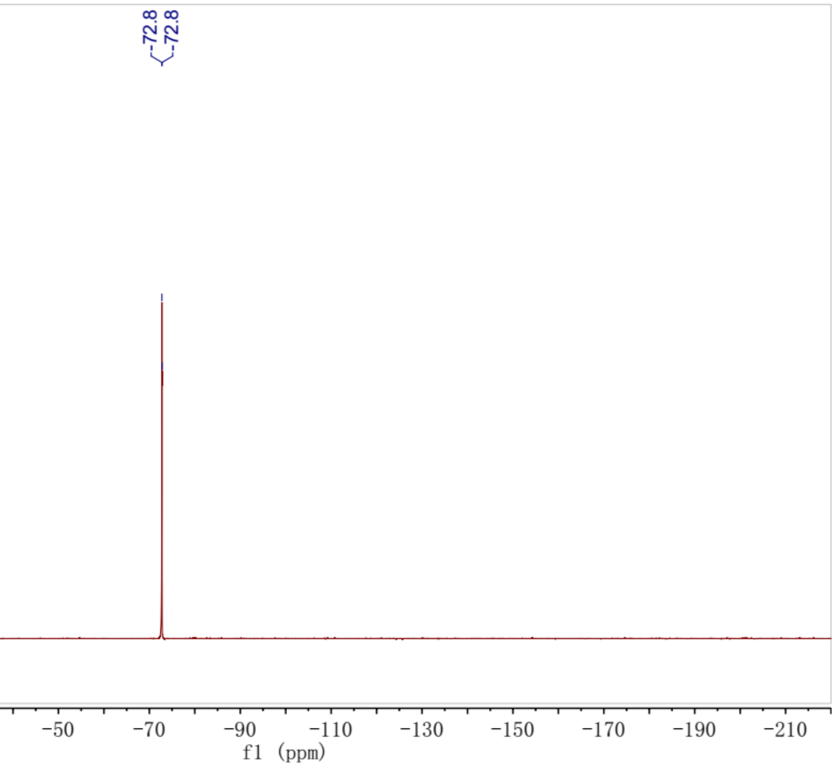

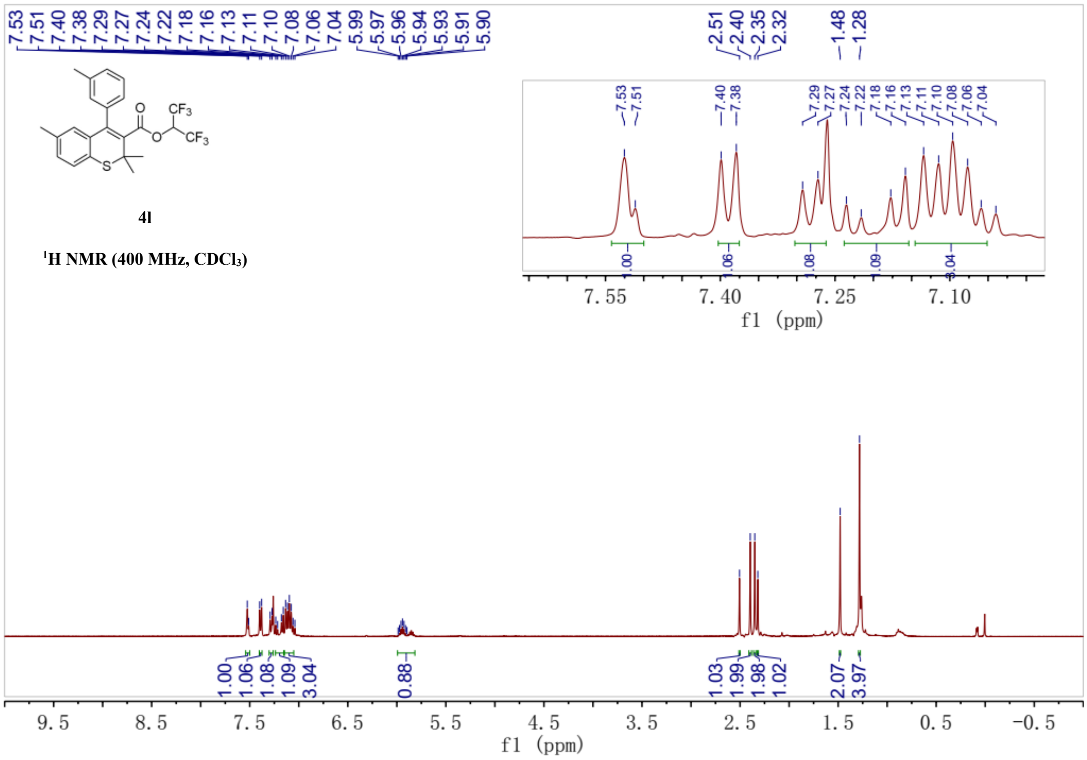

7.53
7.51
7.40
7.38
7.29
7.27
7.24
7.22
7.18
7.16
7.13
7.11
7.10
7.08
7.06
7.04
5.99
5.97
5.96
5.94
5.93
5.91
5.90
2.51
2.40
2.35
2.32
1.48
1.28
4l
^{1}H NMR (400 MHz, CDCl$_3$)
1.00
1.06
1.08
1.09
3.04
0.88
1.03
1.99
1.98
1.02
2.07
3.97
f1 (ppm)

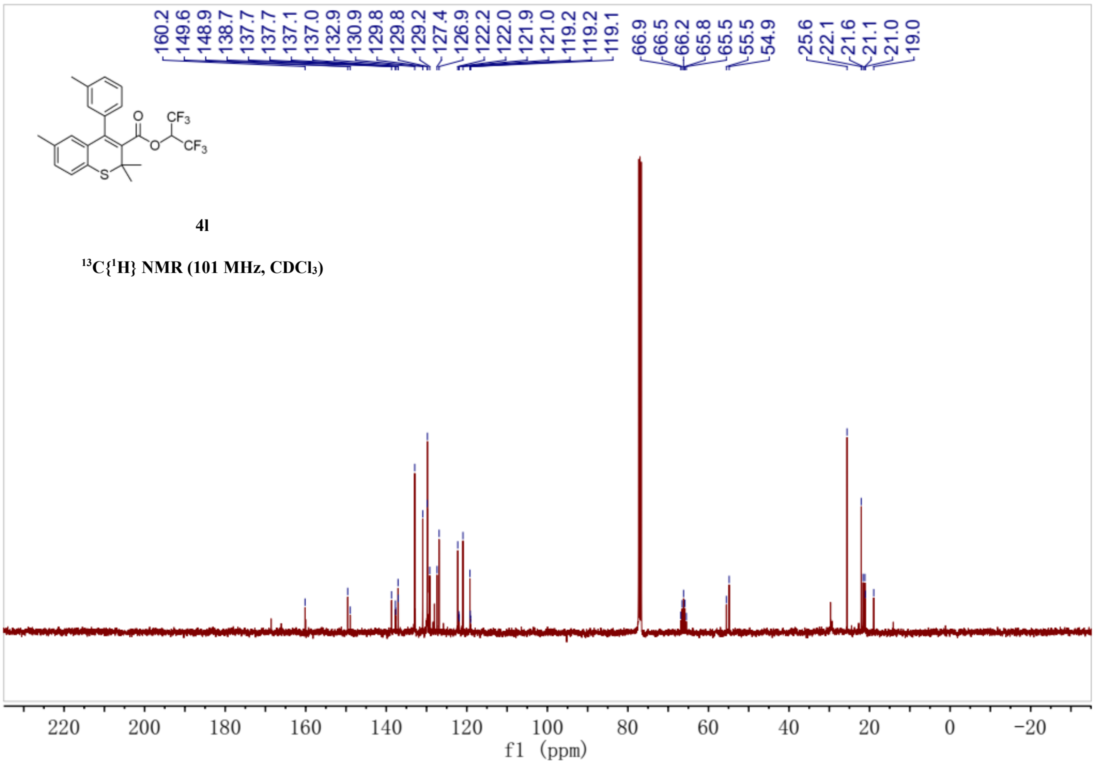

160.2
149.6
148.9
138.7
137.7
137.7
137.1
137.0
132.9
130.9
129.8
129.8
129.2
127.4
126.9
122.2
122.0
121.9
121.0
119.2
119.2
119.1
66.9
66.5
66.2
65.8
65.5
55.5
54.9
25.6
22.1
21.6
21.1
21.0
19.0
4l
^{13}C{^{1}H} NMR (101 MHz, CDCl$_3$)
f1 (ppm)

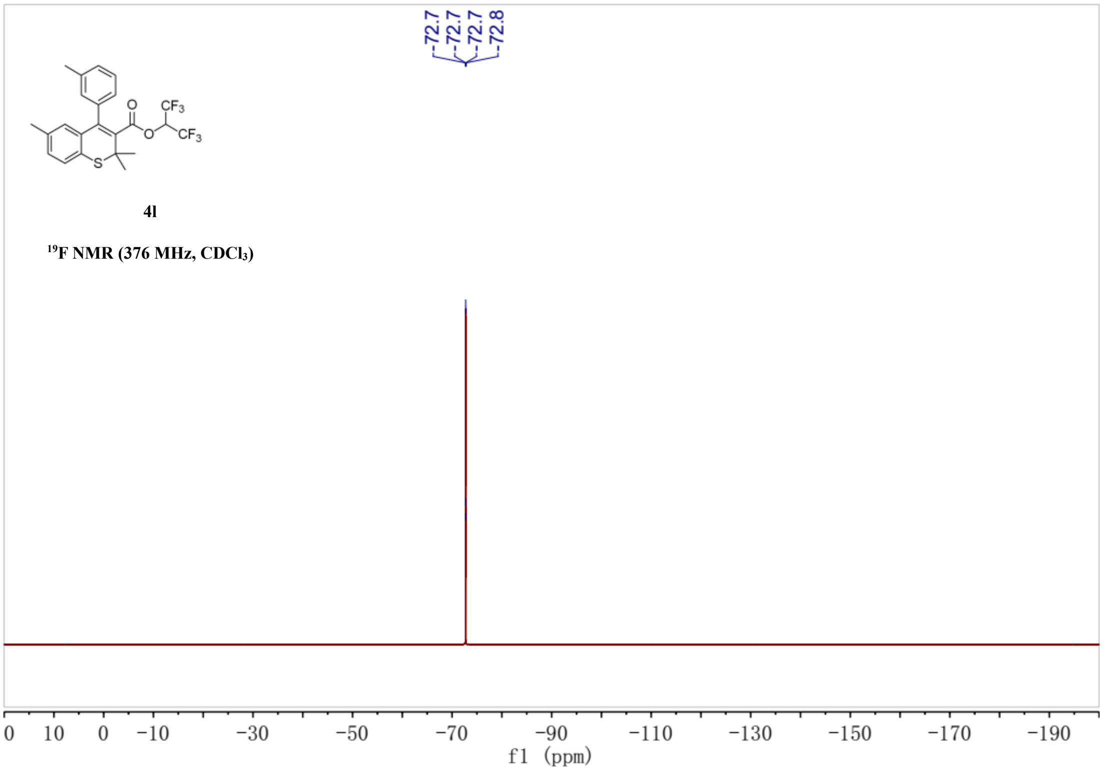
-72.7
-72.7
-72.7
-72.8
CF3
O
O
CF3
S
4l
19F NMR (376 MHz, CDCl3)
0 10 0 −10 −30 −50 −70 −90 −110 −130 −150 −170 −190
f1 (ppm)

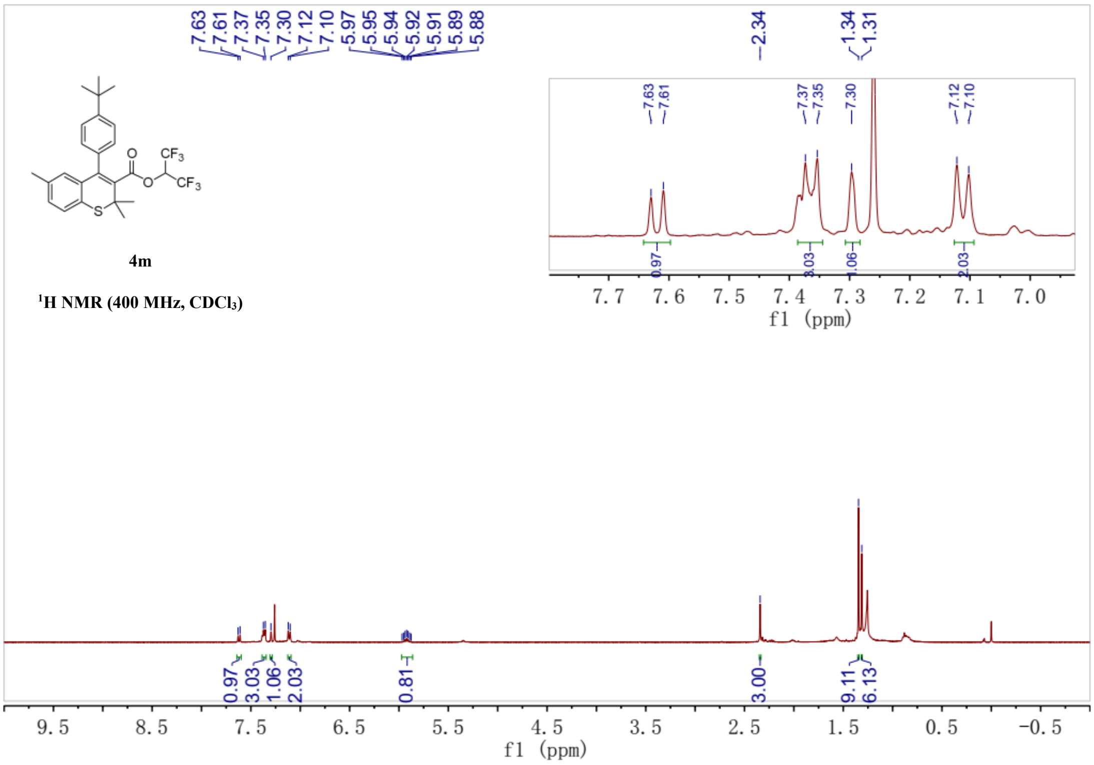
7.63
7.61
7.37
7.35
7.30
7.12
7.10
5.97
5.95
5.94
5.92
5.91
5.89
5.88
2.34
1.34
1.31
CF3
CF3
O
O
S
4m
1H NMR (400 MHz, CDCl3)
0.97
3.03
1.06
2.03
0.81
3.00
9.11
6.13
f1 (ppm)

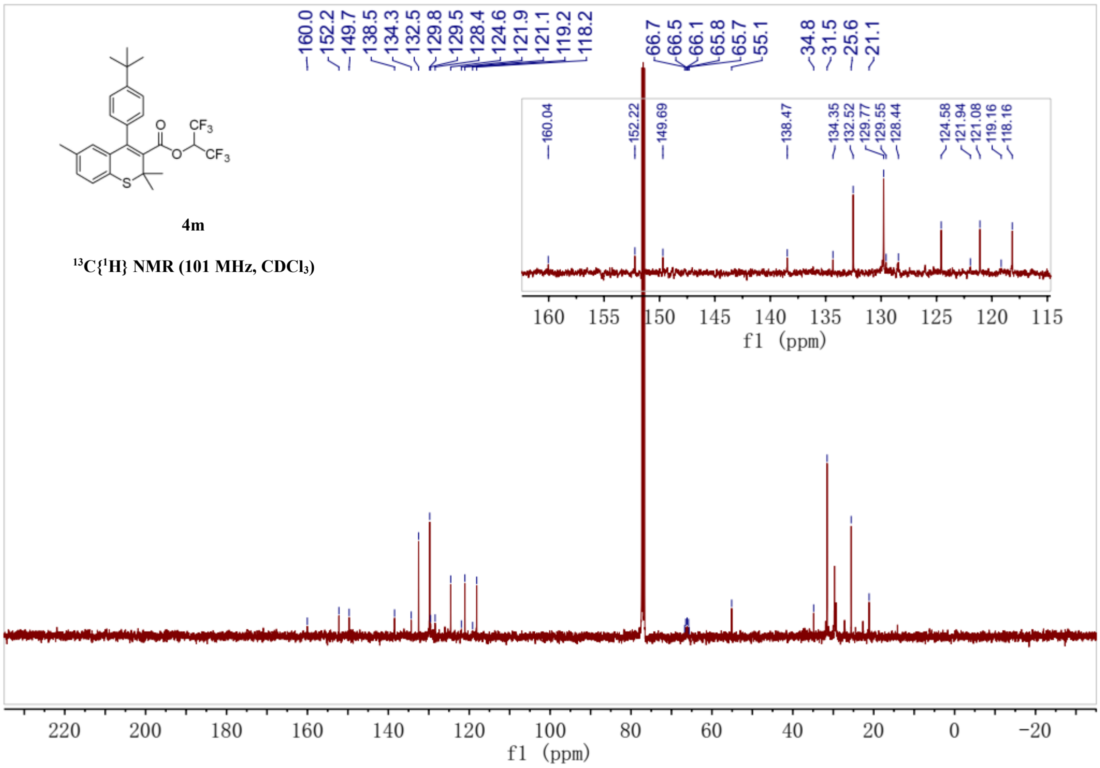
160.0
152.2
149.7
138.5
134.3
132.5
129.8
129.5
128.4
124.6
121.9
121.1
119.2
118.2
66.7
66.5
66.1
65.8
65.7
55.1
34.8
31.5
25.6
21.1
160.04
152.22
149.69
138.47
134.35
132.52
129.77
129.55
128.44
124.58
121.94
121.08
119.16
118.16
CF3
CF3
O
O
S
4m
13C{1H} NMR (101 MHz, CDCl3)
f1 (ppm)

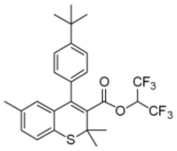

4m

^{19}F NMR (376 MHz, CDCl$_3$)

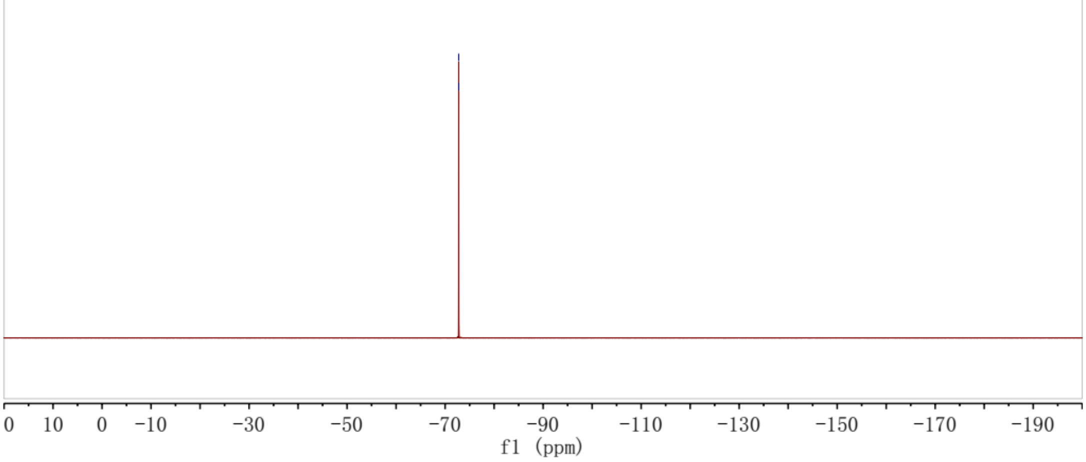

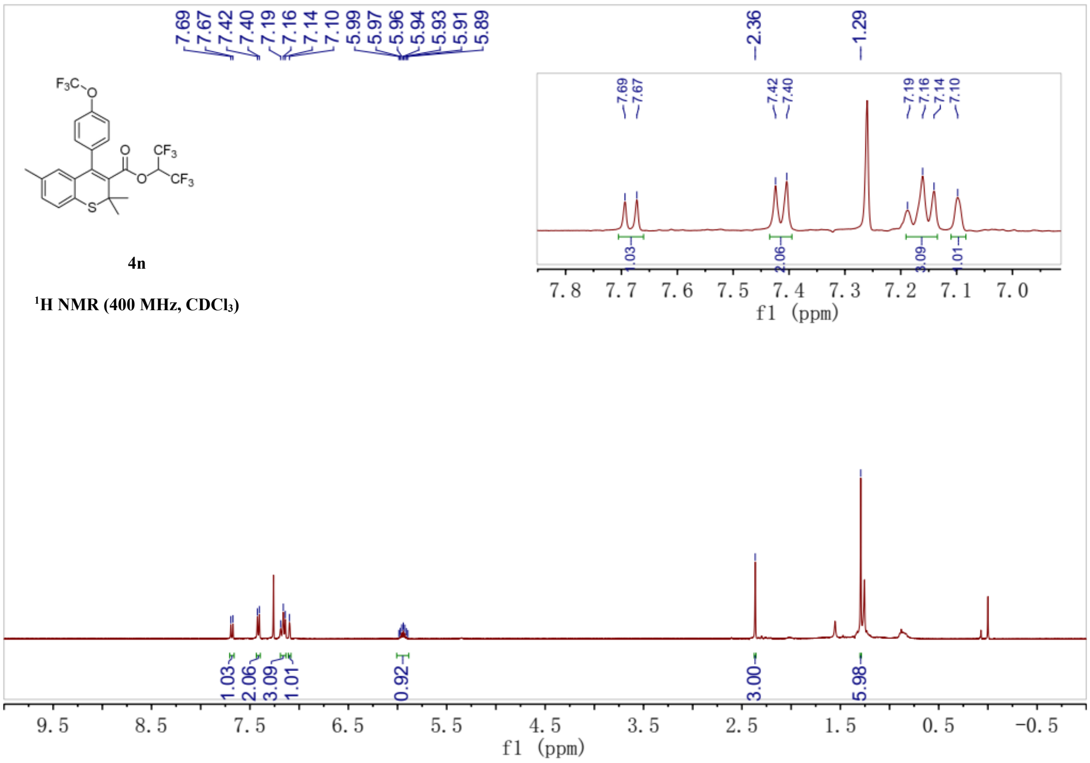

4n

^{1}H NMR (400 MHz, $CDCl_3$)

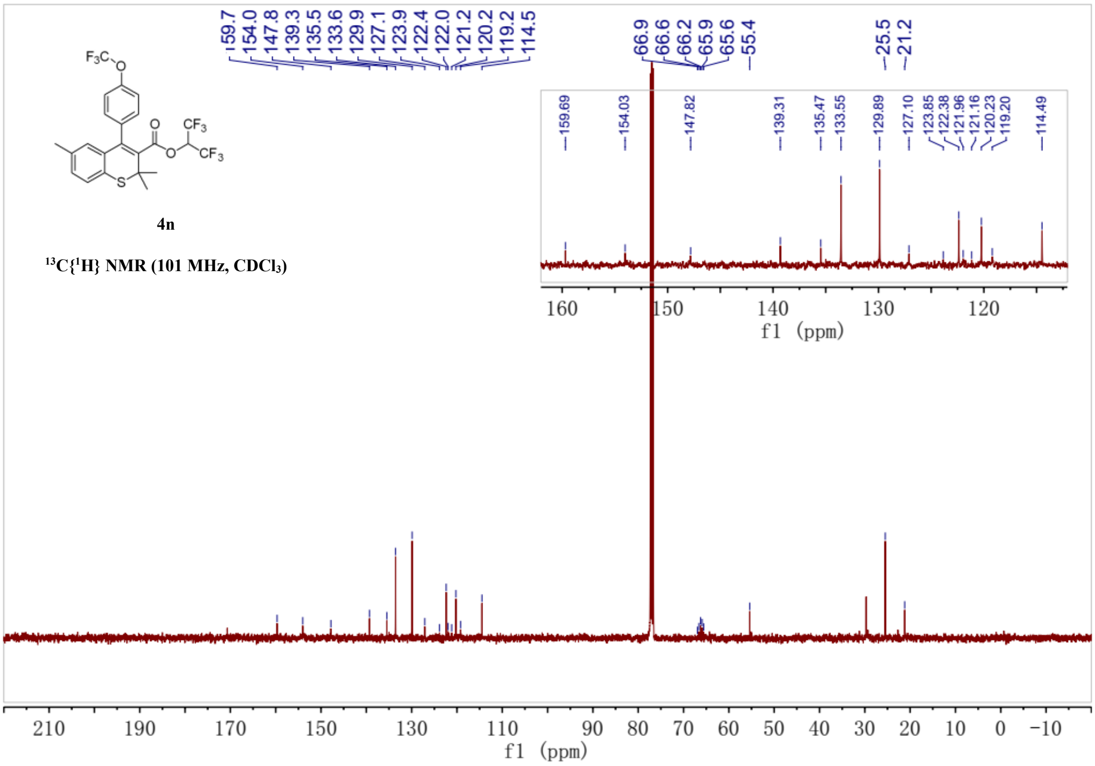

4n

$^{13}C\{^{1}H\}$ NMR (101 MHz, $CDCl_3$)

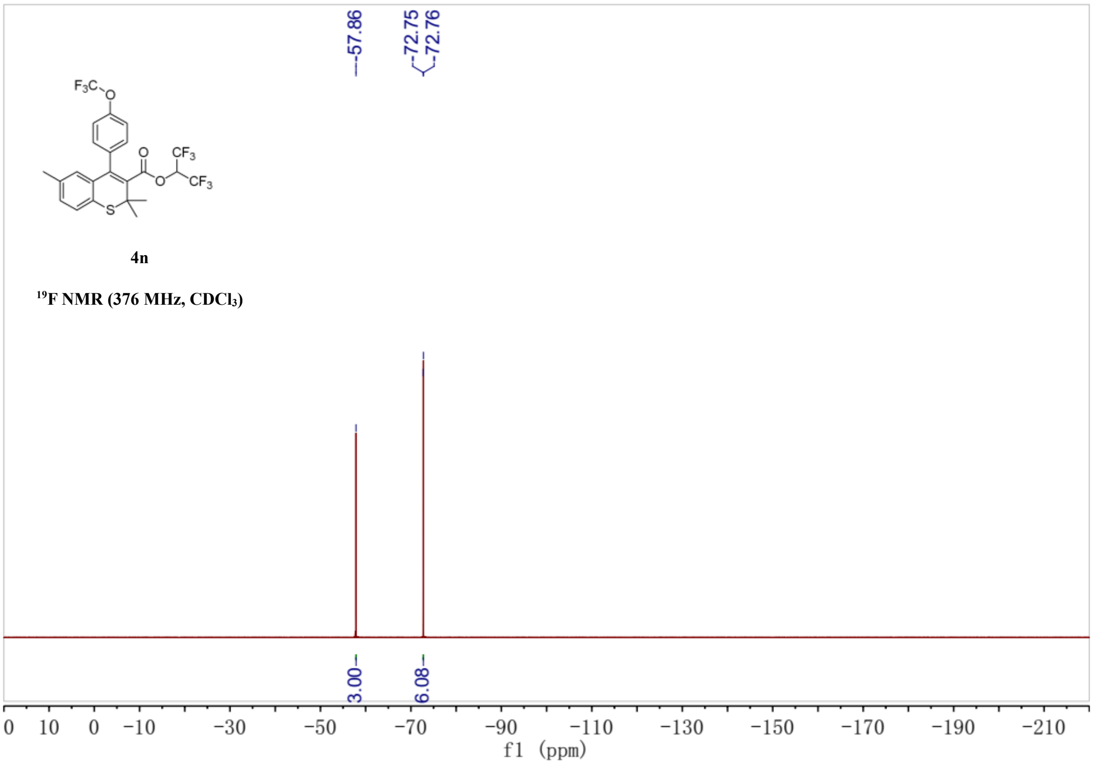
-57.86
-72.75
-72.76
4n
19F NMR (376 MHz, CDCl3)
3.00
6.08
f1 (ppm)

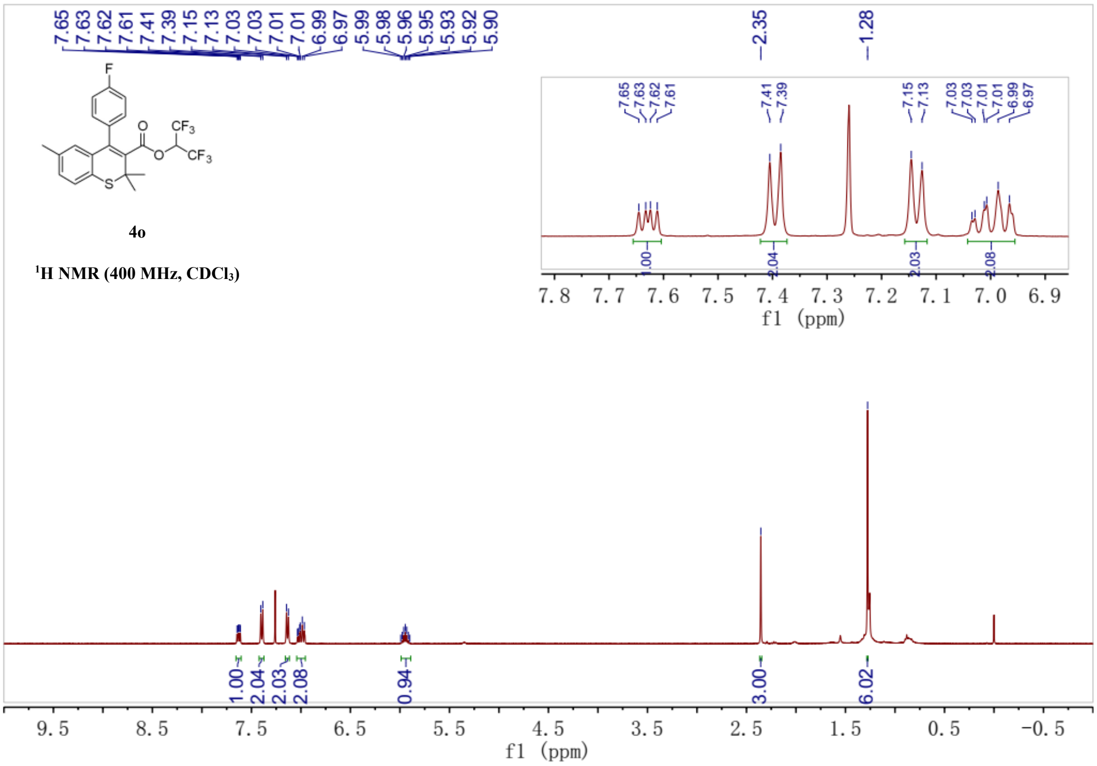
4o
^{1}H NMR (400 MHz, CDCl$_3$)
f1 (ppm)

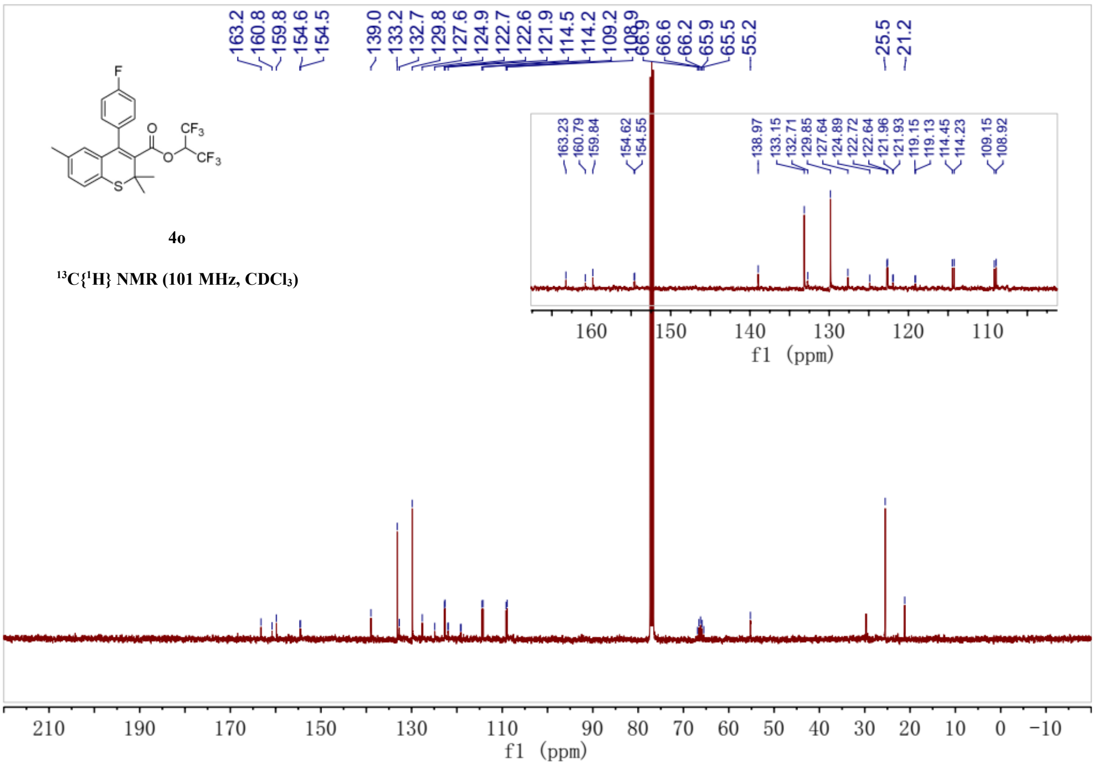
4o
^{13}C{^{1}H} NMR (101 MHz, CDCl$_3$)
f1 (ppm)

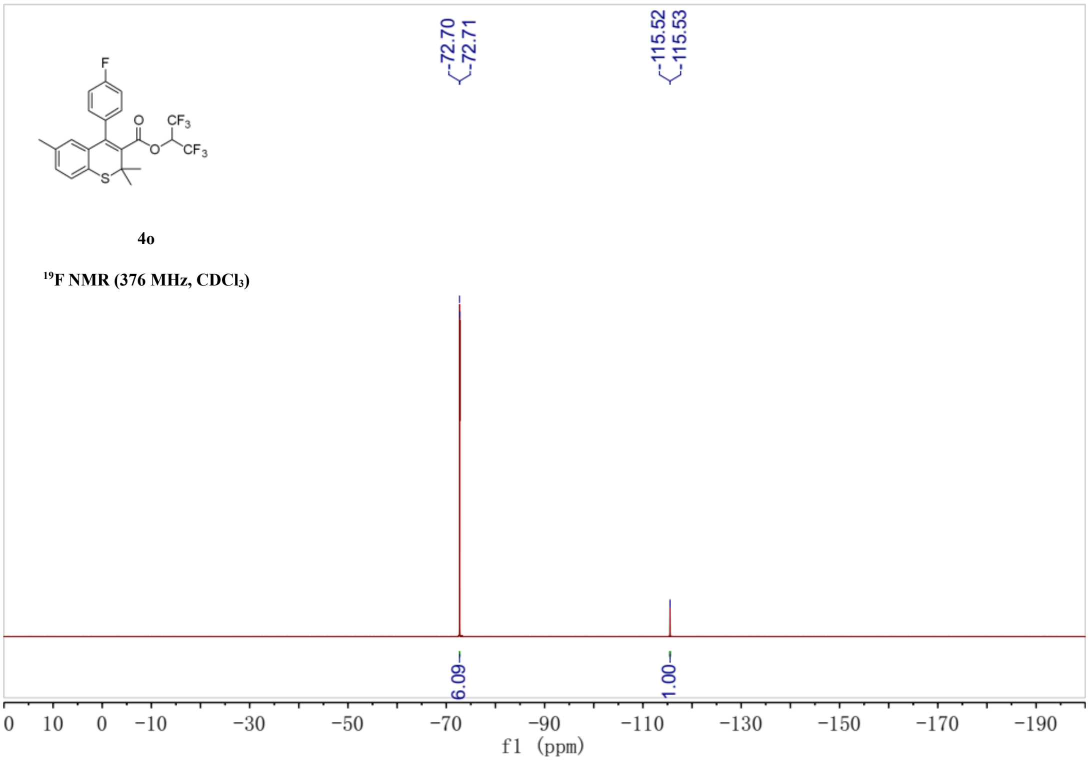

-72.70
-72.71
-115.52
-115.53
F
O
CF3
O
CF3
S
4o
19F NMR (376 MHz, CDCl3)
6.09
1.00
0 10 0 −10 −30 −50 −70 −90 −110 −130 −150 −170 −190
f1 (ppm)

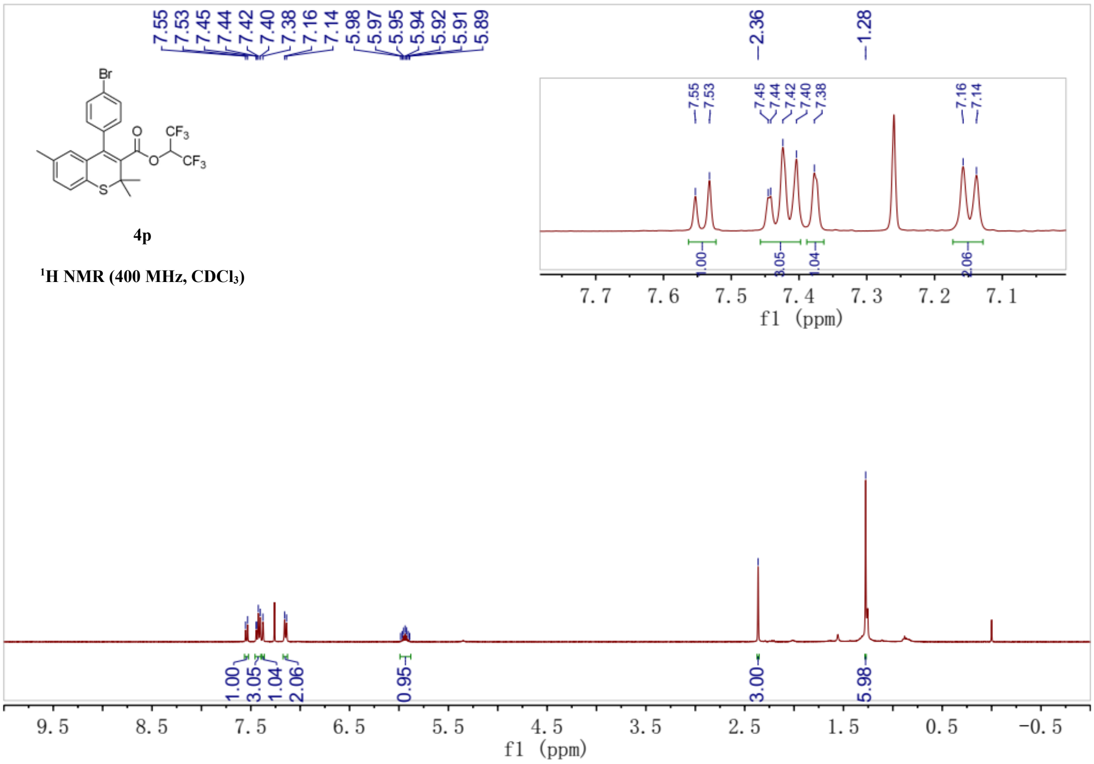

7.55 7.53 7.45 7.44 7.42 7.40 7.38 7.16 7.14 5.98 5.97 5.95 5.94 5.92 5.91 5.89
2.36
1.28
Br
O
CF3
O
CF3
S
4p
1H NMR (400 MHz, CDCl3)
1.00 3.05 1.04 2.06
0.95
3.00
5.98
9.5 8.5 7.5 6.5 5.5 4.5 3.5 2.5 1.5 0.5 -0.5
f1 (ppm)
7.7 7.6 7.5 7.4 7.3 7.2 7.1
f1 (ppm)

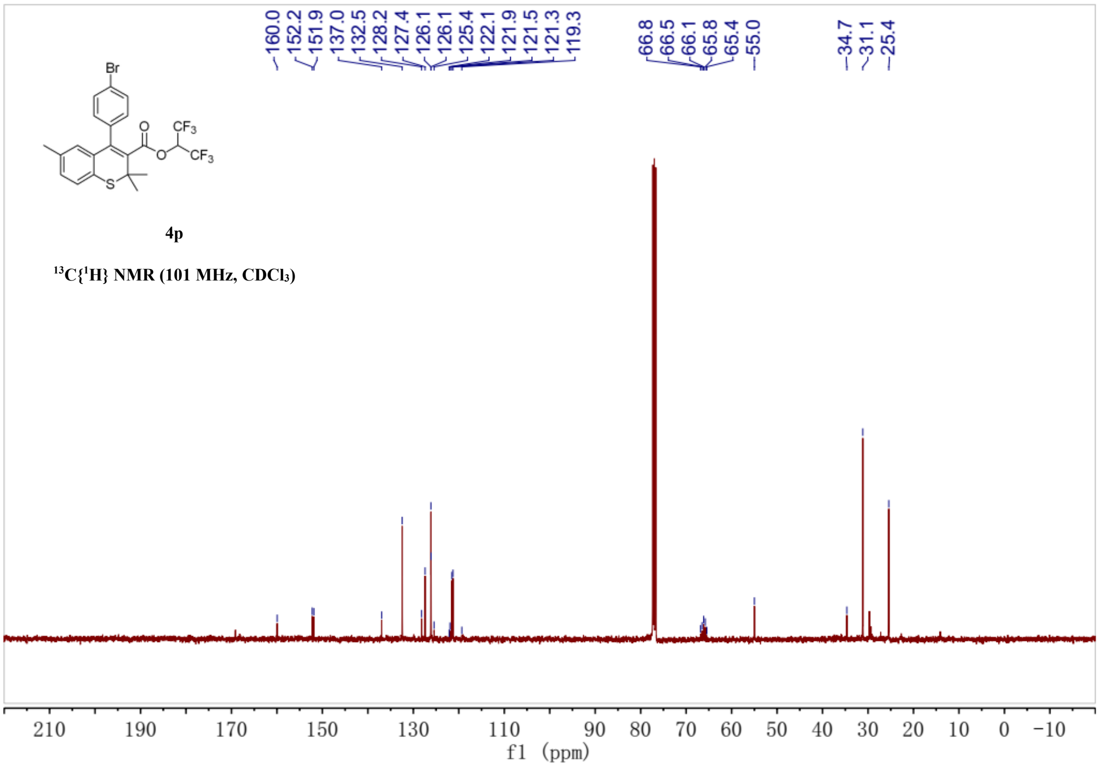

160.0 152.2 151.9 137.0 132.5 128.2 127.4 126.1 126.1 125.4 122.1 121.9 121.5 121.3 119.3
66.8 66.5 66.1 65.8 65.4 55.0
34.7 31.1 25.4
Br
O
CF3
O
CF3
S
4p
13C{1H} NMR (101 MHz, CDCl3)
210 190 170 150 130 110 90 80 70 60 50 40 30 20 10 0 -10
f1 (ppm)

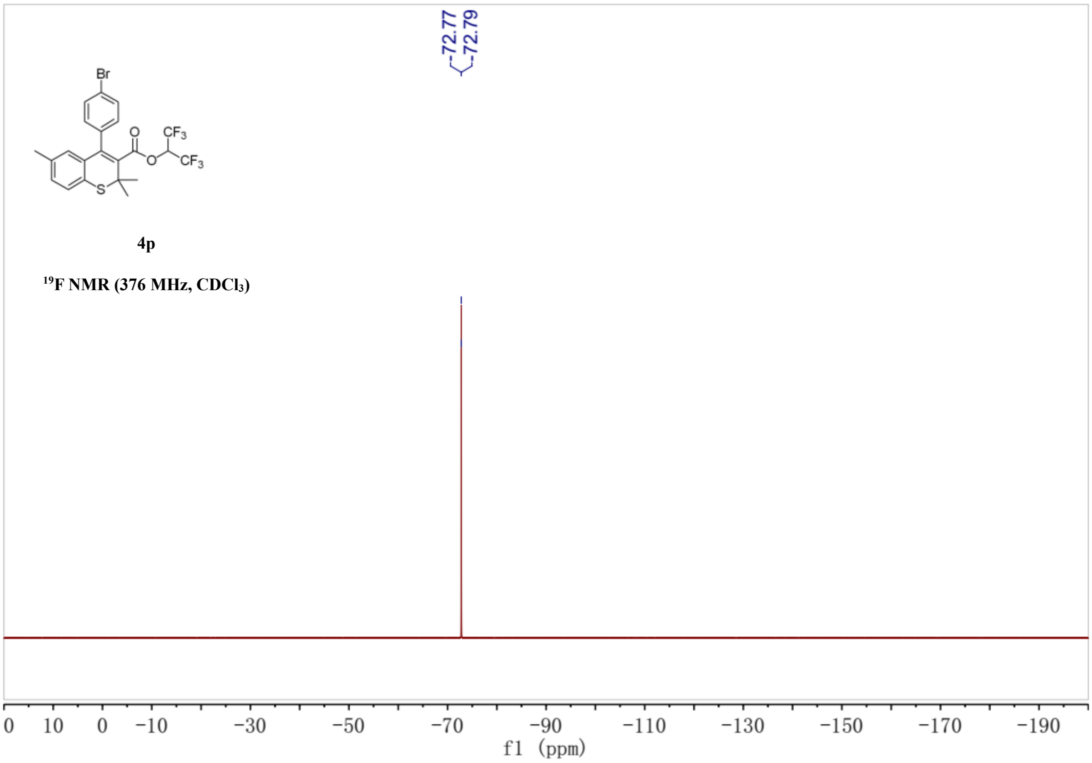
-72.77
-72.79
Br
O
CF3
O
CF3
S
4p
19F NMR (376 MHz, CDCl3)
0 10 0 −10 −30 −50 −70 −90 −110 −130 −150 −170 −190
f1 (ppm)

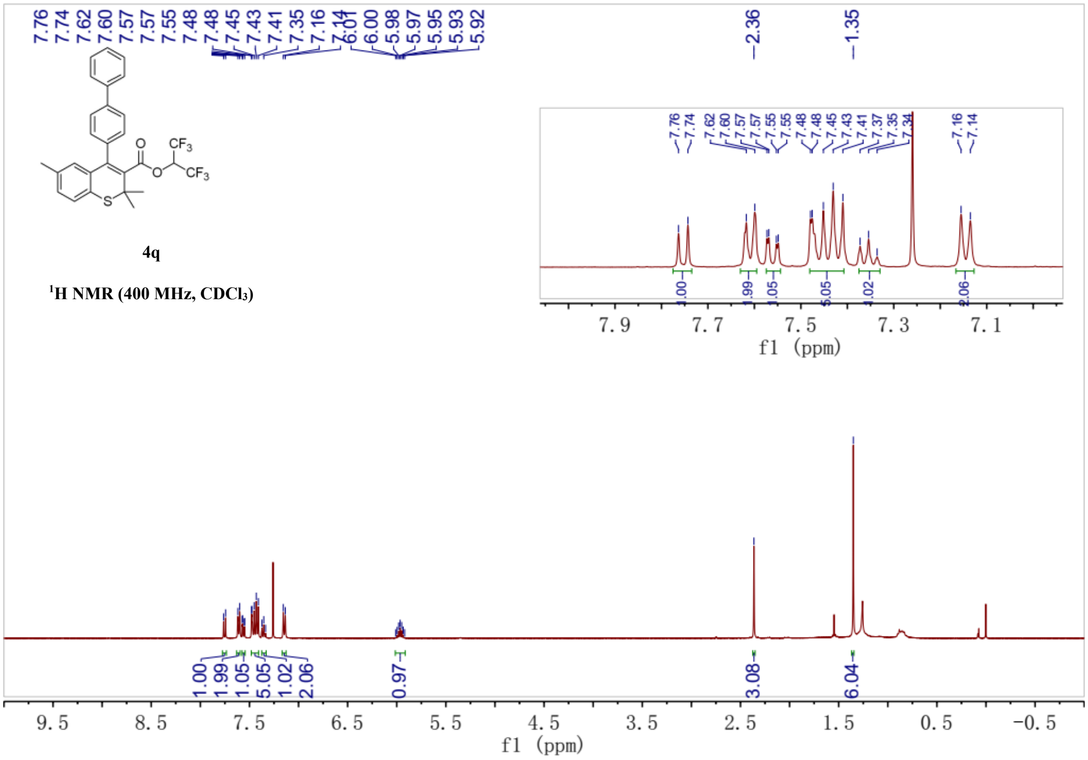

4q

^{1}H NMR (400 MHz, CDCl3)

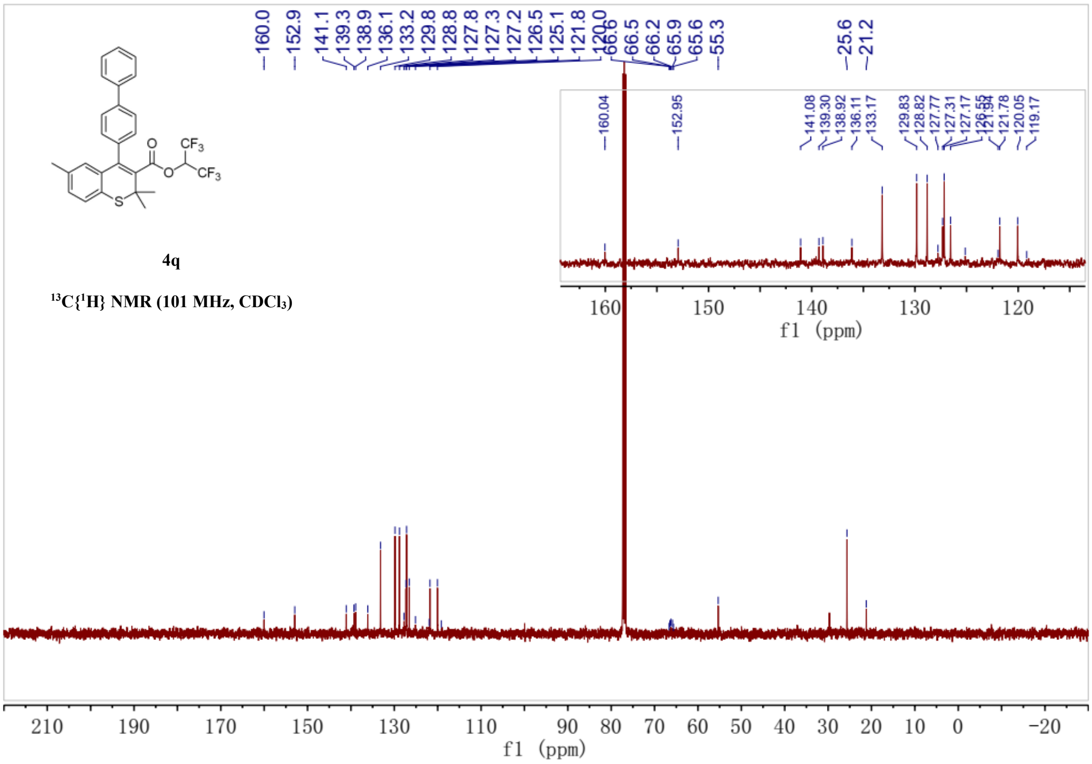

4q

$^{13}C\{^{1}H\}$ NMR (101 MHz, CDCl3)

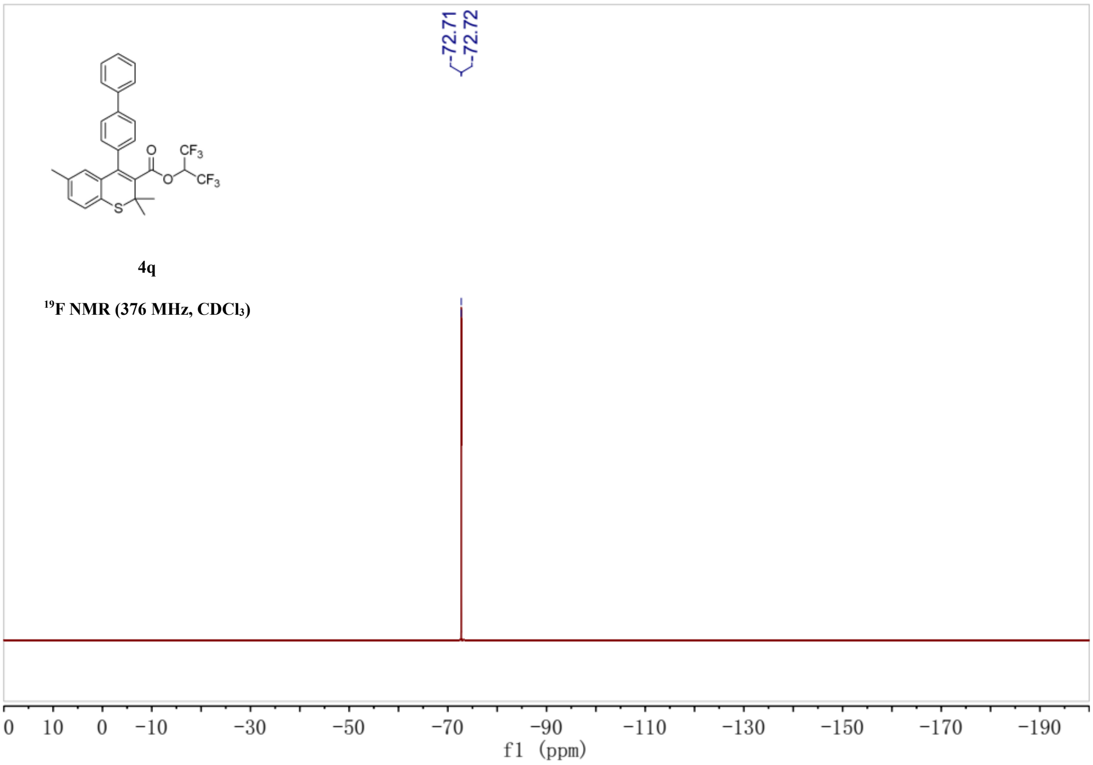
-72.71
-72.72
CF3
O
O
CF3
S
4q
19F NMR (376 MHz, CDCl3)
0 10 0 −10 −30 −50 −70 −90 −110 −130 −150 −170 −190
f1 (ppm)

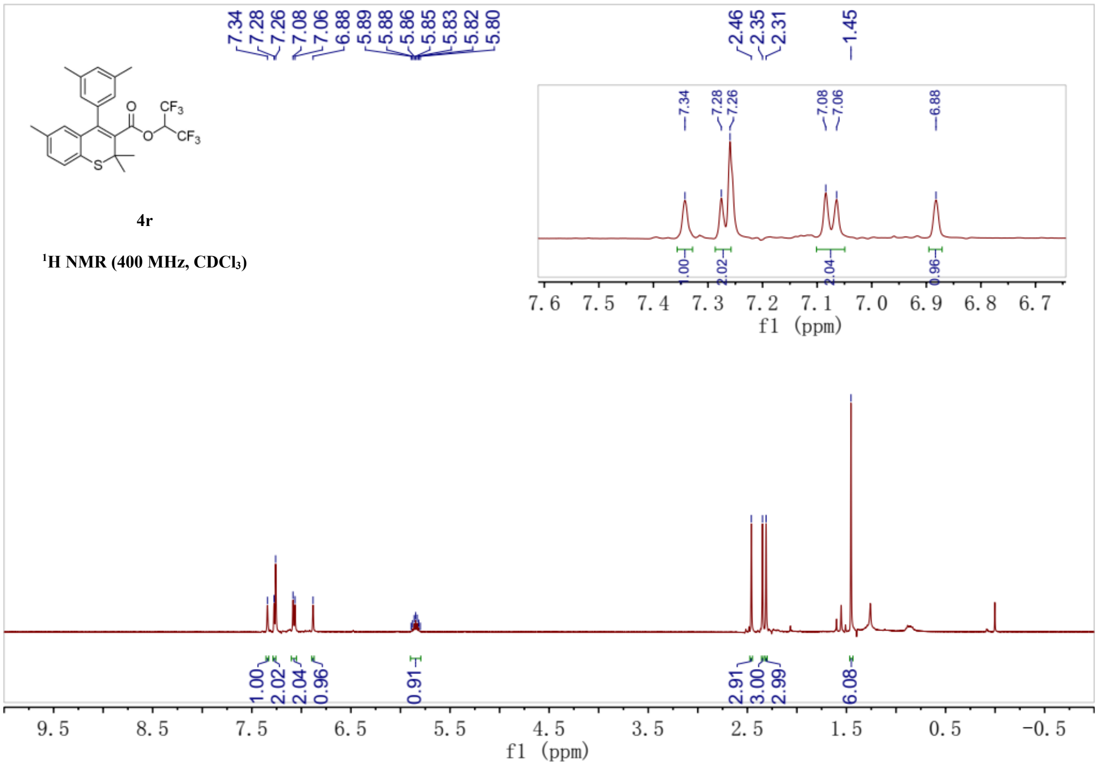

4r

^{1}H NMR (400 MHz, $CDCl_3$)

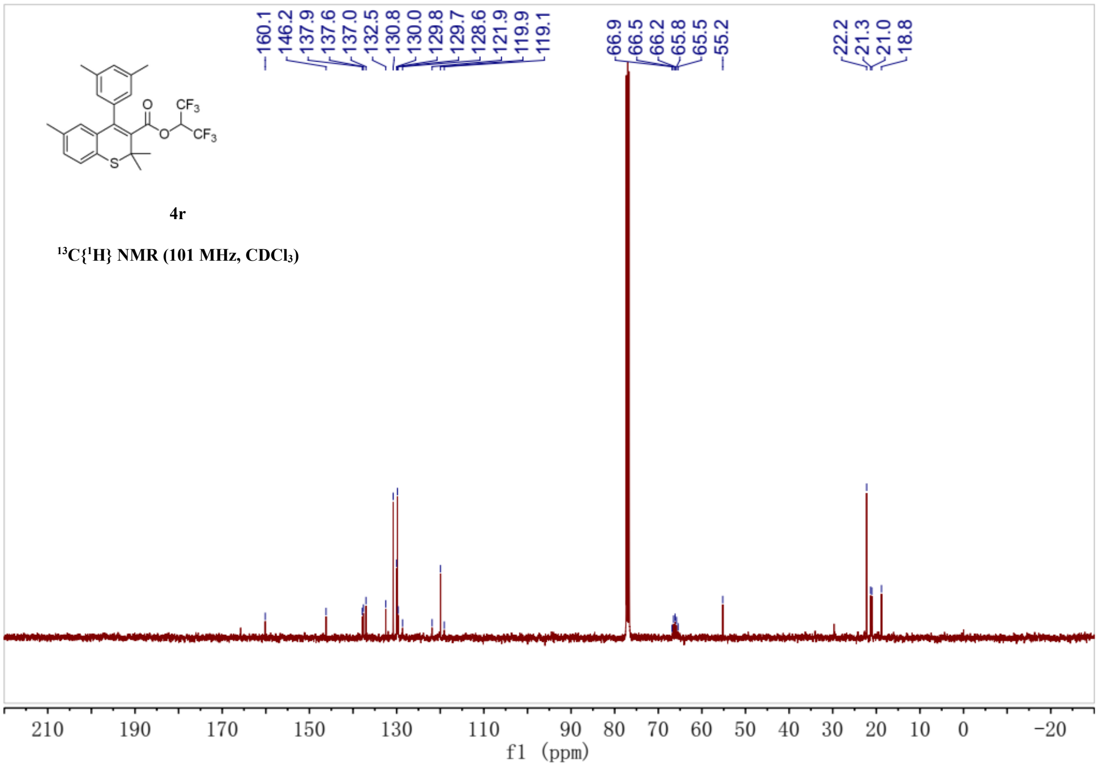

4r

^{13}C{^{1}H} NMR (101 MHz, $CDCl_3$)

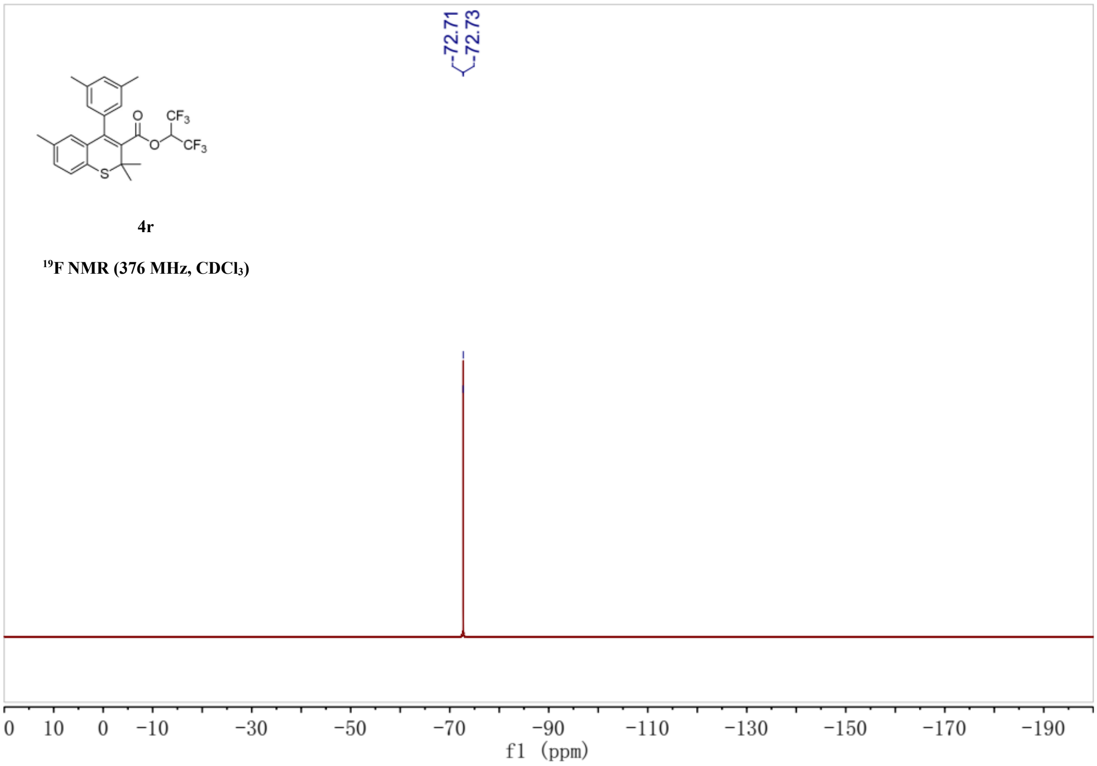

-72.71
-72.73
CF3
O
O
CF3
S
4r
19F NMR (376 MHz, CDCl3)
0
10
0
−10
−30
−50
−70
−90
−110
−130
−150
−170
−190
f1 (ppm)
